# Stress granules formed during infections with RNA viruses have significant virus-specific differences in generation and composition

**DOI:** 10.1371/journal.pbio.3003363

**Published:** 2026-08-14

**Authors:** Reto M. Lang, Silvio Steiner, Jenna Kelly, Anne-Christine Uldry, Pratik Dave, Sophie Braga-Lagache, Jeffrey Chao, Manfred Heller, G. Tuba Barut, Volker Thiel

**Affiliations:** 1 Institute of Virology and Immunology (IVI), Bern and Mittelhäusern, Switzerland; 2 Department of Infectious Diseases and Pathobiology (DIP), Vetsuisse Faculty, University of Bern, Bern, Switzerland; 3 Graduate School for Cellular and Biomedical Sciences, University of Bern, Bern, Switzerland; 4 Multidisciplinary Center for Infectious Diseases (MCID), University of Bern, Bern, Switzerland; 5 Department for BioMedical Research (DBMR), University of Bern, Bern, Switzerland; 6 Friedrich Miescher Institute for Biomedical Research (FMI), Basel, Switzerland; University of Wisconsin-Madison, UNITED STATES OF AMERICA

## Abstract

Eukaryotic cells evolved a cellular stress response to cope with extrinsic and intrinsic stress stimuli including virus infections. The major result of this response is the shutdown of bulk translation to prevent damage and allow the reprogramming of translation towards stress-resolving pathways. The resulting translationally stalled mRNA and associated proteins are accumulated in membrane-less cytosolic condensates called stress granules (SG). While the inhibitory effect of translation arrest on virus growth is well established, the role of SGs in the cellular defense against viruses is still unclear. The observation of specific interference with SG formation during various virus infections led to the hypothesis that SGs could serve as antiviral signaling platforms. In this study, we used mouse hepatitis virus (MHV) to characterize SGs formed during coronavirus infection. By applying APEX2-mediated proximity labeling in combination with quantitative proteomics, we dissected the proteome of MHV-induced granules and compared it to canonical SGs formed during oxidative stress. Our data revealed substantial differences in protein abundance and composition, indicating stressor-specific SG characteristics. To assess if the observed differences are a general feature of virus-induced SGs or rather virus-specific, we extended our investigations to the Semliki Forest virus (SFV), a member of the alphavirus family known to induce SGs. An initial comparison of SG formation kinetics by live-cell imaging showed distinct time points of SG induction between both viruses. A comprehensive comparison of the SG protein compositions revealed profound differences in the SG proteome between SFV and MHV. A further subcellular localization of SG components by microscopy not only confirmed a reduced abundance of several translation initiation factors in MHV-induced granules, but surprisingly, revealed the presence of SFV RNA and the absence of MHV RNA in virus-induced SGs. The reduced connection to canonical SG themes observed for MHV-induced granules compared to SFV- and oxidative stress-induced ones indicates a different impact of these condensates on MHV replication and further raises the question whether they should be considered SGs. The surprising plasticity of SGs concerning induction kinetics, protein composition and abundance, and inclusion or exclusion of viral RNA provide a base for future investigations of the role(s) of SGs in the context of viral infection and how they may impact virus replication.

## Abstract 2

Stress granules are thought to contribute to antiviral defense, but their roles during RNA virus infection remain unclear. This study shows that coronavirus and alphavirus infections generate stress granules with distinct kinetics, proteomes, and viral RNA content, highlighting virus-specific condensate plasticity, with potentially different impacts on viral replication.

## Introduction

Eukaryotic cells have evolved a stress response to cope with various intrinsic and extrinsic stress stimuli including virus infections. Besides a shutdown of bulk translation, this response is characterized by the formation of cytosolic stress granules (SGs). These highly dynamic condensates consist of translationally stalled mRNAs and various associated proteins including eukaryotic translation initiation factors (eIFs) [1,2]. The formation of SGs is mediated by specific RNA-binding proteins like the major SG markers Ras GTPase-activating protein-binding protein 1 (G3BP1), T-cell restricted intracellular antigen-1 (TIA-1), and ubiquitin associated protein 2 like (UBAP2L) by promoting a multivalent network of RNA and protein interactions [1,3,4]. Many studies have suggested the involvement of SGs in the antiviral response as a signaling platform. Several antiviral sensor proteins including the protein kinase R (PKR), were found to accumulate in SGs during different stress stimuli [5–7]. Furthermore, various viruses have been shown to interfere with the SG response by inhibiting their formation or manipulating their characteristics to favor viral replication [8,9]. However, the proposed function of SGs as an antiviral signaling platform was recently questioned, as several studies could not find antiviral sensor proteins in SGs and further the absence of SGs by G3BP1-KO did not alter antiviral signaling [10,11]. These contradictory results show that the role of SGs during virus infections is still poorly understood and might vary between viruses.

Aside from mRNAs, SGs accumulate a large variety of cellular proteins, which might modulate their function during different stress stimuli. To comprehensively identify the proteome of these highly dynamic condensates and capture transiently interacting proteins two studies previously used proximity labeling assays based on the biotin ligase BirA or the engineered peroxidase APEX2 [12,13]. APEX2 is fused to G3BP1 as SG bait and catalyzes the tagging of G3BP1 proximal proteins with biotin phenol (BP) during a short labeling period of 1 min [12]. Markmiller and colleagues applied APEX2-mediated proximity labeling in combination with quantitative mass spectrometry to compare the SG proteome during different nonviral stress situations including oxidative stress induced by sodium arsenite treatment [12]. Among the classical SG marker proteins, they identified many novel SG associated proteins. Furthermore, Markmiller and colleagues and others found that the protein composition of SGs differs between stress stimuli as well as cell types [1,12,13]. These observations demonstrate stress type specific characteristics of SGs suggesting an adaptation of these condensates to handle different stress conditions.

As the characteristics of SGs in the context of virus infections are still poorly understood, we set out to compare SGs induced by two different viruses, the Semliki Forest virus (SFV) and the mouse hepatitis virus (MHV). While many viruses prevent SG formation completely, the SFV induces SGs early in infection by activating the dsRNA sensor PKR [9,14]. This virus is a member of the alphavirus family possessing a positive-sense single stranded, capped, and polyadenylated RNA genome. Interestingly, SFV was shown to actively dissolve the granules later in infection and prevent their reformation [9,15]. The mechanism was suggested to be mediated by the interaction of nsP3 with G3BP1 and the subsequent sequestration of this SG core component [9,16]. However, a recent study questions this view by showing that a loss of the nsp3-G3BP1 interaction does not prevent SG disassembly, while SFV-induced transcription and translation shutoff seem to be essential [17]. Nevertheless, the formation of SG in infection and their subsequent disassembly by the virus, makes SFV a suitable model to study these granules in virus infections.

MHV is a member of the coronavirus family, which is characterized by an exceptionally long positive-sense single stranded RNA genome. Coronaviruses encode various nonstructural and accessory proteins, which are involved in the evasion of host defense mechanisms [18]. In the context of the stress response, it was recently found that the human coronavirus SARS-CoV-2, causative agent of the COVID19 pandemic, inhibit SG formation mediated by the interaction of the nucleocapsid protein (N) with G3BP1 [10,19]. Interestingly, this interaction motif in N is specific for SARS-CoV-2 and could not been found in other coronaviruses including MHV, which was previously shown to trigger SG formation during infection [20]. However, the role of these SGs during coronavirus infections as well as the general characteristics of virus-induced SGs is still poorly understood. Due to a possible role in the antiviral response, the insight into SGs induced by different viruses can provide valuable information on the host-virus interaction in the context of the stress response. This is of special importance for coronaviruses, as the recent COVID-19 pandemic demonstrated the risk posed by members of this virus family and the need to understand their interaction with the host cell.

Here we provide a detailed characterization of SGs in the context of virus infections. By comparing these condensates induced by two RNA viruses from the coronavirus and alphavirus families we observed substantial differences in the kinetics of SG formation. By applying APEX2-mediated proximity labeling, quantitative proteomics and different microscopy-based techniques, we report a remarkable plasticity of SGs concerning protein composition and abundance and concerning the inclusion or exclusion of viral RNA. Collectively, our data on various aspects of virus-induced SGs unravels virus-specific characteristics of these condensates that may impact their role(s) during virus infection.

## Results

### Generation of G3BP1-APEX2-GFP expressing cell line for microscopy-based investigations and proximity labeling

**Fig 1:**
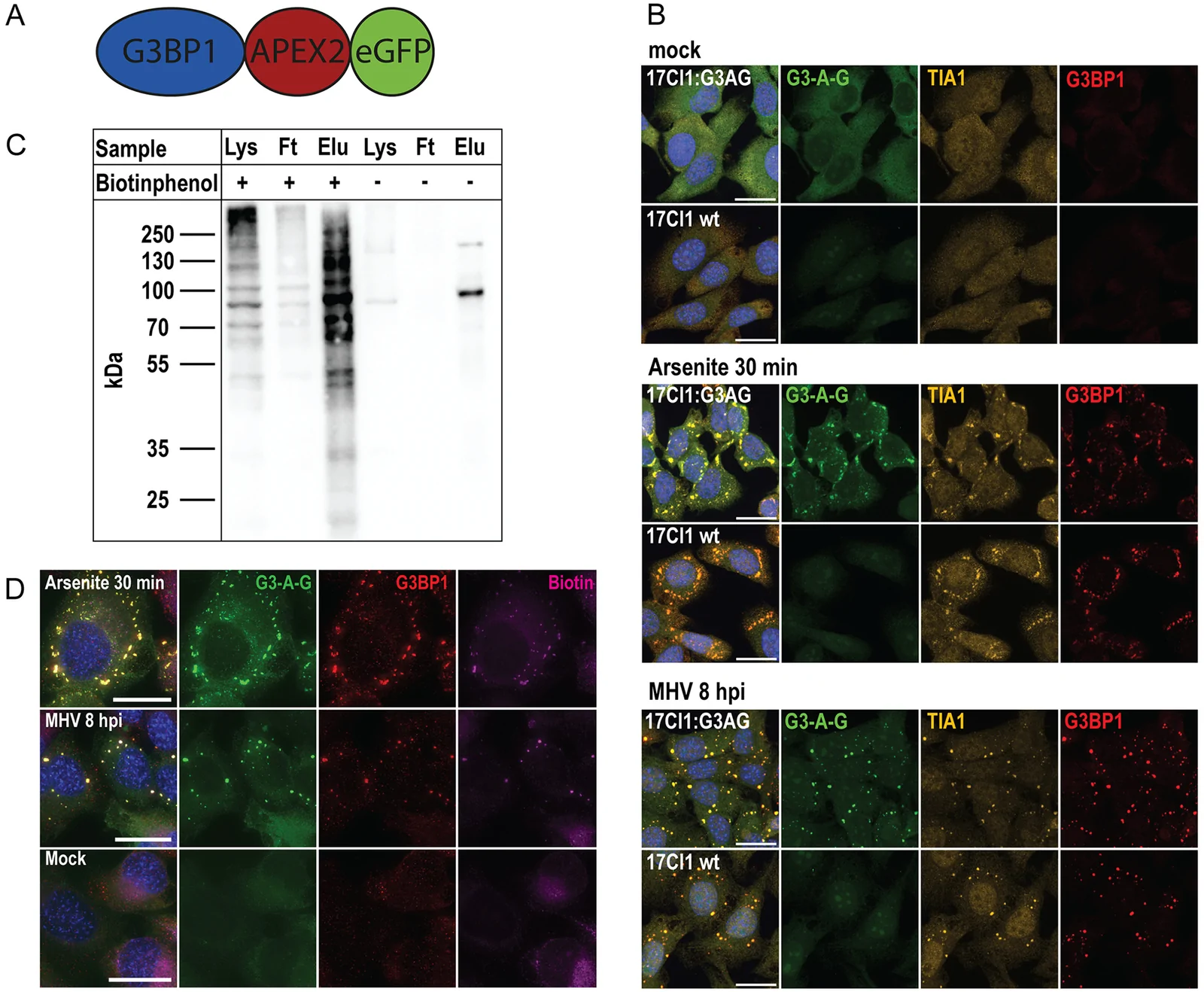
Cell line expressing G3BP1-APEX2-GFP fusion protein allows visualization and proximity-dependent biotinylation of SGs during oxidative and infection stress. **(A)** Schematic representation of the fusion protein consisting of the main SG marker G3BP1, the engineered APEX2 peroxidase for proximity labeling, and an eGFP tag for visualization of SGs by fluorescence microscopy. **(B)** Verification of SG formation and accumulation of G3-A-G fusion protein (green) in recombinant 17Cl1:G3-A-G cells compared to wildtype cells by immunofluorescence microscopy. 17Cl1:G3-A-G or wildtype cells were treated with 250 μM sodium arsenite for 30 min or infected with MHV-A59 (MOI 10). Control of unstressed (mock) cells was additionally included. Cells were fixed at the indicated time point after treatment/infection and an indirect immunofluorescence staining targeting TIA1 (orange) and G3BP1 (red) was performed. Nuclei were stained with DAPI (blue). Scale bar: 20 μm **(C)** Verification of APEX2-mediated proximity labeling of proteins by western blot analysis. 17Cl1:G3-A-G were incubated with 500 μM BP for 30 min. Then, proximity labeling reaction was started by adding hydrogen peroxide. After 1 min the reaction was quenched and cells were lysed. Affinity purification of labeled proteins was performed on streptavidin-coated magnetic beads. Lysates (Lys), flowthrough (Ft), and eluates (Elu) were analyzed for the presence of biotinylated proteins by western blot analysis. As negative controls, BP was omitted for the labeling reaction. **(D)** Verification of APEX2-mediated proximity labeling of SG components by immunofluorescence. Recombinant 17Cl1:G3-A-G cells (green) were either treated with sodium arsenite (250 μM, 30 min) or infected with MHV-A59 (MOI 5, 8 hpi) to induce G3-A-G granules. Unstressed cells (mock) were used as negative control. Syncytia formation in MHV infected cells was prevented by HR2 peptide addition at 2 hpi. Cells were incubated with BP 30 min before the labeling time points. At the indicated time points, labeling reaction was started by adding hydrogen peroxide. Reaction was quenched after 1 min and cells were fixed. An immunofluorescence staining with NeutrAvidin (magenta) to visualize biotinylated proteins, and an antibody targeting the SG marker G3BP1 (red) was performed. Scale bars: 20 μm.

To investigate SGs formed during infection with MHV, we first generated a recombinant mouse fibroblast 17Cl1 cell line stably expressing a fusion protein of the SG marker G3BP1, the engineered ascorbate peroxidase APEX2, and an eGFP tag, hereafter referred to as G3-A-G (Fig 1A). While the eGFP enables microscopy-based characterization of SG formation, the APEX2 enzyme is used for proximity-dependent biotinylation allowing dissection of the G3BP1 microenvironment.

First, we confirmed the accumulation of fusion protein in SGs upon different stress stimuli. We treated 17Cl1:G3-A-G cells with sodium arsenite, a known inducer of oxidative stress [21], or infected them with MHV strain A-59. For infections with MHV, we supplemented the medium with HR2 peptide. This prevents syncytia formation and allows analysis of single cells, especially important for imaging. The subcellular distribution of fusion protein during presence of SGs was assessed by fluorescence microscopy. Based on a preliminary experiment with wildtype cells, we selected 8 hpi as time point of interest for MHV-induced SGs (S1 Fig). We used antibody staining for SG markers G3BP1 and TIA1 to visualize SGs [1]. In the unstressed state (mock) the fusion protein is diffusely distributed in the cytosol as visible in Fig 1B (top panel). A weaker signal in the G3-A-G channel can also be observed for the wildtype cells, which is caused by autofluorescence of the sample. Upon stress induced by either arsenite treatment or MHV infection, G3-A-G accumulates in cytosolic foci, which overlap with the staining for G3BP1 and TIA1 (Fig 1B, middle/bottom panel). These accumulations have a similar appearance to the G3BP1/TIA1 positive SGs formed in the wildtype cells. Based on these results we conclude that G3-A-G localizes to SGs during stress.

Proximity-dependent labeling based on the engineered peroxidase APEX2 has proven an important method to comprehensively identify the proteome of cellular organelles, like mitochondria, as well as more dynamic subcellular structures including SGs [12]. To verify the activity of APEX2, we performed a proximity labeling assay in 17Cl1:G3-A-G cells with a 1 min labeling time after 30 min of incubation with or without BP and further performed affinity purification of labeled proteins. The presence of biotinylated proteins in the lysates, flowthrough and eluates was assessed by western blot analysis. The lysate of 17Cl1:G3-A-G cells incubated with BP contains many different biotinylated proteins as indicated by the numerous distinct bands and the diffuse staining distributed over the whole mass range (Fig 1C, first lane). The affinity purification of this sample shows a successful enrichment of labeled proteins in the eluate (Fig 1C, 3rd lane). In contrast, lysate omitted with BP shows only two distinct bands and no diffuse staining (Fig 1C, 4th lane). Taken together, these results verify APEX2-mediated biotinylation of proteins in our fusion protein expressing cells.

We further analyzed the subcellular localization of biotinylated proteins by fluorescence microscopy to verify the labeling of SG components. MHV-infected or sodium arsenite treated 17Cl1:G3-A-G cells were proximity labeled and fixed at 8 hpi and 30 min treatment respectively and stained with fluorophore-conjugated neutravidin. The results revealed a strong accumulation of biotinylated proteins in MHV- and arsenite induced SGs, verifying the labeling of SGs (Fig 1D).

Taken together, our data showed that the G3-A-G fusion protein localizes to SGs upon different stress stimuli. Furthermore, the APEX2 enzyme contained in the fusion protein efficiently catalyzes the proximity-dependent biotinylation of SG proteins.

### MHV-induced SG reveal a distinct proteome from canonical SG

**Fig 2:**
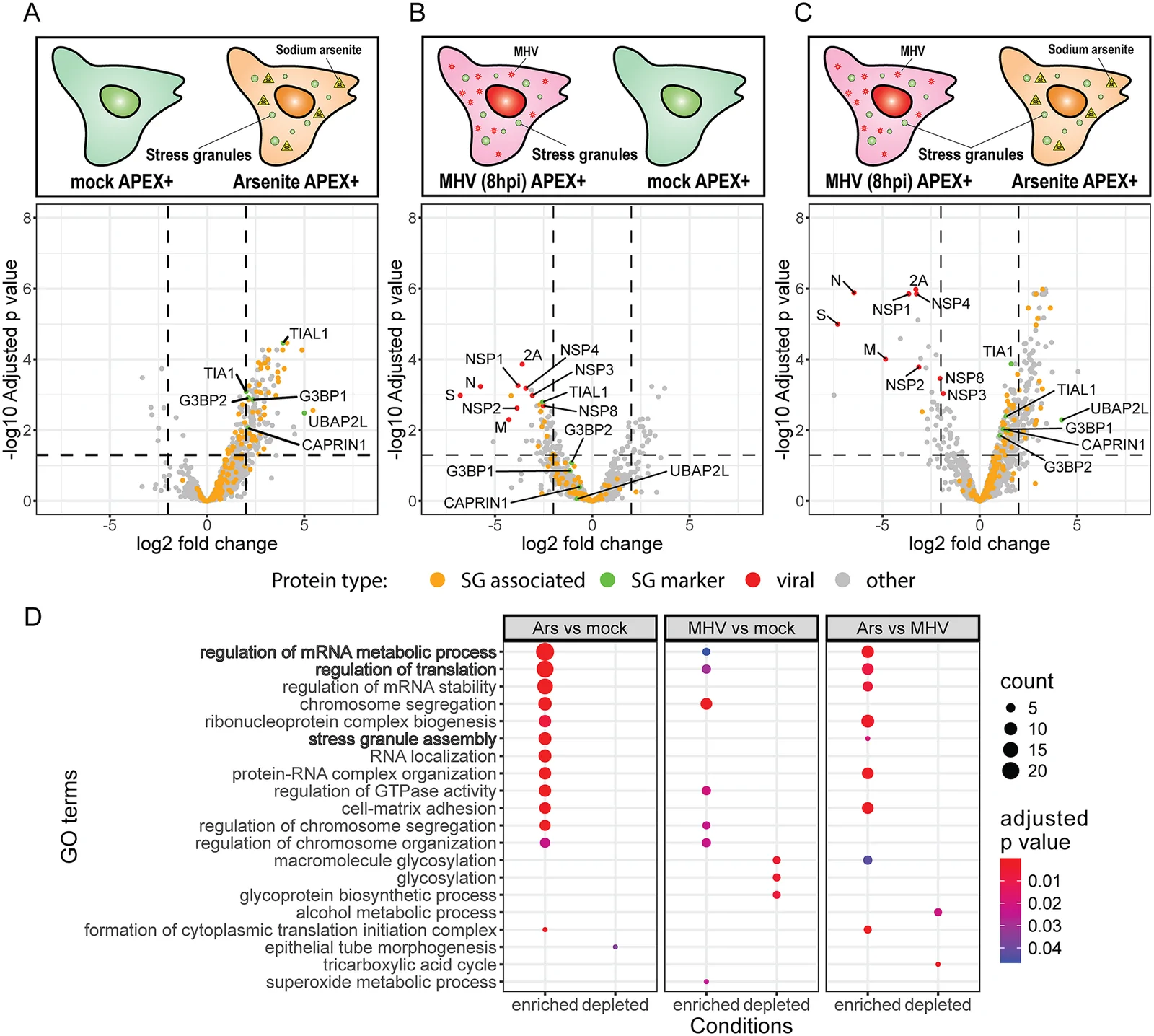
Comparing the protein composition of the SG microenvironment during oxidative stress and MHV infection using APEX2-mediated proximity labeling and quantitative proteomics. Protein composition of the G3-A-G microenvironment was compared between **(A)** sodium arsenite treated and unstressed cells, **(B)** MHV-infected and unstressed cells, as well as **(C)** a direct comparison of the two stressors, sodium arsenite treatment and MHV infection. Volcano plots show the mean log2 fold change between the compared conditions (shown in scheme) for all identified proteins against the corresponding –log10 adjusted *p* value. Proteins from the SG associated proteins reference list (RNA granule database) are indicated in orange, main SG marker proteins are labeled and indicated in green, and viral proteins are labeled and indicated in red. The selected significance levels for log2 fold change (≥2 and ≤−2) and adjusted *p* value (<0.05) are shown with dashed lines. **(D)** GO term enrichment analysis for biological processes was performed on the fraction of significantly enriched/depleted proteins for each comparison using ClusterProfiler in RStudio. Redundant terms were reduced by semantic similarity using the rrvgo package. The circle size indicates the number of genes identified, the color gradient indicates the adjusted *p* value (based on Benjamini-Hochberg). Top 5 terms for each condition based on the adjusted *p* value are displayed. Interesting terms in the context of SGs are highlighted in bold. The data underlying this Figure can be found in S2 Table and S1 Data.

After verifying the 17Cl1:G3-A-G cells, we applied APEX2-mediated proximity labeling to comprehensively dissect the proteome of MHV-induced SGs and compare it to canonical SGs induced by sodium arsenite. We combined this technique with stable isotope labeling of amino acids in cell culture (SILAC) allowing a direct comparison of two conditions by quantitative mass spectrometry based on 'heavy’ or ‘light’ stable isotope labeled amino acids (lysine and arginine) in the culture medium (S2A Fig). We designed five individual comparisons, each of which consists of two different conditions, whereby one is carried out on ‘heavy’ cells and one on ‘light’ cells (S2B Fig). Our setup consists of a direct comparison of MHV- and arsenite-induced SGs as well as a comparison of each stressor with the unstressed condition (Fig 2A–2C) and a stressed but unlabeled control. To capture the SG proteome, the labeling reaction was performed at 8 hpi for MHV infection and 30 min after sodium arsenite treatment, respectively. A total of 2,206 different proteins were detected over all analyzed samples, including 164 SG associated proteins based on a list of known SG proteins derived from the RNA granule database (S1 and S2 Tables) [22].

**Fig 3:**
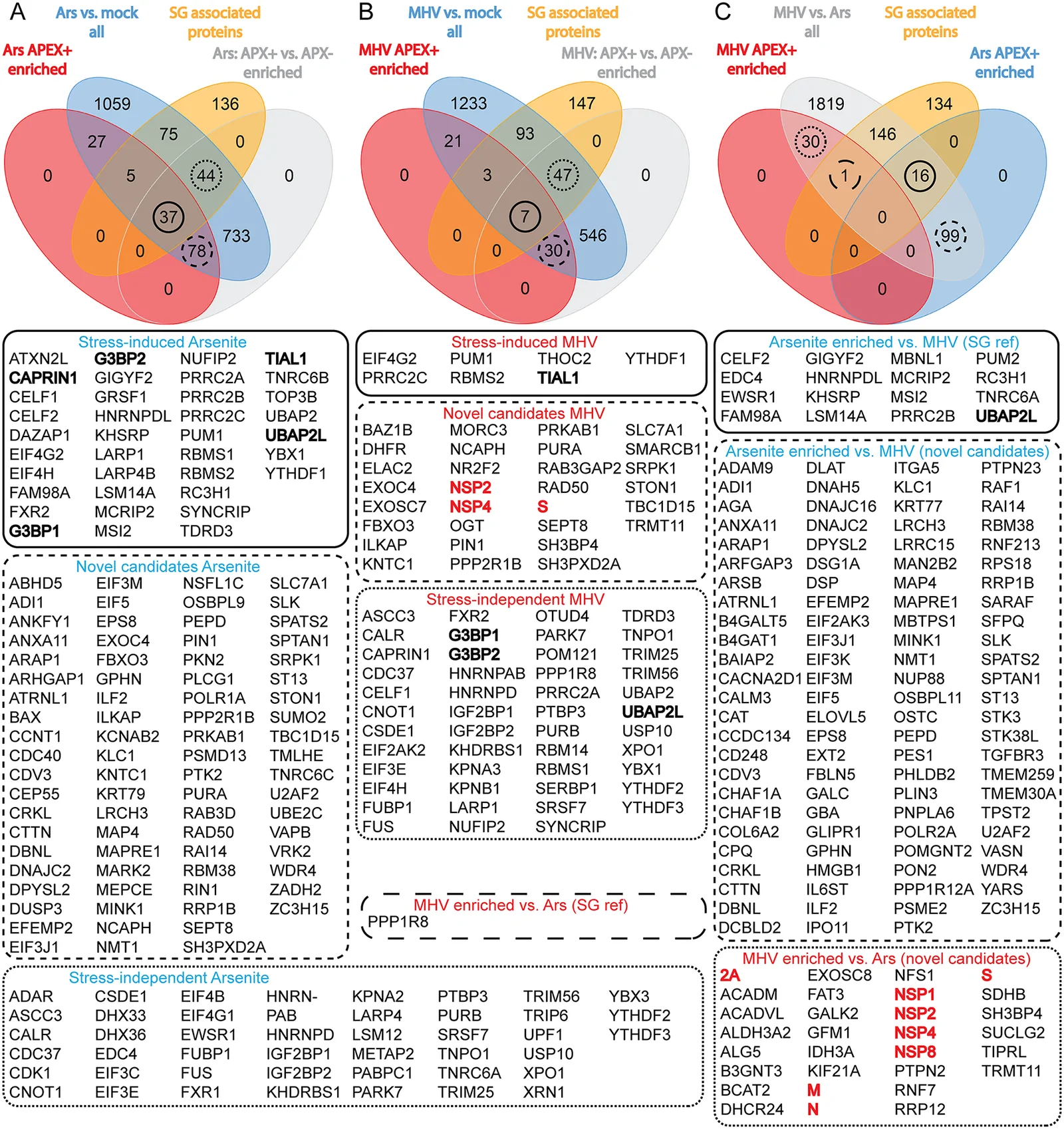
Comparison of SG proteome during oxidative stress and MHV infection. Comparison of SG microenvironment between **(A)** oxidative stress induced by sodium arsenite treatment and the unstressed state (mock) or **(B)** MHV infection and the unstressed state (mock). Different subsets of stress-dependent and independent SG proteins are determined using a Venn diagram between the subset of significantly enriched proteins for the stressor (Ars/MHV enriched, red), all identified proteins of the same comparison (blue), the subset of significantly enriched proteins for the proximity labeled condition (APEX+, gray) in the negative labeling control comparison, and a reference list of known SG associated proteins (orange). Proteins of specific subsets, including stress induced known SG proteins (solid line), novel candidates of SG proteins for respective stress stimulus (large dashes) and stress independent known SG proteins (small dashes), are highlighted with circles and corresponding lists of proteins are provided below. **(C)** Venn diagram shows overlap between significantly enriched proteins for arsenite treatment (blue), significantly enriched proteins for MHV infection (red) and all identified proteins in the direct comparison between these stressors (gray) with the SG associated proteins reference list (orange). Subsets of arsenite specific SG proteins (solid line), arsenite specific novel SG protein candidates (large dashes) and MHV specific novel SG candidates (small dashes) are highlighted with circles and corresponding protein lists are provided below. Main SG marker proteins are highlighted in bold, viral proteins in bold and red. For all significantly enriched fractions log2 fold change significance level of ≥2 and ≤−2, and an adjusted *p* value significance level of <0.05 were used.

We analyzed the data using a similar approach to Markmiller and colleagues [12]. An initial analysis of heavy/light ratios revealed substantial differences in the proteome of MHV- and arsenite-induced SGs. The second analysis based on individual samples, additionally considering proteins unique for one condition, further strengthened this observation. The comparison of arsenite induced SGs with the unstressed state (mock) shows a shift of many SG associated proteins towards the arsenite condition as seen in volcano plot Fig 2A. Among the 147 significantly enriched proteins for arsenite stress based on our stringent significance criteria (log2(fold change) >1), we identify 37 SG associated proteins, which were also enriched in the APX+ versus APX−− labeling control condition and thus represents proteins with a strongly increased abundance in the G3BP1 microenvironment upon stress (stress-induced). These include the well-characterized SG nucleators G3BP1, G3BP2 [23,24], TIA1, TIAL [1,25], CAPRIN1 [24,26], and UBAP2L [4,12,24], as well as several components of the translation initiation complex (Fig 3A). Additionally, our screen identified 78 proteins enriched in arsenite-induced SGs which are not contained in our SG reference list and thus might represent novel candidates of oxidative stress induced SGs. Interestingly, we detected 119 known SG associated proteins, which did not meet our significance criteria. Of these proteins, 44 were also enriched in the labeling control and represent potential stress-independent G3BP1 interactors. Gene ontology enrichment analysis for biological processes of the significantly enriched proteins showed an enrichment for translation initiation and SG assembly, supporting the successful labeling of the SG microenvironment (Fig 2D). Overall, this experiment shows a strong stress induction by sodium arsenite treatment leading to a change in G3BP1 microenvironment in line with canonical SG formation.

In the comparison of the G3BP1 microenvironment between MHV-induced SGs and the unstressed mock cells, we observed the significant enrichment of 61 proteins for MHV. Interestingly, only 7 known SG associated proteins have been detected among these hits with TIAL1 as only SG nucleator protein (Figs 2B and 3B). Although additional 140 SG associated proteins have been detected, 47 of which are labeling control enriched, they show no significant change in abundance during MHV infection. Furthermore, although the GO terms “Regulation of mRNA metabolism” and “translation” still show up among the most significant terms, their significance is reduced (Fig 2D). Besides the SG associated proteins, we detected 51 significantly enriched proteins for MHV with no previous link to SG biology, 30 of which are also significantly enriched for the control condition (MHV APX+ versus MHV APX−) (Fig 3B, S3). Besides cellular proteins, these factors also include 9 viral proteins (nsp1, nsp2, nsp3, nsp4, nsp8, 2a, M, S, and N) (Fig 2B). Interestingly, our experiment revealed the significant depletion of 24 proteins from the G3BP1 microenvironment of MHV-infected cells. According to the GO term enrichment analysis, several of these proteins are associated with glycosylation (Fig 2D, middle). This posttranslational modification is known to be involved in cell signaling and was also previously linked to SG biology [27]. As it was suggested that SGs might function as a signaling platform in the antiviral response [6,28], we checked for the presence of classical RNA sensors like RIG-I, MDA5, and PKR in infected/treated samples. However, none of these factors have been detected in the labeled SG proteome.

For the direct comparison between MHV and sodium arsenite induced stress, we observed enrichment of most SG associated proteins for arsenite-induced SGs similar to the mock comparison, although the fold change of most of the SG nucleator proteins dropped below our significance criteria (Fig 2C). Among the 115 significantly enriched proteins for arsenite, 16 have been identified as known SG associated factors (Fig 3C). In contrast, only 31 proteins are significantly enriched for MHV compared to arsenite, with PPP1R8 as only previously SG associated protein (Figs 2C and 3C). Similar to the previous comparison, the MHV enriched SG proteome shows the presence of several viral proteins. Our analysis suggests that the arsenite induced stress response is stronger than the one induced by MHV. This is supported by the GO term enrichment analysis showing an enrichment for terms connected to mRNA metabolism, translation, and SG formation for arsenite stress against MHV, however, to a lesser extent than observed in the comparison with unstressed cells (Fig 2D). In contrast, the MHV infected condition, which also showed some enrichment for these terms in the previous mock comparison, is lacking their enrichment compared to arsenite stress.

Taken together, our proximity labeling screen showed the expected enrichment of SG associated proteins in the microenvironment of sodium arsenite induced SGs in line with previous findings. In contrast, the microenvironment of SGs during MHV infection revealed a decreased abundance of SG associated proteins and a general reduction in the protein variety. The two observed stress stimuli thus induce a distinct SG composition.

### SG are induced at distinct time points during MHV and SFV infection

**Fig 4:**
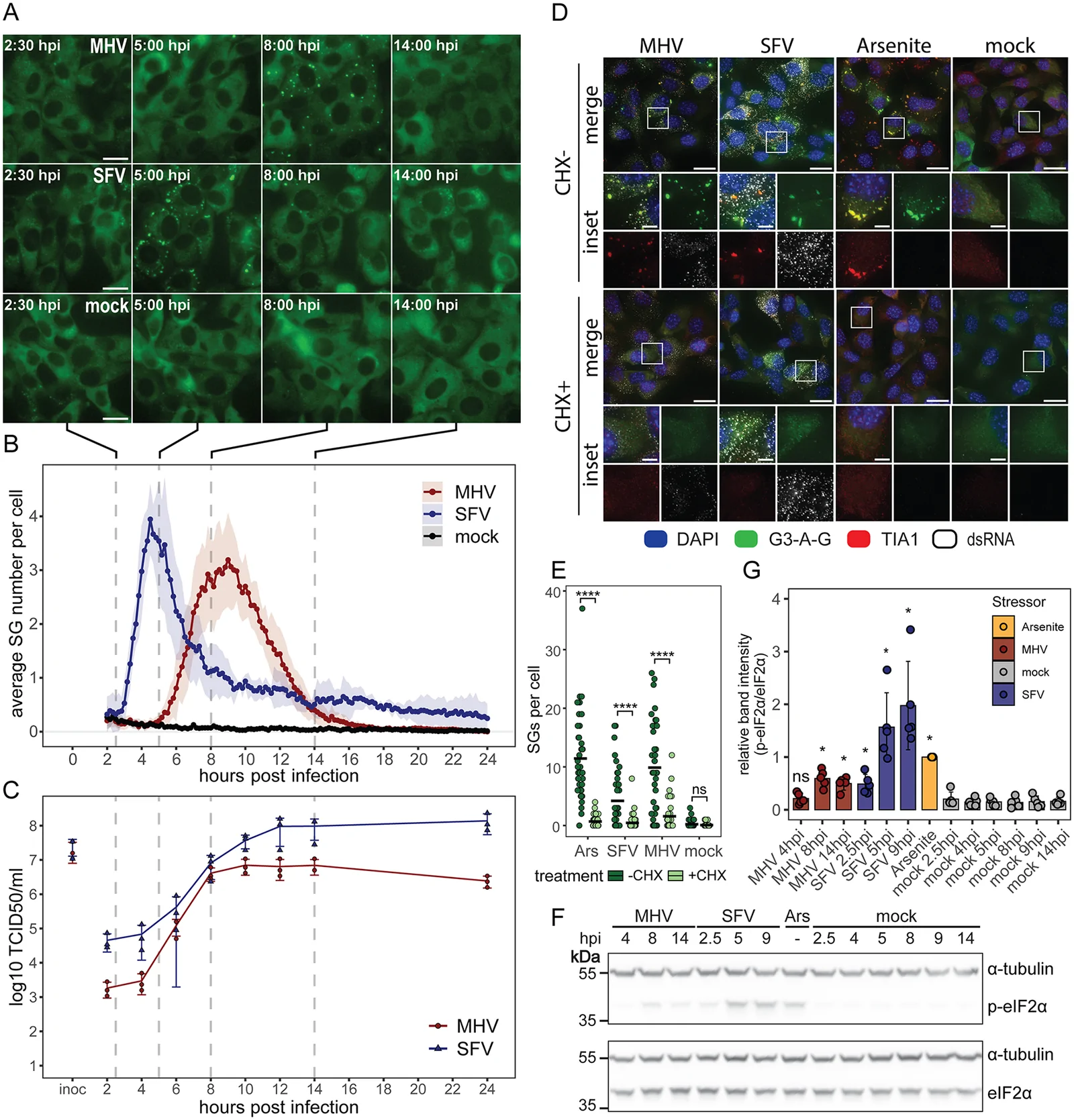
Analysis of SG formation during MHV and SFV infections by live-cell imaging. **(A)** 17Cl1 cells expressing a G3BP1-APEX2-GFP fusion protein (G3-A-G, green) were infected with either MHV or SFV at an MOI of 10, or mock infected. MHV infected samples were additionally supplemented with HR2 peptide to prevent syncytia formation. Cells were imaged every 10 min for ~22 h on a DeltaVision widefield fluorescence microscope with on-stage incubator (see S1 Movie). The panel shows a selection of time points. **(B)** Quantification of average SG number per cell based on live-cell imaging data. SGs were segmented and counted using Ilastik and Fiji. Number of SGs per frame was standardized with the number of visible cells and the mean based on four separate image series for each condition was plotted in RStudio. The ribbon indicates the standard deviation. **(C)** Single cycle infection kinetics for MHV and SFV on 17Cl1:G3-A-G cells. Cells were infected with MHV or SFV at an MOI of 10. Supernatants were collected at the indicated time points and virus titers were estimated by TCID50 assay. (*n* = 3). **(D)** Effect of cycloheximide (CHX) on SGs during MHV and SFV infection or sodium arsenite treatment assessed by indirect immunofluorescence staining. 17Cl1:G3-A-G cells were either infected with MHV-A59 or SFV at an MOI of 10, or treated with sodium arsenite (250 μM) to induce SGs. MHV infected samples were supplemented with HR2 peptide to prevent syncytia formation. CHX+ samples were treated with 50 μM cycloheximide 1.5 h prior to fixation. Cells were fixed at 8 hpi (MHV), 5 hpi (SFV), or 30 min after sodium arsenite treatment. Cells were stained with antibodies against TIA1 (red) and dsRNA (grays) and imaged on a DeltaVision widefield fluorescence microscope. Scale bar: 20 μm or 5 μm (inset). **(E)** Quantification of SGs per cell for each stress condition with and without cycloheximide treatment based on 4–6 IF images per condition from two independent replicates. Each dot represents a cell. Statistical significance between treated and untreated samples for each condition was tested by Wilcoxon signed-rank test. **(F)** Level of phosphorylated eIF2α at indicated time points during MHV and SFV infection assessed by western blot analysis. 17Cl1:G3-A-G cells were infected at an MOI of 10, mock infected or treated with sodium arsenite. Lysates were collected at indicated time points and western blot analysis targeting phosphorylated eIF2α, or eIF2α was performed; α-tubulin was used as loading control. **(G)** Quantification of phosphor-eIF2α (Ser51) level based on Western blots of five independent replicates. Plot shows the mean and individual p-eIF2α/ eIF2α ratios for each condition normalized with the positive control (Ars). SD is indicated by error bars. Band intensities were measured in Fiji, loading corrected based on loading control. Statistical significance between each infected/treated condition and the corresponding mock control (mock 5 hpi for Arsenite) was tested by pairwise Wilcoxon signed-rank test and Benjamini-Hochberg FDR correction. ****: *p* < 0.0001; *: *p* < 0.05; ns: nonsignificant. The data underlying this Figure can be found in S1 Data.

The profound differences in stress granule proteomes between MHV infection and sodium arsenite treatment raised the question if the reduced abundance of several SG markers is a general feature of virus-induced SG or rather an MHV- or coronavirus-specific occurrence. Thus, we extended our investigation to SFV, which was previously used in several studies to investigate SG in the context of virus infection and thus serving as a reference [9,15,29]. First, we assessed the stress granule dynamics during MHV and SFV infection. For this purpose, we infected 17Cl1:G3-A-G cells with either MHV or SFV at a high multiplicity of infection (MOI = 10) and repeatedly imaged the samples for up to 22 hpi. For both infections, the formation of cytoplasmic accumulates of G3-A-G could be observed, although the time point of their appearance substantially differs between the viruses (Fig 4A, S1 Movie). The quantification of the average SG number per cell displayed in Fig 4B shows a rapid SG formation after 3 hpi for SFV. The maximum SG number is reached between 4 and 5 hpi, followed by a fast disassembly of the condensates. At 8 hpi almost all SGs disappeared, and no reformation can be observed for the rest of the experiment. This is in line with previous studies reporting the presence of SGs early in SFV infection and their subsequent disassembly [9,15]. For MHV infection, the SGs start to appear much later at 5 hpi, reaching a peak at 8 hpi as observed previously. Like for SFV, MHV-induced granules dissolve with progressing infection and almost no SGs can be seen after 14 hpi. This delayed induction of SGs is in line with a previous study showing the presence of MHV-induced granules only after 6 hpi [20].

The SG induction time point might be dependent on the duration of the virus replication cycle. Thus, we next performed a one cycle replication kinetics for both viruses. 17Cl1 cells were infected at an MOI of 10 and the amount of newly formed infectious particles was measured at different time points. Both viruses have comparable replication kinetics showing an exponential release of new infectious particles after 4 hpi before reaching a plateau within 12 hpi (Fig 4C). Correlating the SG formation and replication kinetics, we find that the induction of SGs occurs early in SFV infection before the exponential growth and release of new virus. In contrast, MHV shows the SG formation at a later stage of infection simultaneously with its exponential growth phase. Interestingly, the granules persist during the whole exponential phase of MHV growth and are only dismantled once the viral titer reaches a plateau (Fig 4B, 4C). It was shown for several viruses that they form viral aggregates with SG components to interfere with the cellular stress response [8,30,31]. To assess if MHV might form aggregates of SG markers disconnected from SGs, we performed cycloheximide treatment of infected cells. This drug stalls the ribosomes on the mRNA causing the disassembly of canonical SGs, which are in an equilibrium with polysomes, a feature not observed for the viral aggregates [25,32]. Without treatment we observe the formation of G3-A-G positive granules containing TIA1 in the majority of cells for all three stress stimuli as seen previously (Fig 4D, 4E). In contrast, when cells are treated with cycloheximide a strong reduction in the SG number per cell can be observed for the canonical stressor arsenite, as well as for both viruses (Fig 4D, 4E). Based on these results, MHV- and SFV-induced granules are in equilibrium with polysomes like canonical SGs contradicting the viral accumulations in MHV infection.

The major pathway of canonical SG induction is the integrated stress response (ISR). For SFV it is well known that ISR is activated early in infection leading to SG formation [15]. To investigate, if SG formation during MHV infection occurs together with ISR activation, we next analyzed the level of phosphorylated eIF2α (p-eIF2α), the main marker for ISR activation, at different time points during MHV and SFV infection. We further used sodium arsenite treatment as a positive control for canonical stress. The western blot analysis reveals greatly increased levels of p-eIF2α for all SFV time points, whereby the later time points exceed the level of arsenite stress (Fig 4F, 4G). A small increase in p- eIF2α level can also be seen for MHV at later time points indicating the activation of ISR (Fig 4F, 4G). However, it is substantially lower than our positive control.

Collectively, our data showed the formation of SG-like condensates during MHV and SFV infections, which are in equilibrium with the translation machinery similar to canonical SGs. Simultaneously with SG appearance, both viruses induce the activation of the ISR, although MHV shows a much weaker activation compared to SFV and arsenite. Furthermore, the induction time point of these condensates in relation to the replication cycle differs strongly between the viruses.

### G3BP1 microenvironment diverges between MHV and SFV infected cells during SG formation

**Fig 5:**
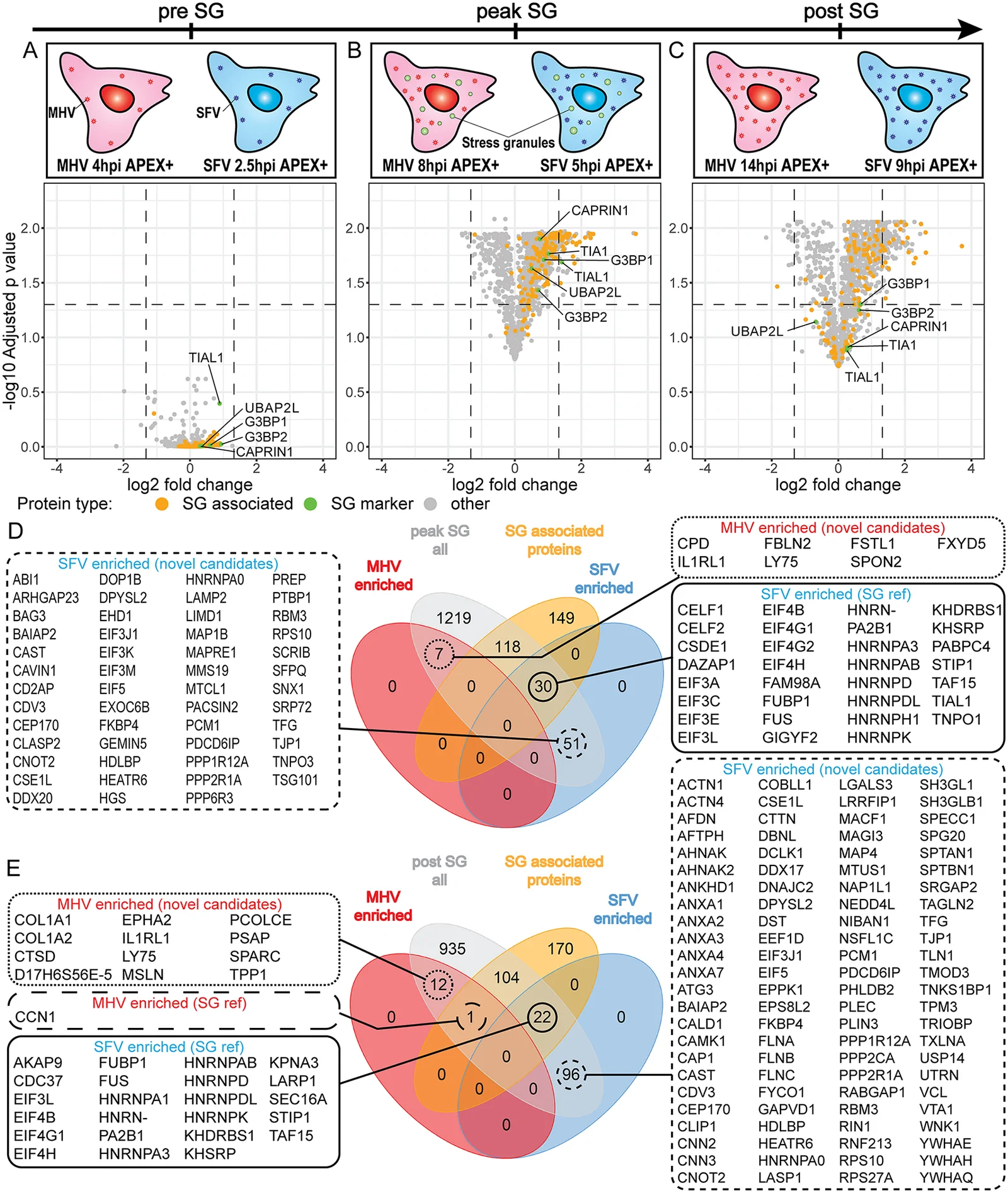
Direct comparison of the G3-A-G microenvironment between MHV and SFV infection at different time points. Scheme visualizing the timeline and the compared conditions. **(A)** Different abundance of proteins between MHV-infected (4 hpi) and SFV-infected (2.5 hpi) and proximity labeled cells prior to SG formation. **(B)** Different abundance of proteins between MHV-infected (8 hpi) and SFV-infected (5 hpi), proximity labeled cells at the respective peak SG time point. **(C)** Different abundance of proteins between MHV-infected (14 hpi) and SFV-infected (9 hpi), proximity labeled cells after SG disappearance. Volcano plots display the mean log2 fold change based on the heavy/light protein ratio between the conditions (*n* = 3) plotted against the -log10 adjusted *p* value. Proteins contained in the reference list for SG associated proteins are indicated in orange, the major SG markers are labeled and indicated in green. Dashed lines show the significance criteria for log2 fold change > log2(2.5) and <-log2(2.5), respectively, and adjusted *p* value < 0.05. Venn diagrams visualizing overlap between significantly enriched protein fractions (red: MHV/blue: SFV), all identified proteins (gray) and reference list of SG associated proteins (orange) for **(D)** the peak SG time point and **(E)** the time point after SG disappearance (post SG). For selected sections the corresponding lists of proteins are provided. The data underlying this Figure can be found in S3 Table and S1 Data.

Next, we comprehensively compared the SG proteome between MHV and SFV infection to assess if previously observed characteristics of MHV-induced SGs are also observed for other viruses. We again applied APEX2-mediated proximity labeling in combination with SILAC. To control for virus-induced changes in G3BP1 microenvironment in the absence of SGs, the labeling reaction was conducted at three different time points in relation to SG formation. Thereby, time points before SG induction (pre SG, Fig 5A), at SG peak (peak SG, Fig 5B) and after SG disassembly (post SG, Fig 5C) were selected for MHV and SFV and directly compared between both viruses. The proximity labeling was verified by immunofluorescence and western blot analysis (S4 Fig). Furthermore, the labeling control condition showed a strong enrichment of most proteins for the unstressed proximity labeled condition compared to the unlabeled condition in line with a successful proximity labeling (S5A). We detected 3,502 different proteins over all analyzed samples, 216 of which are SG associated proteins based on our reference list (S3 Table). Hereafter we focus primarily on proteins detected in both compared conditions, thus yielding a heavy/light ratio.

For the comparison of the pre SG time point between MHV and SFV we detected 2013 proteins in both conditions, whereby 175 were previously associated with SGs including several major SG markers (Fig 5A, S5C). Interestingly, no significant differences in protein abundance can be observed between the viruses based on our significance criteria (log2(2.5) and p 0.05). These results indicate that the G3BP1 microenvironment seems to be similar in both virus infections prior to SG formation.

In contrast to the pre SG time point, we detected substantial changes in the G3BP1 microenvironment between MHV and SFV at the peak of SG presence. We found 81 proteins to be significantly enriched for SFV-induced granules compared to MHV (Fig 5B). Over a third of these proteins [30] belong to our list of SG associated proteins indicating a strong connection to SG biology of the SFV-enriched fraction (Fig 5D). Furthermore, we found a tendency of most SG associated proteins, including the major SG markers, to be enriched for SFV compared to MHV as seen in the volcano plot and the log2 fold change density distribution of SG associated proteins (Fig 5B, S5B). In contrast, only 7 proteins are significantly enriched for MHV-induced granules compared to SFV, none of which was previously associated with canonical SGs (Fig 5B, 5D). Based on these results, SFV-induced granules match very well to canonical SG components while the MHV-induced ones show substantial differences. These findings are in line with an additional SILAC experiment directly comparing SFV-, MHV-, and arsenite-induced SGs with unstressed mock cells (S4 Table). Similar to arsenite-induced SGs, the granules formed during SFV infection show an enrichment for known SG associated proteins including the major markers G3BP1/2 (S6A, S6C, S7A, S7C Figs). For MHV we can observe a high log2(FC) enrichment for the major SG nucleator (S6B, S7B Figs). But the majority of SG associated proteins show a lower fold change. Furthermore, the analysis shows a higher uncertainty as none of the proteins surpasses the *p* value criteria <0.05. These results indicate a less profound change of the G3BP1 microenvironment compared to the unstressed state.

After SG disassembly (post SG time point), the microenvironment of G3BP1 is still characterized by differences in protein composition between MHV and SFV infection (Fig 5C). However, despite an increased number of significantly enriched proteins for SFV compared to the peak SG time point, the number of SG associated proteins (22/118) decreased in line with the disassembly of SGs (Fig 5C, 5E). As many of these factors do not overlapping with the enriched fractions at the peak time point**,** the differences in G3BP1 microenvironment might represent general virus-specific changes in the cellular state or the accumulation of G3BP1 in other structures. For both viruses, we detected viral proteins foremost in the peak and post SG conditions (S8 Fig). MHV showed the same set of proteins detected in the previous SILAC experiment. For SFV, all nonstructural proteins have been detected with nsP3 as the most highly enriched one, which is in line with its G3BP1 interaction [9,30].

Based on the Venn diagrams, we further investigated subsets of significantly enriched proteins for the peak and post time points provided as protein lists in Fig 5D, 5E. Among the significantly enriched SG associated proteins (overlap with SG reference) for SFV peak SG we identified two dominant groups of proteins, the first consisting of several translation initiation factors (eIF) subunits and the second consisting of different members of the heterogeneous nuclear ribonucleoproteins (hnRNP) family (Fig 5D). Interestingly, many of the hnRNP members are amongst the most strongly enriched hits for SFV with a fold change up to 8 for FUS (Fig 5B).

**Fig 6:**
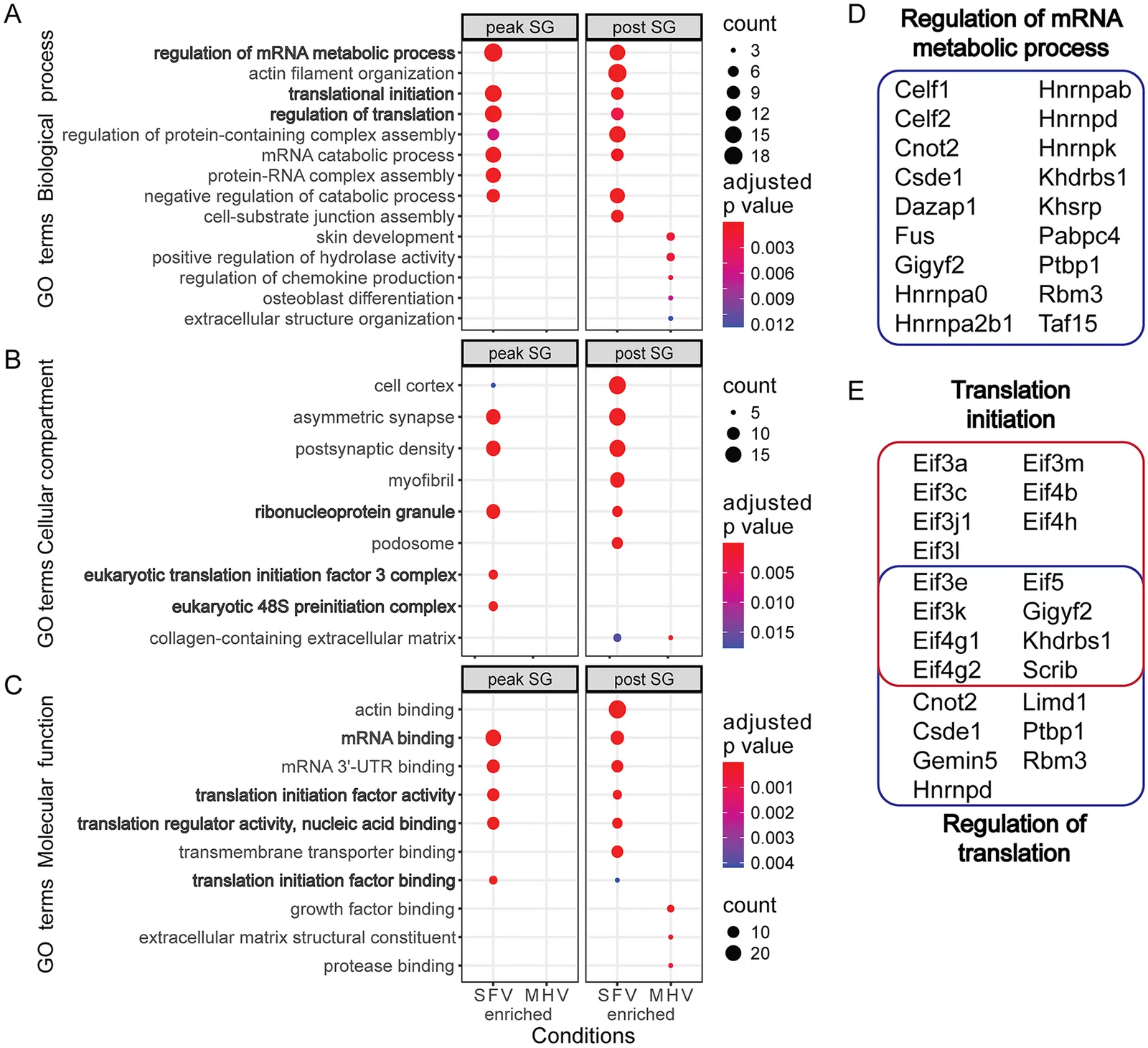
Functional analysis of significantly enriched/depleted proteins for SFV- vs. MHV-induced SGs. Subsets of significantly enriched proteins for SFV and MHV at the peak and post SG time points based on the direct comparison (|log2FC| > log2(2.5), adjusted *p* value < 0.05) were subjected to a GO term enrichment analysis using ClusterProfiler. Redundant terms for each condition were reduced based on semantic similarity. Displayed are the top 5 most significant terms found in each subset of proteins for **(A)** biological processes, **(B)** cellular compartment, and **(C)** molecular function. The circle size indicates the number of identified proteins for each term. Color indicates adjusted *p* value based on Benjamini–Hochberg. Terms of special interest in the context of SGs are highlighted in bold. Lists of proteins associated with the biological process **(D)** regulation of mRNA metabolic process, and **(E)** translation initiation, regulation of translation identified in this screen. The data underlying this Figure can be found in S3 Table and S1 Data.

To functionally classify the sets of significantly enriched proteins in the peak and post comparisons, we performed a GO term enrichment analysis. The enriched protein fraction for SFV at the SG peak time point revealed “regulation of mRNA metabolic process” and “translation initiation” as most significant terms for biological process (Fig 6A). A closer look at the associated proteins for both terms showed the two previously mentioned groups of hnRNPs and eIFs as predominant proteins (Fig 6D, 6E). Consistently, the analysis for cellular compartment (CC, Fig 6B) and molecular function (MF, Fig 6C) revealed a strong link to ribonucleoprotein granules, translation initiation factor 3 complex, translation regulation activity and mRNA binding. Most of these GO terms are also found for the SFV post SG enriched proteins (Fig 6A–6C). However, their associated count is reduced, which is in line with the reduced number of eIFs and hnRNPs enriched for the SFV post SG condition (Fig 5E). For MHV we only found a few significantly enriched proteins for peak and post time points strongly limiting the meaningfulness of the GO term enrichment analysis for these samples (Fig 6A–6C).

These results provide a detailed insight into the G3BP1 microenvironment at different time points during MHV and SFV infections by directly comparing both viruses. Prior to SG formation the G3BP1 microenvironment is similar for both viruses. When SGs are present, we observe substantial virus-specific differences characterized by an enrichment of many SG associated proteins including several members of eIFs and hnRNPs for SFV-induced SGs compared to MHV. Virus-specific differences in G3BP1 microenvironment also persist after disassembly of SGs. Overall, in the direct proteome comparison between MHV- and SFV-induced SGs as well as the comparisons with mock, SFV reveals an increased connection to canonical SG biology.

### Reduced abundance of specific SG markers in MHV-induced SGs compared to SFV

**Fig 7:**
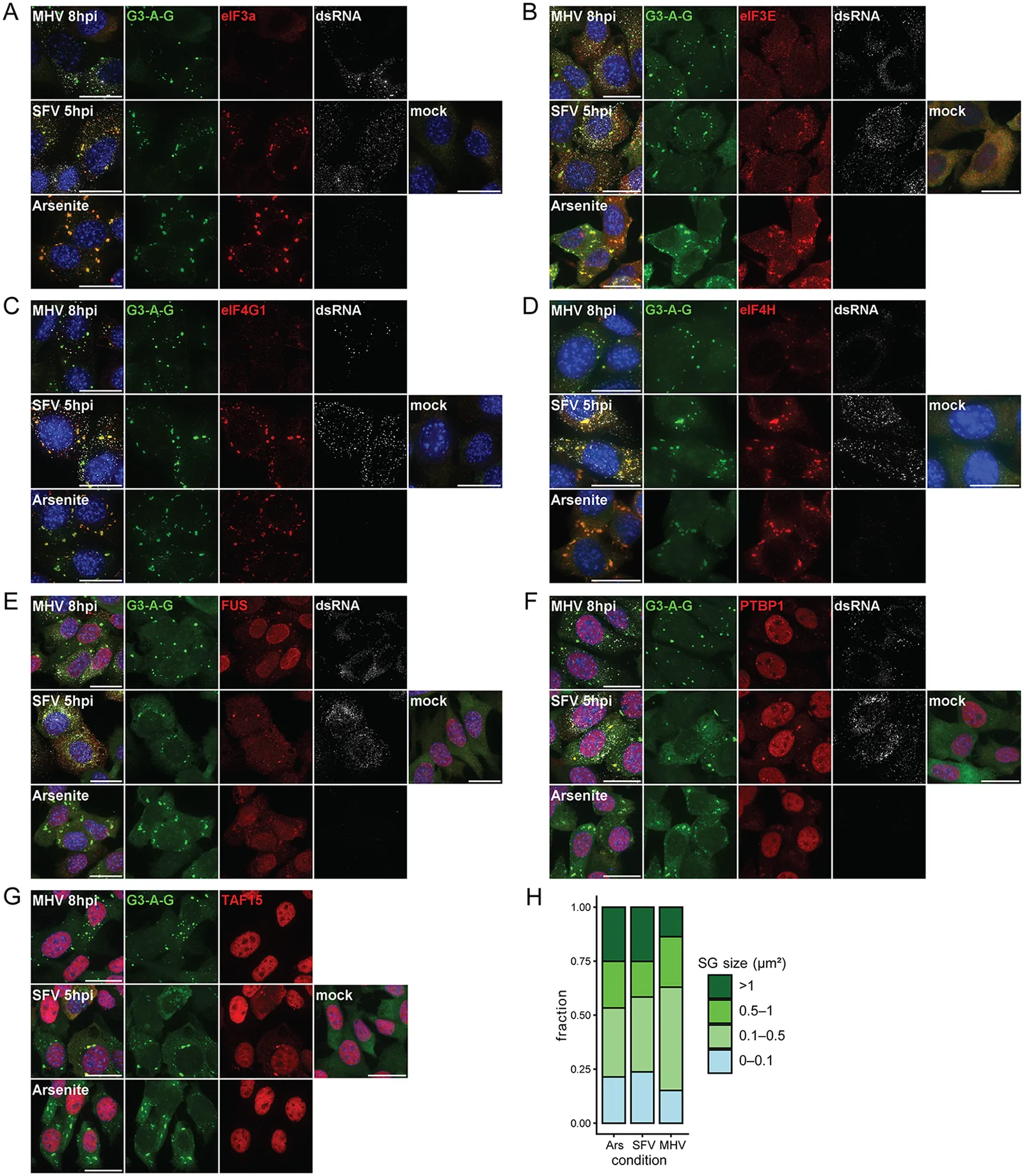
Subcellular localization of SG proteins during different virus infections or oxidative stress by indirect immunofluorescence. 17Cl1:G3-A-G cells (green) were infected with either MHV or SFV at an MOI of 10, mock infected or treated with 250 μM sodium arsenite for 30 min. Cells were fixed at the respective peak time point of SGs (5 hpi for SFV and 8 hpi for MHV) and subjected to indirect immunofluorescence staining. Staining of nucleus with DAPI (blue). **(A)** Staining of eIF3a (red) and dsRNA (grays). **(B)** Staining of eIF3L (red) and dsRNA (grays). **(C)** Staining of eIF4G1 (red) and dsRNA (grays). **(D)** Staining of eIF4H (red) and dsRNA (grays). **(E)** Staining of FUS (red) and dsRNA (grays). **(F)** Staining of PTBP1 (red) and dsRNA (grays). **(G)** Staining of TAF15 (red). All MHV-infected samples were supplemented with HR2 peptide to prevent syncytia formation. Images were acquired on a DeltaVision widefield system (A/C) or a Nikon spinning disk confocal system (B/D/E/F/G) and maximum intensity projection over Z stacks was performed in Fiji. Scale bar: 20 μm. **(H)** Quantification of SG size based on fluorescence microscopy images (all shown IF experiments). SGs were assigned to the indicated size categories based on the measured area, and the fraction of each category among the total SG count for each condition was plotted. The data underlying this Figure can be found in S1 Data.

Next, we aimed to verify selected proteins identified as significantly enriched for SFV-induced SGs by investigating their subcellular localization in SFV and MHV infected cells at the SG peak time point. Therefore, we applied indirect immunofluorescence microscopy on 17Cl1:G3-A-G cells staining for several eIFs (eIF3a, eIF3e, eIF4G1, and eIF4H) and proteins involved in mRNA processing (FUS, PTBP1, and TAF15). Additionally, we also stained double-stranded (ds) RNA as an intermediate of viral replication to visualize the presence of the viruses. Besides MHV and SFV infections, sodium arsenite treatment was included as reference for canonical SGs. All investigated translation initiation factors showed a strong accumulation in G3-A-G positive granules for the sodium arsenite treated samples, while they remained diffusely distributed in our mock control (Fig 7A–7D, bottom row). A similar accumulation of eIFs and colocalization with G3-A-G positive granules can be observed in cells infected with SFV (Fig 7A–7D, middle row). In contrast, the probing for eIFs in MHV infected cells only shows a weak accumulation of these proteins in G3-A-G positive granules (Fig 7A–7D, top row). A quantification of the signal intensities for eIFs in SGs verified the reduced level observed for MHV (S9A–S9D Fig). Thus, the previously observed difference in the abundance of eIFs between MHV- and SFV-induced SGs suggests a mechanism for exclusion or reduction of these proteins from MHV-induced granules, while the SFV-induced SGs are similar to canonical SGs induced by arsenite. The staining for the major SG markers, G3BP1, TIA1, and UBAP2L, as well as eIF4A3 revealed a similar accumulation for all investigated SGs (S10 Fig). The staining for specific hnRNP members revealed a different picture. While a weak accumulation of FUS, PTBP1 and TAF15 can be observed in arsenite and MHV-induced G3-A-G granules, the majority of these proteins still localizes to the nucleus similar to the mock control (Fig 7E–7G). For SFV infected cells, we found two different phenotypes. Some cells show a predominant localization of FUS, PTBP1 and TAF15 in the nucleus with only a weak accumulation in SGs if present. Some cells, however, display an even distribution of these proteins throughout the whole cell, which comes together with a stronger accumulation in virus-induced SGs (Fig 7E–7G). This strong accumulation of nuclear proteins was especially pronounced for PTBP1 and TAF15 as seen in the quantifications of signal intensity in SGs (S9E–S9G Fig). Many cells with delocalization of FUS to the cytoplasm also display an intense signal for dsRNA, indicating a progressed stage of infection. To further verify this observation, we performed a time course experiment staining SFV-infected cells at different time points after infection. The number of cells with cytoplasmic localization of nuclear proteins increases later in infection together with an increased dsRNA signal (S11–S13 Figs).

Based on the IF images, we further determined the SG size by segmenting the SGs using the G3-A-G channel and measuring the area. Figure 7H displays the fraction of SGs assigned to different size categories for each condition. While for arsenite- and SFV-induced granules roughly a quarter reaches a size larger than 1 μm^2^, for MHV only 12% falls into the largest category indicating a tendency of MHV to induce smaller SGs.

When considered together, the subcellular localization of various proteins by indirect immunofluorescence microscopy verified the virus-specific differences in SG protein composition. We confirmed the difference in abundance of several eIFs in MHV-induced granules while the SFV-induced ones are similar to canonical SGs. In contrast, the enrichment of several hnRNPs seems to be specific for SFV-induced SGs and is likely connected to delocalization of these proteins with progressing infection. This observation highlights another virus-specific characteristic of the investigated granules.

### MHV only produces low levels of dsRNA in infected cells

**Fig 8:**
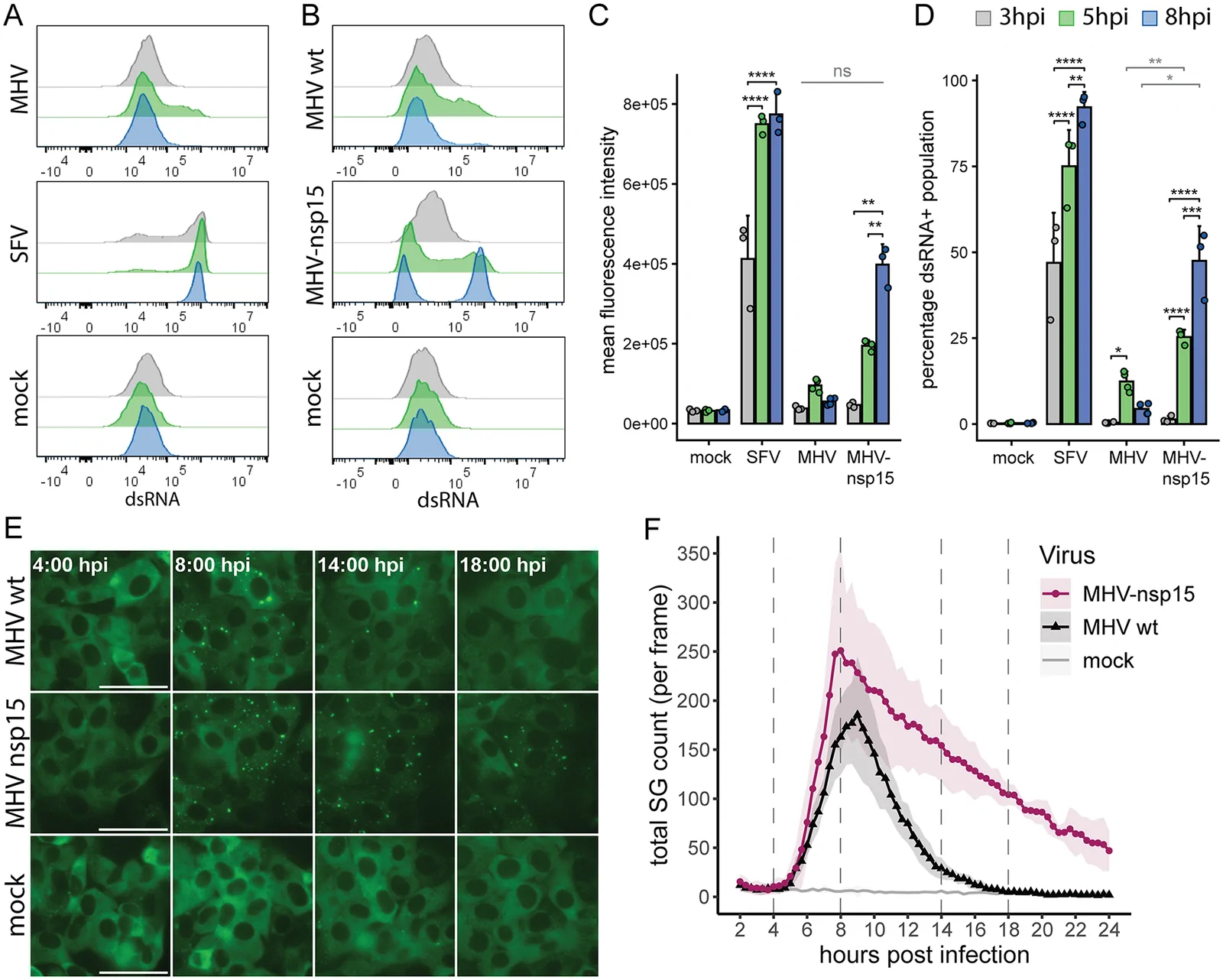
Substantially lower level of dsRNA in MHV infected cells compared to SFV, which is partially mediated by nsp15 endonuclease. Assessment of dsRNA levels in **(A)** MHV and SFV or **(B)** MHV-nsp15 and MHV wildtype infected cells at different time points. 17Cl1 wildtype cells were infected with MHV or SFV at an MOI 10, or MHV-nsp15 at MOI 5, or mock infected and collected at indicated time points. Cells were stained for intracellular dsRNA using anti-dsRNA J2 antibody and intensity was measured by flow cytometry. Graphs show the recorded fluorescence intensity histograms of the dsRNA staining of a representative replicate. **(C)** Mean dsRNA intensity values or **(D)** percentage of dsRNA^+^ cells and corresponding standard deviations for each condition and time point based on three to four independent replicates. Significance between time points (black) and between MHV and MHV-nsp15 (gray) was tested by Tukey's multiple comparisons test in GraphPad. ****: *p* < 0.0001; ***: *p* < 0.001; **: *p* < 0.01; *: *p* < 0.05; ns: nonsignificant. **(E)** SG formation during infection with MHV-nsp15 investigated by live-cell imaging (see S2 Movie). 17Cl1:G3-A-G cells (green) were infected with MHV-nsp15 at MOI 10 and imaged up to 24 hpi on a widefield fluorescence microscope with on-stage incubator. Scale bar: 50 μm. **(F)** SG number per frame over time based on live-cell imaging of MHV-nsp15, MHV wildtype or mock infected 17Cl1:G3-A-G cells. The graph displays the mean SG count based on four image series per condition; the ribbon indicates the standard deviation. The data underlying this Figure can be found in S1 Data and on the Zenodo repository (https://doi.org/10.5281/zenodo.21507492).

Compared to the strong stressor arsenite as well as SFV, we only observed a low level of ISR activation during MHV infection indicating that MHV only causes a weak stress stimulus, which could explain the observed reduction in many canonical SG proteins. A major driver of ISR activation during virus infection is the sensing of dsRNA in the cytoplasm, which was shown to be the cause of a robust ISR activation during SFV infection [15]. Thus, we wondered if differences in the dsRNA levels during MHV and SFV infections could give rise to the different intensity of stress response and SG formation. We collected MHV- or SFV-infected 17Cl1 cells at three different time points after infection (3–8 hpi), stained for intracellular dsRNA using an anti-dsRNA antibody and assessed the dsRNA intensity by flow cytometry. Indeed, while SFV shows a substantial fraction of cells with increased dsRNA levels compared to mock already at 3 hpi, which further increases to over 80% at 8 hpi, MHV reveals a substantially smaller fraction of cells with high dsRNA levels at any investigated time point (Fig 8A, 8C, 8D, S14A, S14B). To our surprise, the fraction of high dsRNA^+^ cells is the highest at 5 hpi and strongly decreases at 8 hpi (Fig 8A, 8D). Coronaviruses are well known to employ different strategies to counteract immune sensing. One crucial factor is the nsp15 endonuclease, which was shown to degrade viral dsRNA to subvert its detection by cytoplasmic sensors including PKR [18,33]. Thus, we repeated the dsRNA level measurement with an MHV mutant (H277A) with impaired nsp15 nuclease activity [18]. In line with the inactive endonuclease, we observed increased dsRNA levels at all investigated time points and most strikingly no decrease but an increase in dsRNA at 8 hpi (Fig 8B–8D, S14C). This result highlights the role of nsp15 in maintaining a low abundance of dsRNA during MHV infection. When we investigated the formation of SGs during MHV-nsp15 infection by live-cell imaging, we found that the impaired nsp15 function did not change the induction time point of the granules but increased their number and altered their disassembly leading to a prolonged persistence of SGs (Fig 8E, 8F, S2 Movie).

Combined, our assessment of dsRNA levels during MHV and SFV infections showed a striking difference between both viruses highlighting the elaborate strategy of coronaviruses to maintain low dsRNA levels to prevent or weaken the induction of the stress response.

### MHV genomic and subgenomic RNA is not accumulated in SGs

**Fig 9:**
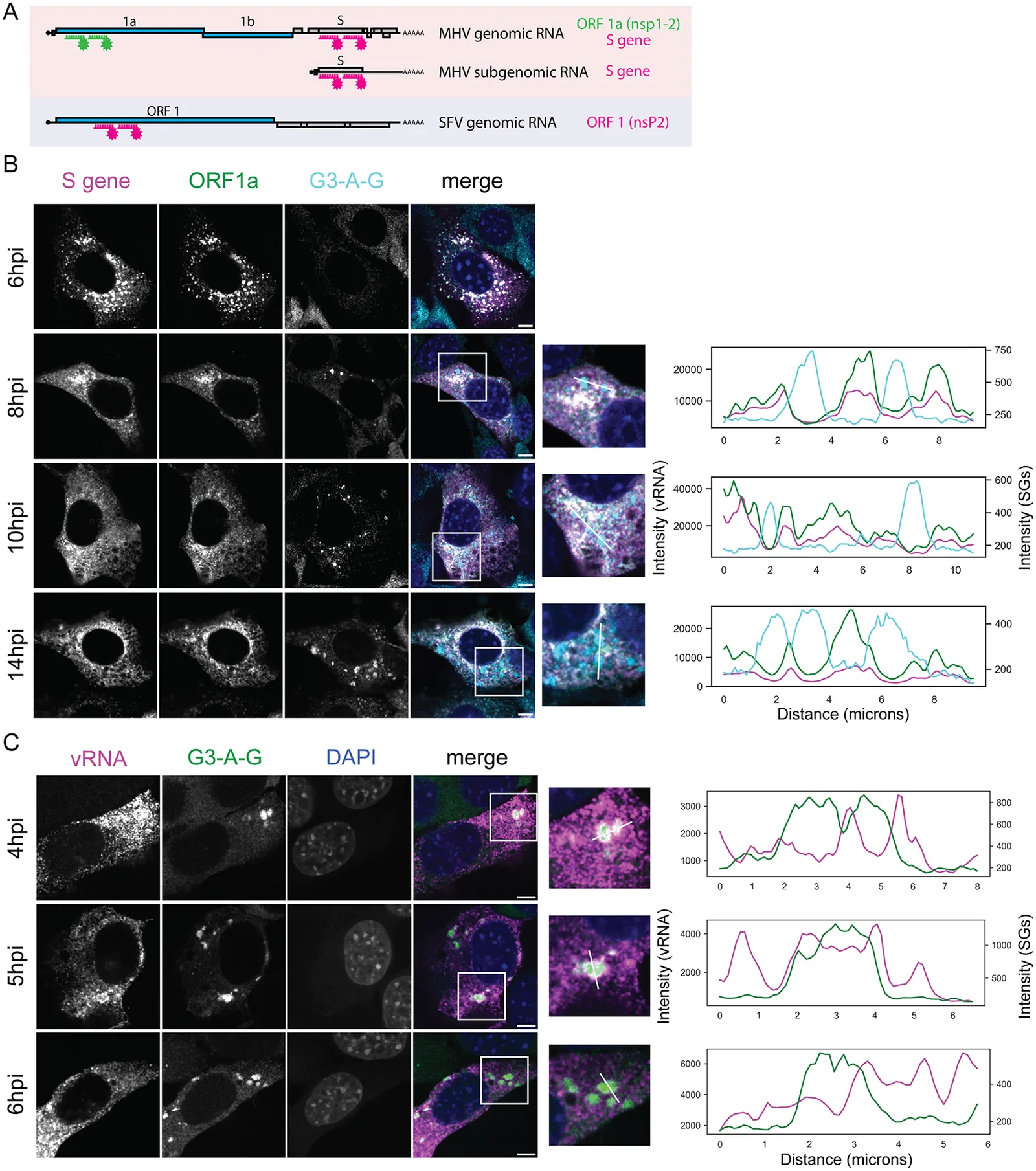
Subcellular localization of viral RNA in relation to SGs during MHV and SFV infection using smRNA-FISH. **(A)** Scheme indicating the different probes and their binding location within the MHV or SFV genome or subgenomic RNA. To visualize genomic and subgenomic RNA of MHV two different probes were designed, each consisting of a pool of 35 different, fluorophore-conjugated oligonucleotides complementary to either the nsp1/2 genes or the S gene. For staining of SFV genomic RNA a probe targeting the nsp2 gene was designed. **(B)** Visualization of MHV genomic (double positive) and subgenomic (positive for S probe) RNA in relation to SGs at different time points after infection. 17Cl1:G3-A-G cells (cyan) were infected with MHV-A59 at an MOI of 0.1. After infection, medium was supplemented with HR2 peptide to prevent syncytia formation. Cells were fixed at the indicated time points and stained for viral RNA using probes targeting the nsp1/2 genes (green) and the S gene (magenta). **(C)** Visualization of SFV genomic RNA in relation to SGs at different time points after infection. 17Cl1:G3-A-G cells (green) were infected with SFV (MOI 0.1) and fixed at indicated time points. FISH staining was performed using probe (magenta) complementary to SFV nsP2 gene. All images were acquired with a Leica spinning disk confocal microscope and post-processed in Fiji. Intensity line plots display the measured signal intensity of the different probes and the G3-A-G along the white line indicated in the inset. Scale bar: 5 μm. The data underlying this Figure can be found in S1 Data.

Besides proteins, RNA is a major component of SGs and it was previously observed that not only cellular mRNA but also viral mRNA can accumulate in these condensates [5,34]. Thus, we wondered if viral mRNA is accumulated in SGs induced by MHV or SFV. To assess the subcellular distribution of viral RNA, we applied single molecule RNA fluorescence in situ hybridization (smRNA-FISH). This technique relies on a pool of fluorescently labeled probes complementary to the sequence of interest allowing single molecule resolution [35]. To visualize MHV mRNA we designed two different probes. One targets the nsp1 and nsp2-coding sequence in the ORF1a and thus only detects full length viral genomes (Fig 9A). The second probe binds the sequence of the spike protein staining full length genomic RNA and subgenomic mRNA of the spike gene. Both probes in combination allow to distinguish between genomic and subgenomic RNA of MHV (Fig 9A). For SFV we designed one probe targeting the ORF1 (nsP2-coding region) to detect full length genomic RNA (Fig 9A).

17Cl1:G3-A-G cells were infected with either MHV or SFV and different time points around the previously used SG peak time point were selected for both viruses to be analyzed. The resulting images for MHV can be seen in Fig 9B. An intense signal for both vRNA probes can be observed at all time points. At 6 hpi well defined foci in the perinuclear region can be observed, which are positive for both probes, indicating genomic viral RNA. These foci likely represent the replication organelles. In all later time points, the staining for vRNA becomes more evenly distributed throughout the cytoplasm (Fig 9B). Due to the high amount of vRNA present at all investigated time points no individual RNA molecules can be resolved. G3-A-G positive cytoplasmic foci are present in infected cells of all but the 6 hpi time point. Interestingly, the G3-A-G fusion protein shows an inverse correlation with both viral RNA probes for all cells with SGs. This is evident in the intensity line plots (Fig 9B), revealing absence of vRNA at sites of high G3-A-G signal representing SGs. A staining for an endogenous mRNA, the activating transcription factor 4 (ATF4) previously shown to accumulate in SGs [36], revealed that in contrast to the viral RNA cellular mRNA is readily detectable in MHV-induced SGs (S15 Fig). These findings indicate that MHV genomic and subgenomic mRNAs are excluded from virus-induced SGs.

Similar to MHV, cells infected with SFV show the presence of large amounts of viral genomic RNA in the cytoplasm for all investigated time points (Fig 9C). In contrast to MHV, genomic RNA of SFV is not excluded from virus-induced SGs at all imaged time points. Even more, the viral RNA seems to have accumulated in the G3-A-G positive foci. As visible in the intensity line plots, this accumulation concentrates particularly at the edge of the SGs (Fig 9C). We further verified the observed differences in vRNA accumulation between MHV and SFV by combining smRNA-FISH with immunofluorescence staining of TIA1 as second SG marker. The resulting images and fluorescence quantifications confirm MHV vRNA exclusion and SFV vRNA accumulation in SGs (S16 Fig). Collectively, the subcellular localization of viral RNA in relation to SGs revealed virus-specific differences in SG characteristics between MHV and SFV infection also on the RNA level. While for SFV the genomic viral RNA is accumulated mainly at the edges of virus-induced SGs, MHV revealed an efficient exclusion of its genomic and subgenomic mRNAs from these condensates. Overall, our investigation into various aspects of SG formation during MHV and SFV infection showed that these condensates are largely characterized by the type of virus inducing them.

### MHV does not actively suppress SG formation induced by arsenite or SFV but prevents accumulation of its vRNA in SGs

**Fig 10:**
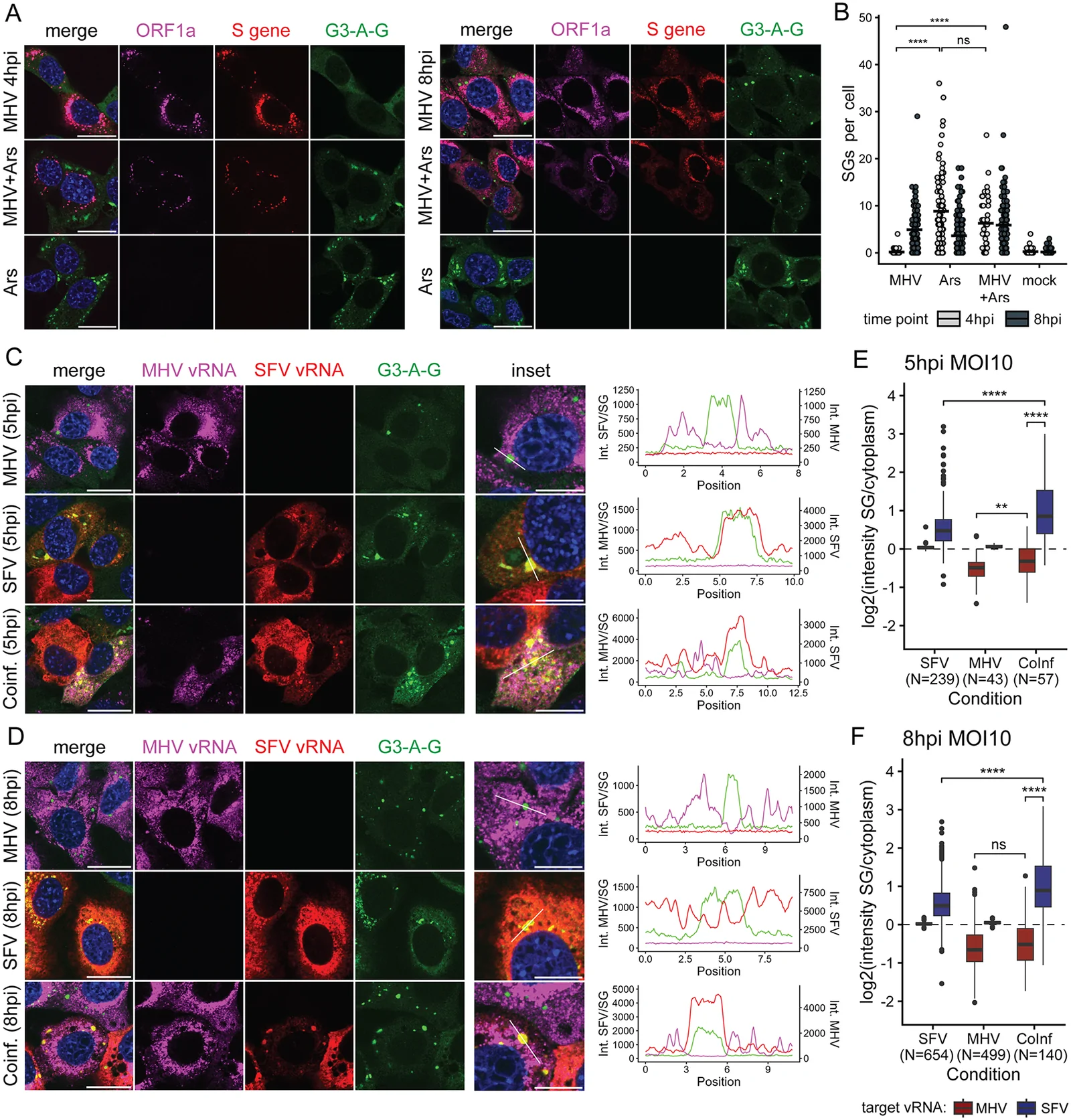
Formation of SGs during MHV infection induced by simultaneous sodium arsenite or SFV infection shows no accumulation of MHV vRNA. **(A)** Visualization of SGs and MHV viral RNA in 17Cl1:G3-A-G cells (green) infected with MHV (MOI 10) and treated with sodium arsenite (250 µM) 30 min before the indicated fixation time point at 4 hpi (left panel) or 8 hpi (right panel). Additional controls with each individual stressor are shown. Medium was supplemented with HR2 peptide to prevent syncytia formation. Cells were stained with smRNA-FISH probes targeting MHV ORF1a (magenta) and S gene (red). Nuclei were stained with DAPI. Scale bar: 20 μm. **(B)** SG number per cell for each condition and time point based on 6 fluorescence microscopy images per condition. Each dot represents a cell. For MHV and MHV + Ars only infected cells (exceeding defined vRNA intensity threshold) were included. Error bars indicate standard deviation. **(C/D)** Co-infection and single infection of 17Cl1:G3-A-G cells (green) with MHV and SFV at an MOI 10 and subsequent visualization of viral genomic RNA and SGs. Cells were fixed at (C) 5 hpi or (D) 8 hpi, and FISH staining was performed targeting MHV ORF1a (nsp1/2, magenta) and SFV ORF1 (nsP2, red). Intensity line plots display the measured signal intensity of the different probes and G3-A-G along the white line indicated in the inset. Scale bar: 20 μm / 10 μm (inset). **(E/F)** Log2 transformed mean fluorescence intensity of MHV or SFV probes in SGs normalized with intensity in the local cytoplasm around each SG. Number of analyzed SGs derived from five images for each condition is indicated in the condition label. The color indicates the targeted viral RNA. Statistical analysis with pairwise Wilcoxon signed-rank test and Benjamini-Hochberg correction. ****: *p* < 0.0001, **: p < 0.01, ns: nonsignificant. The data underlying this Figure can be found in S1 Data.

The absence of SGs early in MHV infection and the observed absence of MHV vRNA in SGs at later time points let us wonder whether SGs can be induced with another stressor during MHV infection. For alphaviruses like SFV it was previously shown that SGs cannot be reinduced by sodium arsenite after their disassembly by the viruses [15]. To test whether MHV can prevent SG formation early in infection, we treated MHV-infected 17Cl1:G3-A-G cells with sodium arsenite at 4 hpi and 8 hpi. As seen in Fig 10A, 10B, we observed the formation of SGs in infected and arsenite treated cells for both time points. In contrast, single MHV infection at 4 hpi only shows few cells with SGs (Fig 10B). These results demonstrate that MHV does not actively suppress SG formation at early time points of infection. Interestingly, the smRNA-FISH staining of MHV genomic and subgenomic RNA shows no accumulation of viral RNA in sodium arsenite induced SGs at 4 hpi as well as at 8 hpi similar to the previous observations for MHV-induced SGs (S17A, S17B Fig). Thus, the exclusion of MHV vRNA from SGs does not seem to be a property of the atypical SGs induced by MHV but rather a property of MHV itself reducing the accumulation of its RNA in SGs. To corroborate this hypothesis, we performed a coinfection experiment with MHV and SFV, as the latter showed an accumulation of its vRNA in SGs contrasting the observations for MHV. To differentiate both viruses, we performed smRNA-FISH targeting MHV ORF1a and SFV ORF1. As shown in Fig 10C, we observed a robust formation of SGs in double infected cells already at 5 hpi, in line with the early SG induction by SFV. Strikingly, in MHV and SFV co-infected cells, we found SFV vRNA to accumulate in the granules, while MHV vRNA was excluded at both investigated time points (Fig 10C, 10D). This general pattern of SFV vRNA enrichment and MHV vRNA exclusion can also be seen in the quantification of vRNA signal intensities in SGs compared to the surrounding cytoplasm especially for 8 hpi (Fig 10E, 10F). The strong virus-specific differences in vRNA accumulation in SGs occurring in the same cells highlight the different availability of SFV and MHV RNA for accumulation in SGs.

Combined, the results of the sodium arsenite treatment of MHV infected cells and the MHV-SFV co-infections suggest that MHV does not actively suppress SG formation during the early phase of infection. Furthermore, our investigations of vRNA presence in SGs induced by combining different stressors indicate that the underlying mechanism preventing accumulation of MHV vRNA in SGs is independent of the atypical characteristics observed for MHV-induced SGs and is rather an intrinsic property of how MHV protects its RNA.

## Discussion

A major focus of this study was based on characterizing the protein composition of the virus-induced SGs, as the presence or absence of specific proteins provides insight into possible roles of these condensates and associated host-virus interactions. For oxidative stress-induced SGs, which we use as a positive control, our results showed the expected proteome of canonical SGs. In line with previous findings by Markmiller and colleagues and others, we identified the major SG nucleator proteins, components of the preinitiation complex and a large variety of other proteins involved in RNA metabolism as abundant components of oxidative stress induced SGs verifying the labeling of the SG microenvironment [12,13]. Our screen further provides a set of novel SG protein candidates. Interestingly, we also detected several known SG associated proteins in the G3BP1 microenvironment under unstressed conditions as seen in the proximity labeling assay. A similar observation was made by Markmiller and colleagues and others suggesting that this could indicate a preexisting network of SG protein interactions in the unstressed cell, which is intensified upon stress [12,13]. Such stress-independent interactions between G3BP1 and other SG proteins could further explain that several known SG associated proteins did not show significant enrichment for arsenite treated or infected cells in our proximity labeling experiments, therefore leading to a potential underestimation of the proteins accumulated in SGs.

The granules formed during MHV infection show the accumulation of the major SG nucleator proteins including G3BP1, TIA1 and UBAP2L similar to canonical SGs. The presence of these SG markers was similarly observed in previous studies [20]. Several viruses, like Japanese encephalitis virus, have been shown to accumulate SG proteins in distinct viral aggregates to prevent SG formation [8]. However, this does not seem to be the case for MHV-induced granules as they are in an equilibrium with polysomes indicated by their disassembly with polysome stabilizing compounds. Furthermore, the increase in phosphorylated eIF2α simultaneously with granule appearance suggests that they are a result of the activated stress response during late MHV infection. Despite these canonical SG properties, MHV-induced SGs seem to be generally smaller, show a lower abundance of the majority of SG associated proteins including eIFs, and an overall reduced connection to SG biology compared to arsenite induced SGs. These observations suggest the formation of an atypical SG type during MHV infection. It is tempting to speculate that MHV induces a weaker stress response with less profound changes to the G3BP1 microenvironment and may even employ mechanisms to exclude or greatly reduce abundance of many canonical SG-associated proteins. In line with this, MHV infected cells show a lower level of phospho-eIF2α compared to arsenite stress and only low levels of dsRNA especially early in MHV infection.

In accordance with previous studies, we found that SGs formed during SFV infection resemble canonical SGs. They are formed upon the induction of the ISR by PKR activation and have a high abundance of SG nucleator proteins and components of the translation initiation machinery [15,29]. In our study, the ISR activation even exceeds the level of arsenite induced stress, demonstrating an efficient mounting of the stress response by infected cells early in SFV infection. In line with a strong stress induction by SFV, we observed high levels of dsRNA in SFV infected cells already after 3 hpi. Comparing the SG microenvironment between SFV and MHV, we found a similar enrichment of SG associated proteins for SFV as seen for oxidative stress, suggesting that the observed depletion of these factors for MHV-induced SGs is a virus-specific characteristic. Furthermore, SFV induces larger SGs than MHV, similar to arsenite treatment. Thus overall, SFV-induced SGs seem to be more similar to canonical SGs than the atypical SGs induced by MHV indicating that the latter are an MHV-specific SG type. The most striking difference thereby is a strong depletion of several translation initiation factors from SGs in MHV-infected cells, which seemingly exceed the reduced abundance of the other SG markers. Translation initiation factors are abundant in canonical SGs as most of them are part of the 48S preinitiation complex, a major component of SGs [37]. In contrast, other condensates like processing bodies (PBs) or the recently described paracrine granules formed in uninfected bystander cells during infection, are characterized by the absence of many eIFs [2,38]. However, our identified atypical SGs induced by MHV do seem to be distinct from both. PBs are enriched in proteins involved in mRNA decay like the decapping enzyme DCP1A, which we did not observe for MHV-induced SGs [2,13]. The paracrine granules on the other hand are characterized by the accumulation of large ribosomal proteins and their resistance to disassembly by cycloheximide, which are distinct from the atypical SGs described here [38]. Furthermore, as we used a high MOI for our experiments, the fraction of uninfected bystander cells is minimal.

The differences in eIF accumulation between SFV- and MHV-induced SGs is of great interest as many of these proteins are important host factors for viruses, which rely on the host translation machinery for viral protein production. As alpha- and coronaviruses possess 5′-cap structures on their genomic and subgenomic mRNAs, it is believed that they require the cap-dependent translation initiation machinery to produce viral proteins [39,40]. Interestingly, in the case of alphaviruses, the translation of subgenomic mRNA (sgmRNA) was found to be independent of eIF4F complex and eIF2, whereby the latter is mediated by a downstream stable hairpin structure in the coding region of the sgmRNA [29,41]. Nevertheless, there is evidence that sgmRNA translation of alphaviruses requires other factors including eIF3 [42]. Coronaviruses on the other hand were found to express nsp1 to profoundly manipulate host translation. For SARS-CoV-2, it was shown that this viral protein directly interacts with the ribosome causing translation inhibition of host transcripts, while promoting translation of viral RNAs [43,44]. Which eIFs are required for coronavirus translation is currently unclear. It was suggested that SGs might exert antiviral properties by sequestering eIFs to inhibit viral translation [45]. Based on our results, the eIFs seem to be either actively or passively prevented from accumulating in SGs during MHV infection, keeping them available for viral translation. The different eIF abundance in MHV and SFV induced SGs thus might indicate different ways how the viruses cope with the stress reaction and SG formation and ultimately a different role of these granules in both virus infections. Currently, we do not know the underlying mechanism of eIF reduction in MHV-induced atypical SGs and besides an active high jacking by viral proteins, several other factors could contribute. As discussed above, the weaker stress response observed for MHV could also lead to a weaker accumulation of many SG proteins, which is in line with the smaller size of the atypical SGs. On the other side, the efficient translation of viral RNA at later time points might occupy a large fraction of eIFs preventing their accumulation in SGs. This could also lead to smaller granules and would further explain why the major SG nucleator still seem to accumulate in comparable levels for canonical and atypical SGs. Another factor could be the maturation stage of SGs as a previous study showed differences in the SG proteome depending on their maturation stage [46]. As we only compare the peak time point for each virus, we cannot exclude that some differences might arise from different stages of SG maturation.

Besides the distinct protein composition, our study further revealed virus-specific differences in the accumulation of vRNA in MHV- and SFV-induced SGs. Especially, the exclusion of MHV vRNA from SGs contrasting the observed accumulation for SFV vRNA raises the question on underlying mechanisms. As MHV vRNA is also excluded from SGs induced by sodium arsenite and SFV, it does not seem to be a specific characteristic of MHV-induced atypical SGs. A more likely explanation could be differences in the availability of vRNA in the cytoplasm between SFV and MHV. Alphaviruses like SFV replicate in single-membrane spherules with an opening at one end known to “leak” vRNA into the cytoplasm and activating host sensors like PKR early in infection [15]. In contrast, MHV and other CoVs replicate in more complex double membrane vesicles (DMVs) with viral protein pores as only connections to the cytoplasm [47,48]. Furthermore, as seen with our dsRNA measurements, MHV seems to produce lower levels of dsRNA and possesses mechanisms to regulate vRNA amounts. Another mechanism of vRNA exclusion could be the interaction with viral proteins like the vRNA-binding N, or the efficient translation potentially mediated by nsp1 preventing the recruitment into SGs. Further investigations are needed to assess which mechanism is driving MHV vRNA exclusion from SGs. Nevertheless, the striking difference in vRNA accumulation between SFV and MHV is of special interest because some suggested antiviral mechanisms of SGs are associated with the recruitment of vRNA to these condensates. By bringing together vRNA and sensors like RIG-I and PKR, SGs could serve as signaling platforms regulating the antiviral response [5,7]. However, in contrast to several studies reporting an accumulation of RIG-I, PKR and OAS in antiviral SGs during Sendai virus and influenza A ΔNS1 virus infections, our proteomics screen showed no specific enrichment of these proteins neither for the atypical SGs induced by MHV, nor for the canonical SGs induced by SFV [5–7]. Importantly, the used 17Cl1 cell line has long been used for MHV infection studies, also because it is fully IFN competent and contains functional RIG-I, PKR and OAS. Thus, the absence of these innate immune sensors in SGs in our study suggest that their abundance in SGs likely differs between viruses, even from the same family, and does not seem to be a general feature of virus-induced SGs. Another suggested antiviral mechanism is the sequestration of vRNA as a recent study found that the condensation of vRNA in SGs during SARS-CoV-2 infection limits viral growth [10]. In the context of these findings, it is striking that MHV efficiently excludes gRNA and sgRNA from virus-induced SGs, while SFV shows a strong accumulation of gRNA in these granules. Because the observed exclusion of vRNA for MHV could counteract the proposed antiviral mechanisms of SGs, our findings suggest different roles of the granules during MHV and SFV infections.

Besides the differences in composition, the varying role of MHV and SFV induced SGs during both infections is further supported by their formation kinetics observed in our live-cell imaging. SFV triggers the SG formation early in infection as observed previously [9]. This formation is preceded by a strong activation of the cellular stress response seen by elevated phospho-eIF2α levels already before SG assembly. In line with an early ISR activation, we observed high levels of dsRNA already 3 h after SFV infection. With progressing infection SFV actively disassembles the granules likely mediated by the nsP3 directly interacting with G3BP1 [9,34]. This disassembly of SGs seems to occur prior to the exponential growth phase of SFV suggesting that SFV might have to disassemble SGs to facilitate production of virus progeny, which is in line with a previous study reporting a correlation between SG disassembly and higher levels of viral RNA [29]. In contrast, the SGs during MHV infection only appear at a late stage of infection with increasing production of new virus particles. At early stages of infection, the virus seems to prevent the induction of the stress response and subsequent SG formation. This prevention of stress induction seems to be mediated by the efficient hiding of MHV from cellular sensors rather than an active inhibition. Firstly, we found that SGs can still be induced by SFV co-infection or sodium arsenite treatment at early time points of MHV infection contradicting an active interference with the stress pathway. Secondly, coronaviruses are well known to hide their dsRNA in DMVs and further possess an arsenal of nsps, like the endonuclease nsp15, to prevent its detection by host sensors [18]. This is further supported by our observation that MHV only produces low levels of dsRNA, which are increased when the nsp15 endonuclease activity is impaired. Although not inducing the granules earlier, the lack of nsp15 activity and the increased dsRNA levels seem to increase the strength and duration of the SG response supporting that dsRNA-mediated activation of ISR might be a main driver of the SG formation during MHV infection. When the granules are finally formed, the virus has already established the infection and completed most of its life cycle. Thus, MHV does not only seems to induce a weaker stress response compared to SFV, but it also does so only at a late stage where a potential antiviral effect on viral growth is limited. The striking differences in the timing of SG appearance in relation to virus growth between MHV and SFV indicate different replication strategies of these viruses. While SFV seems to quickly overwhelm the cell and employ countermeasures to cope with activated host responses, like the disassembly of SGs or the independence form eIF2, MHV seems to remain undetected for a longer period while establishing the infection. The weak stress stimulus combined with the late time point of SG formation during MHV infection might give rise to the altered proteome of the atypical SGs characterized in this work.

**Fig 11:**
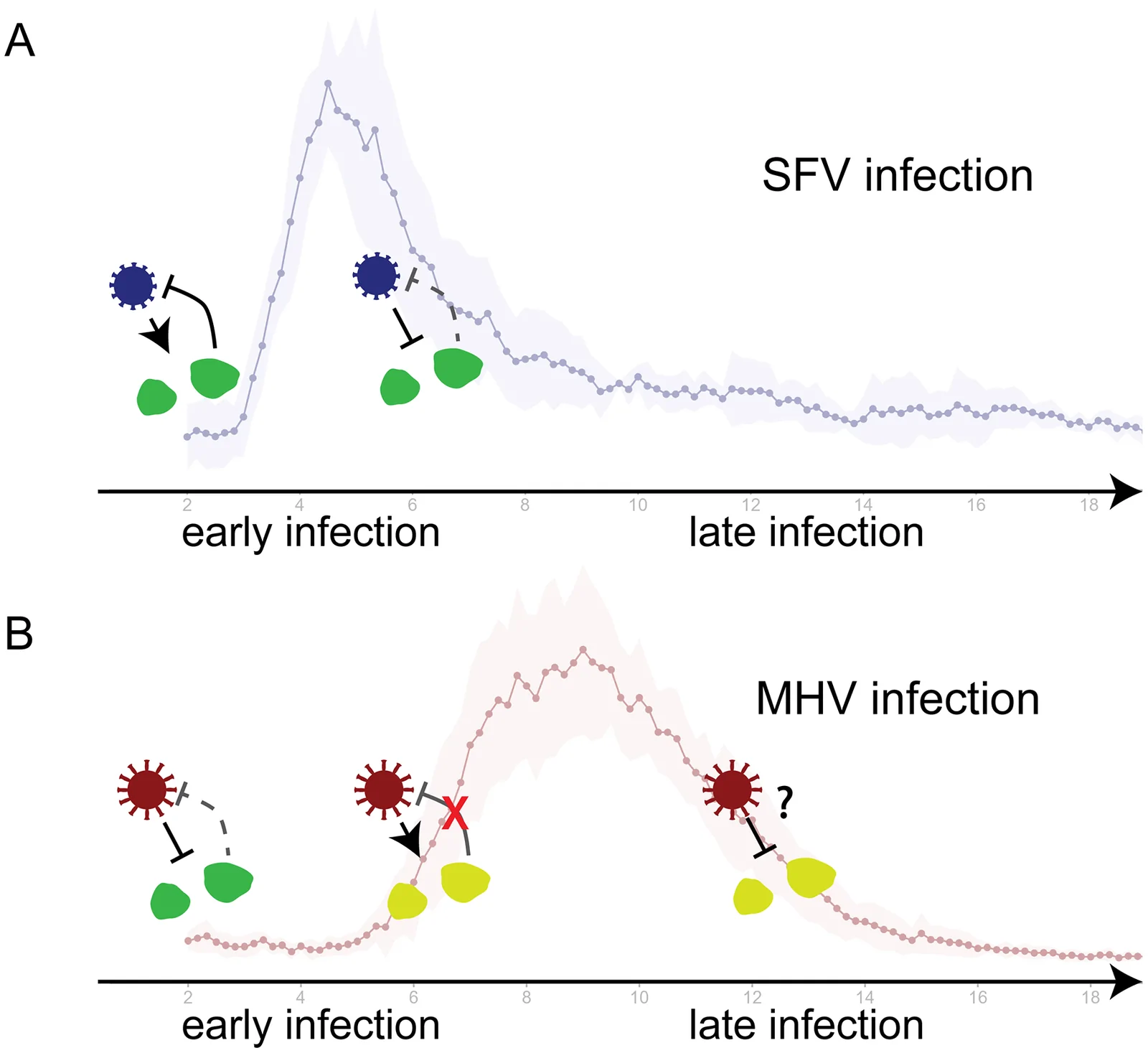
Proposed model of host-virus interaction in the context of SGs during SFV and MHV infections in cells. **(A)** SFV seems to induce canonical SGs early in infection characterized by the presence of well-known SG marker proteins like eukaryotic translation initiation factors. The sequestration of especially the eIFs, which are required by the virus for efficient viral translation, as well as the accumulation of SFV genomic RNA in these condensates could have negative effects on viral growth. Thus, the virus dissolves the SGs with progressing infection. **(B)** In contrast, MHV seems to prevent induction of canonical SGs early in infection. Only with the onset of exponential release of new virus particles, accumulations positive for the major SG markers G3BP1, TIA1, and UBAP2L are formed in the cytoplasm. A reduced abundance of many known SG proteins including eIFs compared to canonical SGs suggests a type of atypical SGs. Together with the efficient exclusion of MHV viral RNA, this gives rise to the apparent tolerance of these condensates by MHV later in infection.

Altogether, our study revealed substantial virus-specific differences for the investigated SGs contradicting the formation of a general “antiviral SG”. While SFV seems to induce canonical SGs, our investigation reveals the formation of distinct, atypical SGs during MHV infection characterized by the reduced abundance of some canonical SG components like eIFs, the absence of vRNA in the granules, and a smaller size. These differences in SG characteristics further support different roles of these granules during SFV and MHV infection strongly shaped by different virus-host interactions. Our current understanding (Fig 11) is that SFV induces the formation of canonical SGs early in infection likely triggered by sensing of vRNA via the ISR. These SGs accumulate canonical SG proteins including eIFs as well as viral RNA, which could limit viral growth by reducing the availability of these components for SFV. To efficiently produce new virus particles, SFV actively dissolves the granules as infection progresses and prevents their reformation [9]. In contrast, MHV prevents ISR activation and SG formation during the early phase of infection by efficiently hiding its vRNA. Only at a later stage is the stress response activated and G3BP1 positive granules are formed. However, these atypical SGs show a reduced abundance of many SG proteins and an altered composition distinct from canonical SGs. The reduced accumulation of eIFs and the exclusion of vRNA from MHV-induced granules likely counteracts antiviral functions mediated by sequestering important host-factors and vRNA. The altered composition in combination with the late induction time point allows the virus to tolerate the presence of these granules until their disassembly during the collapse of cellular homeostasis. Collectively, our work revealed a remarkable and surprising plasticity of SG formation and composition that is to a large extent dependent on the type of virus infection. In particular, the greatly reduced abundance of canonical SG proteins in MHV-induced SGs and the exclusion of MHV RNA from SGs raise the question, if these granules exert any antiviral function. For example, if MHV, and possibly other coronaviruses, employ mechanisms (i) to prevent SG formation early during infection, (ii) to modulate the SG proteome, and (iii) to exclude viral RNA from SGs. Our work also provides an impetus for future studies on SG biology in general, and the role(s) of SGs during infection with coronaviruses, alphaviruses, and beyond. As with many other cellular processes, basic principles of cell biology can be deciphered by using virus infection models, and as our study suggests, basic determinants of SG formation and SG composition are still unknown.

## Materials and methods

### Cell culture

Murine 17 Clone 1 fibroblasts (17Cl1, kindly provided by S.G. Sawicki) and murine 929 fibroblasts (L929, Sigma) were cultured on minimum essential medium (MEM, Gibco) supplemented with 10% (v/v) FBS, 1x Penicillin-Streptomycin (seraglob) and 1% (w/v) nonessential amino acids (Gibco) (MEM+/+/+). Human Hek293T cells were cultured in Dulbecco’s Modified Eagle Medium-GlutaMAX (DMEM, Gibco) supplemented with 1 mM sodium pyruvate, 10% (v/v) FBS, 100 μg/ml streptomycin, 100 IU/ml penicillin (Gibco) (DMEM+/+). Cells were grown in a humidified incubator at 37 °C and 5% CO_2_.

### Viruses

Recombinant MHV A59 strain and MHV-nsp15 (H277A) mutant strain were previously generated using a reverse genetic system based on vaccinia virus [18,49]. A plasmid encoding the genome of SFV was kindly provided by Oliver Mühlemann. The sequence was modified with standard molecular techniques to represent the wildtype SFV strain 4. SFV mRNA was in vitro transcribed from the linearized plasmid and capped using the RiboMAX Large Scale RNA Production System for the SP6 promoter (Promega). To rescue infectious virus, baby hamster kidney cells (BHK21, propagated in MEM+/+/+) were electroporated with SFV mRNA and cocultured with susceptible 17Cl1 cells. The resulting passage 0 was used to produce progeny passage stocks on 17Cl1 cells for all infection experiments. Viral titers were estimated by plaque assay on 17Cl1 or L929 cells. To summarize, cells were grown in 24-well plates and infected with a 10-fold serial dilution of virus stock. The infection medium was replaced with overlay medium (DMEM supplemented with 10% FBS, 1x P/S, 1x NEAA, and 1% methylcellulose) 2 hpi. After 1 day, the cells were fixed with 4% formalin for 15 min and stained with crystal violet for 10 min. Titers were calculated based on the counted plaques.

### Generation of stable cell lines 17Clone1: G3BP1-APEX2-eGFP cells

First, the In-Fusion HD cloning kit (Takara) was used to integrate the coding sequence of the fusion protein mG3BP1-APEX2-GFP under the control of an HSV-TK promotor into an adapted pSMWUP lentiviral vector (CellBioLabs). Next, lentiviruses were produced in Hek293T cells by co-transfecting the cells with modified pSMWUP(TK:mG3BP1-APEX2-eGFP), pMD2G (addgene 12,259) and CMVR8.74 (addgene 22,036) using X-tremeGene HP DNA Transfection Reagent (Sigma–Aldrich). Produced lentiviruses were used to infect 17Cl1 cells, which were subsequently treated with puromycin (2 μg/ml) to select for transduced cells. Surviving cells were sorted based on GFP signal using flow cytometry. 17Cl1:G3BP1-APEX2-eGFP (17Cl1:G3-A-G) cells were further propagated in MEM+/+/+ maintaining the puromycin selection at 1 μg/ml. Puromycin was omitted for all experiments.

### Viral replication kinetics

17Cl1 cells were seeded in 24-well plates to reach 80%–100% confluency the following day. Cells were infected with MHV or SFV at an MOI of 10 or mock infected without virus. Infection was synchronized by incubation of cells on ice for 1 h, followed by incubation at 37 °C for 1.5 h. Cells were washed 3x with PBS and fresh MEM+/+/+ was added. Supernatant was collected at time points indicated in Fig 4C and virus titers were estimated by TCID50 assay. In short, 17Cl1 cells were infected with a 10-fold serial dilution of the supernatants (6 columns of 96-well plate pr sample) and incubated for 3 days at 37 °C. The cells were fixed and stained with crystal violet. Based on the highest dilutions displaying dead cells, TCID50 was calculated using the Spearman and Kärber algorithm [50]. The kinetics time course was carried out in three independent replicates. The mean titers and corresponding standard deviations were calculated and plotted in RStudio using ggplot2 and dplyr packages [51].

### Indirect immunofluorescence

Glass coverslips were coated with poly-D-lysine solution for 1 h and washed 3x with PBS. Recombinant 17Cl1:G3-A-G cells were seeded on coated glass coverslips in a 12-well plate at a density of 2.25 x 10^5^ cells/well. The following day, cells were infected with MHV or SFV at an MOI of 10 and infection was synchronized as described above. Cells were washed 3x with PBS and fresh MEM+/+//N was added. Medium for MHV samples was supplemented with 1 μM HR2 peptide. Samples for sodium arsenite treatment were incubated with MEM+/+/N supplemented with 250 μM NaAsO_2_ (Sigma) 30 min before fixation. For cycloheximide treatments, the medium was exchanged 1.5 h before fixation with medium supplemented with 50 μM cycloheximide (Sigma). For the sodium arsenite treatment, fresh medium supplemented with sodium arsenite and cycloheximide was added 1 h after the start of cycloheximide treatment and 30 min before fixation. At the indicated time point, cells were washed 2 times with PBS and fixed with 4% formalin for 20 min. Next, cells were washed and stored in PBS at 4 °C until staining.

For the staining procedure, the cells were permeabilized first by incubation in 0.1% Triton X-100 (in PBS) for 5 min at room temperature, followed by 20 min incubation in confocal buffer (CB) consisting of PBS supplemented with 50 mM NH_4_Cl, 0.1% saponin, and 2% BSA. Afterwards, primary antibodies were diluted in CB as indicated in Table 1. Glass coverslips were incubated in primary antibody solution for 2 h at room temperature. Excess antibody solution was removed by washing the cells 3x 5 min with CB, before incubating the cells with secondary antibody solution for 1 h at room temperature. Afterwards, samples were washed firstly 3x with CB for 5 min, followed by one wash with PBS and a quick rinse with water. Lastly, the cells were mounted on ProLong Diamond mounting medium containing DAPI (Lifetechnologies).

**Table 1:** Antibodies/reagents for indirect immunofluorescence staining and western blot analysis with used dilution factor and source.

Antibody or reagent	Source	Identifier	Used dilution
anti-dsRNA (mouse mAb)	SCICONS	J2	1:200/1:1000
anti-G3BP1 (rabbit pAb)	Sigma	G6046	1:200
anti-G3BP1 (mouse mAb)	Proteintech	66486-1-Ig	1:200
anti-TIA1 (rabbit pAb)	Proteintech	12133-2-AP	1:200
anti-UBAP2L (rabbit mAb)	Cell signaling	40,199	1:200
anti-eIF3a (rabbit mAb)	Cell signaling	3,411	1:400
anti-eIF3E (rabbit pAb)	Proteintech	10899-1-AP	1:50
anti-eIF3L (mouse mAb)	Proteintech	67719-1-Ig	1:400
anti-eIF4A3 (rabbit mAb)	abcam	ab180573	1:250
anti-eIF4E (rabbit mAb)	abcam	ab33766	1:250
anti-eIF4G1 (rabbit pAb)	Cell signaling	2,858	1:200
anti-eIF4H (D85F2) XP (rabbit mAb)	Cell signaling	3,469	1:400
anti-PABP (rabbit pAb)	abcam	ab21060	1:400
anti-FUS/TLS (rabbit mAb)	Cell signaling	67,840	1:800
anti-TAF15 (8TA-2B10) (mouse mAb)	Invitrogen	MA3–078	1:250
anti-PTBP1 (rabbit pAb)	Proteintech	12582-1-AP	1:100
anti-eIF2α (rabbit mAb)	Santa Cruz Biotechnology	sc-133132	1:500
anti-phospho-eIF2α (S51) (mouse mAb)	abcam	ab32157	1:5′000
anti-α-tubulin	Proteintech	66031-1-lg	1:20′000
Streptavidin Alexa 594 conjugate	Molecular Probes	S-32356	1:400
Avidin, NeutrAvdin HRP conjugate	Invitrogen	A2664	1:2′500
donkey anti-rabbit Alexa 488	Jackson ImmunoResearch	711-545-152	1:400
donkey anti-rabbit Alexa 594	Jackson ImmunoResearch	711-585-152	1:400
donkey anti-mouse Alexa 647	Jackson ImmunoResearch	715-605-150	1:400
Peroxidase-conjugated Donkey anti-Mouse	Jackson ImmunoResearch	715-035-151	1:5′000
Peroxidase-conjugated Donkey anti-Rabbit	Jackson ImmunoResearch	711-035-152	1:5′000

Image acquisition by fluorescence microscopy was done on a DeltaVision Elite High-Resolution widefield imaging system (General Electric) or a Nikon Ti-2 Cicero spinning disk confocal microscope (Nikon) using 60x or 100x oil immersion objectives. For each imaged point a Z-stack with a Z resolution of 0.2 μm was acquired. Images acquired with the DeltaVision widefield system were additionally deconvolved with the in-built software softWoRx. The post-processing including a maximum intensity Z projection was done with the software Fiji and figures were assembled using the FigureJ plug-in [52,53]. Quantification of signal intensity in cytoplasma was done by segmenting cytoplasma based on G3-A-G channel using Ilastik [54]. Intensity of second channel in segmented cytoplasma was measured in Fiji and the average intensity on a per image base (normalized with area) was plotted in RStudio. One-way ANOVA and subsequent pairwise *t* test was performed to determine significance.

### Live-cell imaging and image analysis

17Cl1:G3-A-G cells were seeded in 8-chambered glass bottom slides (ibidi, 80,807) and infected with SFV or MHV at an MOI 10 or MHV-nsp15 (H277A). Infection was synchronized by first incubating the slide on ice for 1 h, followed by incubation at 37 °C for 1.5 h. Infection medium was exchanged with fresh MEM+/+/+ supplemented with 1:40 HEPES (gibco). Medium for MHV infected samples additionally contained 1 μM HR2 peptide to prevent syncytia formation. Cells were imaged on a DeltaVision elite system fluorescence microscope equipped with a 40x oil immersion objective, an on-stage incubator chamber at 37 °C, and 5% CO_2_ supply. Multiple points per condition were imaged from 2 hpi up to 24 hpi with an imaging interval of 10 min.

Data analysis was done with an image post-processing and quantification workflow based on Fiji (ImageJ 2.16.2) and Ilastik (V1.4.1). In short, an initial processing was done in Fiji including a maximum intensity Z projection. Next, Ilastik was used to train a pixel classification model on a subset of imaging data to differentiate between SG and background. The trained model was subsequently used to generate pixel prediction masks for all image series, which were further used to segment and count SGs in each image with an automated script in Fiji. The cell number for each image series was derived by manually counting the cells in selected frames throughout the imaging period. The number of SGs for each image was normalized with the corresponding cell number. The mean value over multiple image series was calculated for each condition and plotted using RStudio and the packages tidyverse and ggplot2 [55].

### SILAC labeling with isotopically labeled amino acids

All SILAC experiments were carried out with special medium either containing heavy isotope labeled arginine/lysine or normal isotope labeled arginine/lysine. MEM without L-arginine and L-lysine (MEM for SILAC, Thermo Fisher) was supplemented with 10% (v/v) FBS, 100 μg/ml streptomycin, 100 IU/ml penicillin (Gibco), 1x NEAA (Gibco), and 0.25 mM L-proline:HCl (Thermo Fisher). The “light medium” was additionally supplemented with 0.6 mM normal isotope labeled L-arginine:HCl (Thermo Fisher) and 0.4 mM normal isotope labeled L-lysine:2 HCl (Thermo Fisher). The “heavy” medium was additionally supplemented with heavy isotope labeled L-arginine:HCl (^13^C_6_, ^15^N_4_; Cambridge Isotope laboratories) and heavy isotope labeled L-lysine:2HCl (^13^C_6_, ^15^N_2_; Cambridge Isotope laboratories).

Two batches of recombinant 17Cl1:G3-A-G cells were propagated on either heavy or light medium for a minimum of three weeks, and incorporation of isotopically labeled amino acids was verified by mass spectrometry.

### APEX2-mediated proximity biotinylation

Recombinant 17Cl1:G3-A-G cells propagated on either heavy or light SILAC medium were seeded in 10 cm dishes 2 days prior to infection. Dishes for 2nd and 3rd SILAC experiments further contained a glass coverslip for visual verification of proximity labeling by IF (staining procedure described above). Cells were infected with either MHV-A59 at MOI 5 (1st experiment) or 10 (2nd experiment), or SFV at MOI 10. Infection media were additionally supplemented with 25 mM HEPES (Gibco). Samples for arsenite treatment or mock infection were incubated in fresh SILAC medium. The infection was synchronized by incubation of the dishes on ice for 1 h, followed by 1.5 h at 37 °C. Infection medium was aspirated, cells were washed 3x with PBS, and fresh prewarmed SILAC medium was added. 30 min prior to the respective labeling time point medium was replaced by fresh prewarmed SILAC medium supplemented with 0.5 mM (1st experiment) or 2.5 mM (2nd/3rd experiment) BP. Samples for negative labeling control received medium without BP. APEX2-mediated proximity labeling was carried out by direct addition of hydrogen peroxide to the medium at a final concentration of ~1 mM. After incubation of 60 s at room temperature the medium was removed, and cells were washed 3x with quencher solution consisting of PBS supplemented with Trolox (5 mM, Sigma), sodium ascorbate (10 mM, Sigma), and sodium azide (10 mM, Sigma). The quencher solution was freshly prepared for each labeling time point. Cells were lysed in 1 ml lysis buffer (50 mM Tris-HCl pH 7.4, 500 mM NaCl, 0.2% SDS, 1x complete Mini Protease inhibitor cocktail (Roche), 5 mM Trolox, 10 mM sodium ascorbate, and 10 mM sodium azide in H_2_O), scraped off and collected in a tube. Triton-X100 was further added to the lysates to a final concentration of 2%. Next, the lysates were homogenized using a sonicator or MagNA lyser tubes (Roche), and cleared by centrifugation at 12′000 rpm for 10 min at 4 °C. The protein concentration of each lysate was assessed by 660 nm protein assay (Pierce), and light and corresponding heavy samples were mixed in a 1:1 protein ratio, 500 μg of each. For the affinity purification, 100 μl (1st experiment) or 500 μl (2nd/3rd experiment) streptavidin coated magnetic beads (Pierce Streptavidin Magnetic Beads, Thermo Fisher) for each mixed lysate were washed 2x with lysis buffer. The mixed lysates were then incubated on the beads overnight at 4 °C on a rotator. The next day, the supernatants were collected and stored for further analysis. A stringent washing procedure of the beads was performed consisting of two washes with buffer 1 (2% SDS in H_2_O), one wash with wash buffer 2 (2M Urea, 10 mM Tris-HCl pH 7.4), two washes with wash buffer 3 (0.1% (w/v) deoxycholate, 1% (vol/vol) Triton X-100, 1mM EDTA, 500 mM NaCl, and 50 mM HEPES pH 7.5 in H_2_O), two washes with wash buffer 4 (0.5% (w/v) deoxycholate, 0.5% (vol/vol) NP-40, 1 mM EDTA, 250 mM LiCl, 10 mM Tris-HCl pH 7.4 in H_2_O) and a final wash with 50 mM Tris-HCl pH 7.4. Each wash included 8 min incubation on a rotator, followed by 1 min incubation on a magnetic rack. To elute the proximity labeled proteins, the beads were incubated in 50 μl elution buffer (75 mM Tris pH 6.8, 12% glycerol, 3% SDS, 6% β-mercaptoethanol, 10 mM biotin) at 95 °C for 10 min on a shaking heat block (600 rpm). The eluates were stored at −80 °C until mass spectrometric analysis. The proximity labeling and affinity purification were verified by western blot analysis of lysates, eluates and flowthroughs using horse radish peroxidase (HRP) conjugated streptavidin (Dako) or NeutrAvidin (Invitrogen) to detect biotinylated proteins. For the 2nd and 3rd experiment the subcellular localization of biotinylated proteins was further assessed by immunofluorescence microscopy using AlexaFluor 594 conjugated streptavidin (Invitrogen) (procedure described above).

### Mass spectrometry analysis

After affinity purification, eluates were fractionated according to protein size by SDS-PAGE and subsequent cutting of gel into 1 mm^3^ cubes. The samples were digested in-gel and subjected to liquid chromatography tandem mass spectrometry (LC-MS/MS). In short, the gel cubes were reduced, alkylated, and digested by trypsin as described previously [56]. After loading, peptides were eluted in back flush mode onto the analytical self-packed nano-column (Acquity CSH C18, 1.7 µm, 100 A, 0.075 mm i.d. x 200 mm length, Waters, Baden, Switzerland) using an acetonitrile gradient of 5%–40% solvent B (0.1% Formic Acid in water/acetonitrile 4,9:95) in 60 min at a flow rate of 350 nL/min. The column effluent was directly coupled to a Fusion LUMOS mass spectrometer (Thermo Fischer, Bremen; Germany) via a nano-spray ESI source.

Data acquisition was made in data dependent mode with precursor ion scans recorded in the orbitrap with resolution of 120′000 (at m/z = 250) parallel to top speed fragment spectra of the most intense precursor ions in the Linear trap for a cycle time of 3 s maximum.

Mass spectrometry data were analyzed as previously described [57]. Briefly, proteins were identified by MaxQuant (version 2.5.1.0) against mouse protein database (SwissProt, release April 2025_01), MHV strain A59 database (UniprotKB, release March 2023) and a curated list of SFV proteins [58]. The initial precursor mass tolerance was set to 10 ppm, and that of the fragment peaks to 0.4 Da. Enzyme specificity was set to strict trypsin, and a maximum of three missed cleavages were allowed. Carbamidomethylation on cysteine was set as a fixed modification, while methionine oxidation, and protein N-terminal acetylation were set as variable modifications.

After removal of common contaminants and inversing reversed ratios, 1-sample *t* tests were performed on the MaxQuant normalized ratios, provided the ratios were measured in all replicates. Adjusted *p*-values for multiple testing were corrected by the R fdrtool package [59].

Protein intensities were furthermore calculated as the median of the log_2_-transform of the intensities of all peptides allocated to the protein, using the heavy and light channels as separate samples, as previously described [12]. Normalization was performed by the global median method, whereby the viral proteins as well as any previously known SG proteins (curated list of tire 1 SG proteins based on RNA Granule Database, December 2025 [22]) were excluded from the calculation of the normalization coefficients. Missing protein intensities were imputed in the following manner, provided there was at least two identifications in at least one group: if there was at most two identifications in the group of replicates, the missing values were drawn from a Gaussian distribution of width 0.3 centered at the sample distribution mean minus 2.5x the sample standard deviation, otherwise the Maximum Likelihood Estimation (MLE) method was used [60]. Log_2_ fold changes (Log2FC) between given conditions were calculated by using mean Log2 of the respective conditions. Differential expression tests were performed by applying an Empirical Bayes test [61], and the FDR-adjusted *p*-values (Benjamini and Hochberg multiple test correction [62]) were calculated. Significantly enriched or depleted proteins (adj. *p*-value ≤ 0.05 for large Log2FC, and |Log2(FC)| > 2 for individual comparisons or> log2(2.5) for SILAC based comparisons) were determined between different conditions. Data visualization was performed in R: Gene Ontology analysis was performed using the clusterProfiler (V4.16.0) and org.Mm.e.g.,db (V3.21.0) packages [63]. Redundant GO terms for each condition were reduced based on semantic similarity (similarity threshold: 0.7, method: relevance) using rrvgo package (V1.20.0) [64]. Venn diagrams were generated with VennDiagram package (V1.7.3) [65]. Further data analysis and visualization were performed using ggplot2 and tidyverse (V2.0.0) packages.

### Western blot analysis

17Cl1:G3-A-G cells were grown in 12-well plates and infected with MHV or SFV at an MOI of 10, or mock infected as described above. At the indicated time points, medium was removed, cells were washed once with PBS and lysed in lysis buffer (M-PER Mammalian Protein Extraction Reagent, Thermo Scientific) supplemented with cOmplete mini protease inhibitor (Roche) and phosphatase inhibitor (Thermo Scientific). Protein concentration was determined using Pierce BCA Protein assay (Thermo Scientific) and 15 ng total protein was mixed with Lämmli buffer (supplemented with β-mercaptoethanol) and incubated at 95 °C for 5 min. Samples were run on an SDS-PAGE (4%–20% gradient, SurePAGE GenScript) and blotted onto nitrocellulose membranes. Membranes were blocked in TBS + 0.1% Tween-20 (TBS-T) supplemented with 5% milk powder for 1 h at room temperature. Staining with primary (anti-eIF2α, anti-phospho-eIF2α, α-tubulin) and secondary antibodies was done in TBS-T + 0.5% milk over night at 4 °C or for 1 h at room temperature, respectively. Used antibody dilutions are found in Table 1. Membranes were washed 3x with TBS for 5 min. Membranes were developed with WesternBright ECL HRP substrate (Advansta) and imaged on a FusionFX. Band intensities were measured in Fiji (ImageJ 2.16.2) and plotted in RStudio. In short, intensity for eIF2α and p-eIF2α bands was loading corrected with the corresponding loading control signal. The p-eIF2α/eIF2α ratio was calculated and normalized with the Arsenite sample. Statistical analysis was done by performing a pairwise Wilcoxon signed-rank test and Benjamini-Hochberg FDR correction in RStudio (V 2026.01.0+392) comparing each stress condition with the corresponding mock control using the tidyverse (V2.0.0) and ggpubr (V0.6.2) packages.

### smRNA FISH

17Cl1:G3-A-G cells were seeded on glass coverslips and infected with MHV or SFV at an MOI of 0.1 or 10 as described above. The smFISH protocol used to visualize viral mRNAs in this study was executed as previously detailed by Dave and colleagues (2023) [66]. Cells were fixed at the specified time points post-infection using 4% paraformaldehyde in PBS for 20 min. The cells were then permeabilized with 0.5% Triton X-100 solution for 10 min and pre-blocked for 15 min in wash buffer consisting of 2xSSC (Invitrogen) and 10% v/v formamide (Ambion). For simultaneous IF staining, preblocking was done with wash buffer supplemented with 3% BSA for 30 min. Hybridization with fluorescently labeled oligos was then carried out using a hybridization buffer containing 150 nM smFISH probes specific to MHV genomic RNA (nsp1–2 genes), S-gene, SFV genomic RNA (nsP2 gene), or mmATF4 mRNA, along with 2xSSC, 10% v/v formamide, and 10% w/v dextran sulfate (Sigma), for 4 h at 37 °C. Hybridization buffer was additionally supplemented with primary antibody (anti-TIA1, 1:200) for combined IF staining. Following hybridization, cells were washed twice with wash buffer for 30 min each and subsequently 3x with PBS. For combined IF staining, coverslips were incubated on drops of secondary antibody solution (anti-rabbit Alexa 488, 1:400 in PBS) for 1 h at 4 °C and subsequently washed 3x with PBS. Coverslips were mounted on slides with ProLong Gold antifade reagent containing DAPI (Molecular Probes). The 20-nucleotide oligonucleotides targeting various viral RNAs (S5 Table) were designed using the Stellaris probe designer and conjugated to either Atto-565 or Atto-633, as described in the protocol by Gasper and colleagues (2017) [67].

Images were acquired using a Zeiss AxioObserver7 inverted microscope equipped with a Yokogawa CSU W1-T2 spinning disk confocal scanning unit, a Plan-APOCHROMAT 100x 1.4 NA oil objective, an sCMOS camera, and an X-Cite 120 EXFO metal halide light source. Imaging was performed with 21 z-stacks at 0.24 µm intervals.

Microscopy images were analyzed for colocalization in Fiji (ImageJ 2.16.2). The images were cropped to include a single cell per image and intensity of signal across multiple channels was measured using the plot profile function in ImageJ across the ROI (straight line across the object of interest). The plot profiles across channels were then plotted in python (seaborn) or RStudio (V 2026.01.0+392).

### Image quantification and statistical analysis

The quantification of SG size, number, and signal intensities of stained target proteins and vRNAs was done using an image analysis workflow based on Fiji (ImageJ 2.16.2)/Ilastik (V1.4.1) and Python (cellpose). In short, pixel prediction masks for SGs were produced using an Ilastik autocontext pixel prediction model trained with a subset of data. The prediction maps were then used for segmentation of SGs in Python and subsequent measurement of area and mean intensity of channels of interest. Individual cells and nuclei were segmented in Python (V3.9.25) using cellpose (V4.0.8) [68]. SG number for each cell and mean intensity of channels of interest in the cytoplasm (cell mask excluding nucleus and SGs) were measured. For a local cytoplasma sample for each SG used for the quantification of vRNA intensities, a doughnut-shaped mask was generated around each SG (excluding other SGs, 3px margin between SG and mask). The mean intensity of the target channel in each SG or the mean intensity in each SG over the mean intensity in the corresponding local cytoplasm was plotted in RStudio. Single Z sections were used for intensity measurements. A maximum Z projection was used for analysis of SG number per cell. For the analysis of vRNA signals in the coinfection experiment, only SGs of infected (single infections) and double-infected (coinfection) cells were included based on vRNA intensity thresholds in the cytoplasm (MHV: 199.43/SFV: 531.34).

Significance between different conditions, treatments or time points based on groups of SGs, cells, and/or experiments (as indicated in the corresponding figure legend) was tested by performing Kruskal–Wallis test followed by post-hoc pairwise Wilcoxon signed-rank test including *p* value adjustment by Benjamini-Hochberg FDR correction, in RStudio (V 2026.01.0+392) using the tidyverse (V2.0.0) and ggpubr (V0.6.2) packages.

### Flowcytometry

Single-cell suspensions were prepared by infecting 17Cl1 wildtype cells with SFV or MHV at MOI 10 or with MHV-nsp15 (H277A) at MOI 5 as described above. Cells were detached with enzyme-free dissociation reagent and carefully scraped off. Cells (5,0 x 10^5^ cells/sample) were stained with Zombie NIR viability dye (0.075 μl/100 μl) for 15 min at 4 °C followed by fixation with 4% PFA for 20 min at RT. Fixed cells were permeabilized with 0.5% Triton X-100 in PBS (10 min at RT) followed by 10 min incubation in Perm/Wash buffer (BD Biosciences, ref. 554,723). Primary antibody staining (anti-dsRNA J2, 1:1000 in Perm/Wash buffer) and secondary antibody staining (anti-mouse Alexa 647, 1:400 in Perm/Wash buffer) were performed for 1 h or 30 min at 4 °C followed by two washing steps. Cells were resuspended in BD CellWASH buffer (ref.349524) and acquired on a 4-laser Cytek Aurora (16V-14B-10YG-8R). Compensation was performed using single-stained controls, and fluorescence-minus-one (FMO) controls were used where appropriate. Data were analyzed using FlowJo (version 10.10.0). Doublets and dead cells were excluded based on forward and side scatter properties and viability dye staining. Gating strategies were established based on unstained and control samples. The gating strategy is presented in S14C Fig.

### Illustrations

All illustrations were done by us using Adobe Illustrator. The toxic warning symbol used to depict arsenite treatment was derived from Wikimedia Commons and is part of the public domain.

## Supporting information

S1 Fig Infection time course to assess SG formation during MHV infection. 17Cl1 mouse fibroblast cells were infected with MHV-A59 at an MOI of 10. Cells were fixed at indicated time points and subjected to indirect immunofluorescence staining targeting the main SG marker G3BP1 (green) and dsRNA (red) as an intermediate of viral replication.
(TIF)

S2 Fig Experimental setup for SG proteome comparisons based on proximity labeling and quantitative proteomics using a stable isotope labeling in cell culture (SILAC) approach. **(A)** For SILAC based comparisons of two conditions, two batches of 17Cl1:G3-A-G cells are propagated on medium either containing heavy isotope labeled L-arginine/L-lysine or unlabeled (“light”) L-arginine/L-lysine. Cells are seeded in 10 cm dishes. The treatments to compare are done, one on heavy cells and the other on light cells. APEX2-mediated proximity labeling is performed for both samples individually. After lysis, heavy and light lysates are mixed in a 1:1 protein ratio and a subsequent affinity purification of biotinylated proteins is performed on the mixture. Biotinylated proteins are identified by quantitative proteomics and a ratio of abundance between the compared treatments is calculated for each protein based on the intensity of heavy and light forms. **(B)** Five SILAC comparisons were performed to compare the SG proteome between oxidative stress (sodium arsenite treatment) and MHV infection. Comparison of each stressor with mock (comparison 1 and 4), comparison of each stressor with and without proximity labeling (comparison 2 and 5), and direct comparison of both stressors (comparison 3).
(TIF)

S3 Fig Comparison of different stress stimuli with and without APEX2-mediated proximity labeling. **(A)** Volcano plot shows the mean log2 fold change of proteins between sodium arsenite treated cells (oxidative stress) with (Ars APEX+) and without (Ars APEX**−**) APEX2-mediated proximity labeling of SG proteome plotted against the –log10 adjusted *p* value. Cells were treated with 250 μM NaAsO2 for 30 min. Proximity labeling was done with or without addition of biotin phenol. **(B)** Volcano plot shows the mean log2 fold change of proteins between MHV infected cells (oxidative stress) with (MHV APEX+) and without (MHV APEX**−**) APEX2-mediated proximity labeling of SG proteome plotted against the –log10 adjusted *p* value. Cells were infected with MHV-A59 (MOI 5). Proximity labeling was done at 8 hpi with or without addition of biotin phenol. Proteins from our SG associated proteins reference list are indicated in orange, main SG marker proteins are labeled and indicated in green, and viral proteins are labeled and indicated in red. The selected significance levels for log2 fold change (>=2 and <=−2) and adjusted *p* value (<0.05) are shown with dashed lines. The data underlying this Figure can be found in S2 Table and S1 Data.
(TIF)

S4 Fig Validation of APEX2-mediated proximity labeling and affinity purification in samples for mass spectrometry. **(A)** Immunofluorescence staining for biotinylated proteins (magenta) and dsRNA (red) of infected and proximity labeled samples for proteomics analysis. Heavy or light isotope labeled 17Cl1:G3-A-G cells (green) were grown in 10 cm dishes each containing one glass coverslip. Cells were infected with MHV or SFV at MOI 10, mock infected or treated with sodium arsenite. APEX2-mediated proximity labeling was performed at the indicated time points. After quenching the cells on the coverslips were fixed and stained. Images were acquired with a widefield fluorescence microscope and processed in Fiji. Scale bar: 20 µm. Presence of biotinylated (proximity labeled) proteins in **(B)** the individual lysates, and (C) the eluates and flowthroughs after affinity purification on streptavidin-coated magnetic beads was assessed by western blot analysis. Membranes were stained with HRP-conjugated NeutrAvidin.
(TIF)

S5 Fig Differential protein abundance analysis of the G3-A-G microenvironment between MHV- and SFV-infected cells and mock controls based on proximity labeling and quantitative proteomics. **(A)** Differential abundance of proteins between unstressed, proximity labeled (mock APEX+) and unstressed, unlabeled (mock APEX**−**) cells. Volcano plot displays the mean log2 fold change based on the heavy/light protein ratio between the conditions (*n* = 3) plotted against the -log10 adjusted *p* value. Proteins contained in the reference list for SG associated proteins are indicated in orange, the major SG markers are labeled and indicated in green. Dashed lines show the significance criteria for log2 fold change>log2(2.5) and <-log2(2.5), respectively, and adjusted *p* value < 0.05. (B) Log2 fold change density distribution of total identified proteins (gray bar plot), and the corresponding subset of previously with SG associated proteins based on a reference list (orange curve). Results for direct comparison of MHV and SFV infections at pre SG time point (top), peak SG time point (middle) and post SG time point (bottom), based on volcano plots in Fig 5. **(C)** Venn diagram visualizing overlap between significantly enriched protein fractions (red: mock APEX+/blue: mock APEX−), all identified proteins of this comparison (gray) and reference list of SG associated proteins (orange) for the unstressed proximity labeling control (mock). **(D)** Venn diagram visualizing overlap between significantly enriched protein fractions (red: MHV/blue: SFV), all identified proteins (gray) and reference list of SG associated proteins (orange) for the time point prior to SG formation (pre SG). The data underlying this Figure can be found in S3 Table and S1 Data.
(TIF)

S6 Fig Dissection of SG proteome induced by SFV, MHV or sodium arsenite using APEX2-mediated proximity labeling and quantitative proteomics. Schemes visualize the different stress stimuli compared to unstressed cells. **(A)** Different abundance of proteins between SFV-infected (5 hpi) and uninfected (mock), proximity labeled cells. **(B)** Different abundance of proteins between MHV-infected (8 hpi) and uninfected (mock), proximity labeled cells. **(C)** Different abundance of proteins between sodium arsenite treated and untreated (mock), proximity labeled cells. Volcano plots display the mean log2 fold change based on the heavy/light protein ratio between the conditions (*n* = 3) plotted against the −log10 adjusted *p* value. Proteins contained in the reference list for SG associated proteins are indicated in orange, the major SG markers are labeled and indicated in green. Dashed lines show the significance criteria for |log2FC| > log2(2.5), and adjusted *p* value < 0.05. For the MHV comparison the adjusted *p* value < 0.1 is additionally indicated (red dashed). The data underlying this Figure can be found in S4 Table and S1 Data.
(TIF)

S7 Fig Significantly enriched proteins in SG microenvironment during different stress stimuli. Venn diagrams visualizing overlap between significantly enriched protein fractions for the stress condition, all identified proteins in the respective comparison (gray), reference list of SG associated proteins (orange) and proximity labeling independent hits (proteins detected but not significantly enriched in “stressor with proximity labeling” vs. "stressor without proximity labeling” comparisons, gray) for **(A)** SFV, **(B)** MHV (less stringent *p* value criteria of <0.1 used) and **(C)** sodium arsenite. For selected sections, the corresponding lists of proteins are provided. Proteins in bold are found for all three stressors, colored proteins are only found for one virus (SFV: blue/MHV: red). **(D)** GO term enrichment analysis for significantly enriched proteins for each stressor using ClusterProfiler and rrvgo. Displayed are the top 5 most significant terms found in each subset of proteins for biological processes. Circle size indicates number of proteins (counts) identified for GO term. The data underlying this Figure can be found in S4 Table and S1 Data.
(TIF)

S8 Fig Comparison of G3-A-G microenvironment between different stress conditions and time points based on proximity labeling and quantitative proteomics. Volcano plots show the mean log2 fold change of proteins based on the individual conditions plotted against the –log10 adjusted *p* value. Proteins which have not been detected in one condition were imputed to receive a ratio between the compared conditions. Proteins from our SG associated proteins reference list are indicated in orange, main SG marker proteins are labeled and indicated in green, and viral proteins are labeled and indicated in red. Shapes indicate if protein was absent in one condition, while present in all replicates (*n* = 3) of the second condition (strict on/off), or at least present in 2 replicates of the second condition (on/off). The selected significance levels for |log2FC| ≥ 2 and adjusted *p* value (<0.05) are shown with dashed lines. The data underlying this Figure can be found in S4 Table and S1 Data.
(TIF)

S9 Fig Signal intensity of indicated SG proteins in SGs formed during MHV infection, SFV infection or sodium arsenite treatment based on the IF staining shown in Fig 7A–7G. Fluorescence microscopy images (3–6 per condition) from the experiments shown in Fig 7 were quantified by segmenting the SGs based on the G3-A-G channel using an Ilastik pixel prediction model and measuring the intensity of the indicated protein staining in the SGs. The mean intensities measured in the indicated number of SGs over multiple images were plotted as box plot and jitter plot for each condition. Statistical significance was tested by performing a Kruskal-Wallis test (indicated *p* value) and subsequent pairwise Wilcoxon test with *p* value adjustment by Benjamini-Hochberg FDR correction. *: *p* < 0.05; **: *p* < 0.01; ***: *p* < 0.001; ****: *p* < 0.0001; ns: nonsignificant. The data underlying this Figure can be found in S1 Data.
(TIF)

S10 Fig Subcellular localization of selected proteins in relation to SGs induced by oxidative stress, MHV or SFV infection. 17Cl1:G3-A-G (green) were either infected with MHV or SFV at an MOI of 10 or treated with 250 μM sodium arsenite for 30 min to induce SGs. Unstressed cells (mock) were included as negative control. MHV infected samples were additionally supplemented with HR2 peptide to prevent syncytia formation. Cells were fixed at indicated time points after infection/treatment and subjected to an indirect immunofluorescence staining with antibodies targeting the main SG markers **(A)** G3BP1, **(B)** TIA1, **(C)** UBAP2L, and additional protein **(D)** eIF4A3 (red channel). Additional staining for double stranded RNA (dsRNA, grays), an intermediate of virus replication, was done to visualize virus infection. Cell nuclei were stained with DAPI (blue). Scale bars: 20 μm
(TIF)

S11 Fig Subcellular localization of FUS over time in SFV infected cells. **(A)** 17Cl1:G3-A-G cells (green) were infected with SFV at an MOI 10, mock infected or treated with sodium arsenite. Cells were fixed at indicated time points; arsenite sample after 30 min, mock sample at 7 hpi. Indirect immunofluorescence staining was performed targeting FUS (red) and dsRNA (grays). Scale bar: 20 µm. **(B)** Quantification of average fluorescence intensity of FUS signal on a per-image base. The cytoplasm was segmented based on the G3-A-G channel and the area and integrated density were measured. For each, the mean average intensity based on three images is plotted for each condition. The dots represent the average intensity for the individual images; the error bar represents the standard deviation. *P*-value of one-way ANOVA over all conditions indicated. A paired *t* test was performed between the mock condition and each infected/treated condition. *: *p* < 0.05; **: *p* < 0.01; ns: nonsignificant. The data underlying this Figure can be found in S1 Data.
(TIF)

S12 Fig Subcellular localization of PTBP1 over time in SFV infected cells. **(A)** 17Cl1:G3-A-G cells (green) were infected with SFV at an MOI 10, mock infected or treated with sodium arsenite. Cells were fixed at indicated time points; arsenite sample after 30 min, mock sample at 7 hpi. Indirect immunofluorescence staining was performed targeting PTBP1 (red) and dsRNA (grays). Scale bar: 20 µm. **(B)** Quantification of average fluorescence intensity of PTBP1 signal on a per-image base. The cytoplasm was segmented based on the G3-A-G channel and the area and integrated density were measured. For each, the mean average intensity based on three images is plotted for each condition. The dots represent the average intensity for the individual images; the error bar represents the standard deviation. *P*-value of one-way ANOVA over all conditions indicated. A paired *t* test was performed between the mock condition and each infected/treated condition. *: *p* < 0.05; **: *p* < 0.01; ns: nonsignificant. The data underlying this Figure can be found in S1 Data.
(TIF)

S13 Fig Subcellular localization of TAF15 over time in SFV infected cells. **(A)** 17Cl1:G3-A-G cells (green) were infected with SFV at an MOI 10, mock infected or treated with sodium arsenite. Cells were fixed at indicated time points; arsenite sample after 30 min, mock sample at 7 hpi. Indirect immunofluorescence staining was performed targeting TAF15 (red). Scale bar: 20 µm. **(B)** Quantification of average fluorescence intensity of TAF15 signal on a per-image base. The cytoplasm was segmented based on the G3-A-G channel and the area and integrated density were measured. For each, the mean average intensity based on three images is plotted for each condition. The dots represent the average intensity for the individual images; the error bar represents the standard deviation. *P*-value of one-way ANOVA over all conditions indicated. A paired *t* test was performed between the mock condition and each infected/treated condition. *: *p* < 0.05; **: *p* < 0.01; ns: nonsignificant. The data underlying this Figure can be found in S1 Data.
(TIF)

S14 Fig Gating strategy and scatterplots for dsRNA analysis by flow cytometry. 17Cl1 cells were infected either **(A, B)** with SFV (MOI 10) or MHV (MOI 10), or **(C)** with MHV-nsp15 (MOI 5) or MHV (MOI 10), and samples were collected at 3, 5, and 8 hours post-infection (hpi). After a viability staining (Zombie NIR) to exclude dead cells, cells were fixed with 4% PFA followed by permeabilization with triton, and intracellular dsRNA staining to identify infected (dsRNA⁺) cells. Data were acquired on a Cytek Aurora, and gates were set using uninfected controls. (A) Gating strategy: Cells were gated on FSC/SSC, singlets, and viable cells prior to analyzing the intracellular dsRNA signal within the cells. (B-C) Representative scatter plots of three independent experiments show a time-dependent increase in dsRNA⁺ 17Cl1 cells following infection with SFV, MHV, or MHV-nsp15, but not in uninfected controls. Differences in dsRNA⁺ cell frequencies and intracellular dsRNA accumulation in 17Cl1 cells indicated distinct viral mechanisms among MHV, MHV-nsp15, and SFV. The data underlying this Figure can be found in S1 Data and on the Zenodo repository (https://doi.org/10.5281/zenodo.21507492).
(TIF)

S15 Fig Subcellular localization of mATF4 mRNA as representative host mRNA in relation to SGs induced during MHV infection using smRNA FISH. 17Cl1:G3-A-G cells (blue) were infected with MHV at an MOI of 0.1. Medium was then supplemented with HR2 peptide to prevent syncytia formation. Cells were fixed at indicated time points and subjected to smRNA FISH with a probe targeting the mouse activating transcription factor 4 mRNA (mmATF4, green), and a probe targeting MHV genomic RNA (vRNA, magenta). Line plots display measured intensity of G3-A-G, mmATF4 and vRNA channels along the selected line (white) indicated in the inset. Cell nuclei were stained with DAPI (grays, only in merge). Scale bar: 5 μm. The data underlying this Figure can be found in S1 Data.
(TIF)

S16 Fig Verification of subcellular localization of MHV and SFV vRNA in relation to SGs visualized by antibody staining of TIA1. 17Cl1 wildtype cells were infected with MHV or SFV at an MOI of 0.1 or 10 or treated with sodium arsenite for 30 min. Medium of MHV samples was then supplemented with HR2 peptide to prevent syncytia formation. Cells were fixed at indicated time points and subjected to smRNA FISH with probes targeting **(A)** MHV ORF1a (magenta), and MHV S gene (red) or **(B)** SFV ORF1 (red). Cells were additionally stained for TIA1 (green). Line plots display measured intensity of TIA1, MHV ORF1a, and MHV S gene or SFV ORF1 channels along the selected line (white) indicated in the inset. Cell nuclei were stained with DAPI (grays, only in merge). Scale bar: 20 μm/10 μm (inset). **(C)** Relative mean intensity of vRNA probes between SGs and surrounding local cytoplasm was measured based on 3–7 images and the log2 transformed values are plotted for each condition and target vRNA. Number of SGs per condition indicated. Statistical analysis with pairwise Wilcoxon test and Benjamini-Hochberg correction. ****: *p* < 0.0001, **: *p* < 0.01, *: *p* < 0.05. The data underlying this Figure can be found in S1 Data.
(TIF)

S17 Fig Colocalization analysis by intensity line plots based on smRNA-FISH images of MHV-infected and arsenite treated cells presented in Figure 9A. Cutouts of images shown in Fig 9A. MHV-infected (MOI 10) and/or arsenite treated (30 min, 250 μM) or uninfected 17Cl1:G3-A-G cells (green) fixed at **(A)** 4 hpi or **(B)** 8 hpi. Scale bar: 5 µm. Line plots display measured intensity of G3-A-G (green), ORF1a (magenta) and S gene (red) channels along the selected line (white, left to right) indicated in the images. Cell nuclei were stained with DAPI (grays, only in merge). The data underlying this Figure can be found in S1 Data.
(TIF)

S1 Movie SG formation during MHV and SFV infections monitored by live-cell imaging. 17Cl1 cells expressing a G3BP1-APEX2-GFP fusion protein (green) were infected with MHV or SFV at an MOI of 10, or mock infected. MHV infected samples were additionally supplemented with HR2 peptide to prevent syncytia formation. Cells were imaged every 10 min on a DeltaVision widefield fluorescence microscope equipped with an on-stage incubator and 5% CO2 supply. Scale bar: 20 μm
(MP4)

S2 Movie SG formation during MHV-nsp15 and MHV wildtype infections monitored by live-cell imaging. 17Cl1 cells expressing a G3BP1-APEX2-GFP fusion protein (green) were infected with MHV-nsp15 (H277A) or MHV wildtype at an MOI of 10, or mock infected. Samples were additionally supplemented with HR2 peptide to prevent syncytia formation. Cells were imaged every 20 min on a DeltaVision widefield fluorescence microscope equipped with an on-stage incubator and 5% CO2 supply. Scale bar: 20 μm
(MP4)

S1 Table Reference list of SG associated proteins related to Figures 2, 3, 5, and S3, S5–S8. Proteins are derived from the RNA granule database [22]. List was downloaded on the 9.12.2025 and only proteins with high confidence (tire 1) were included.
(CSV)

S2 Table MaxQuant output for first APEX2-based SG proteome analysis comparing MHV and sodium arsenite, related to Figures 2, 3, and S3. The file contains protein intensities including corresponding analysis based on individual conditions (Analysis - imputation) and heavy/light ratio (MS data), an experimental design overview, and additional information.
(XLSX)

S3 Table MaxQuant output for second APEX2-based SG proteome analysis comparing MHV and SFV, related to Figures 5, 6, S5 and S8. The file contains protein intensities including corresponding analysis based on heavy/light ratios (MS data - ratios/Analysis - normalized ratios) and individual conditions (MS data - individual conditions/Analysis - imputation), an experimental design overview, and additional information.
(XLSX)

S4 Table MaxQuant output for second APEX2-based SG proteome analysis comparing MHV, SFV and arsenite against mock, related to S6 and S7 Figs. The file contains protein intensities including corresponding analysis based on heavy/light ratios (MS data – ratios/Analysis - normalized ratios) and individual conditions (MS data - individual conditions/Analysis - imputation), an experimental design overview, and additional information.
(XLSX)

S5 Table Sequences of oligonucleotides for fluorescence in-situ hybridization targeting MHV viral RNA, SFV viral RNA, or mouse ATF4 mRNA. Oligonucleotides are complementary either to MHV ORF1a nsp3 (MHV_ORF1a), MHV spike gene (S_gene), SFV ORF1 nsp2 (SFV_nsp) or the mouse ATF4 mRNA (mmATF4).
(XLSX)

S1 Raw Images The raw blot images for the western blot results shown in Figure 1C, 4F, and S4B, S4C recorded on a FusionFX imager. Left images show raw image of luminescence, right images show composite with ladder.
(PDF)

S1 Data File containing numerical data for all plots shown in this study. (XLSX)

## Abbreviations

ATF4: activating transcription factor 4
BP: biotin phenol
CB: confocal buffer
CC: cellular compartment
DMVs: double membrane vesicles
ds: double-stranded
eIFs: eukaryotic translation initiation factors
FMO: fluorescence-minus-one
G3BP1: GTPase-activating protein-binding protein 1
hnRNP: heterogeneous nuclear ribonucleoproteins
hpi: hours post-infection
HRP: horse radish peroxidase
ISR: integrated stress response
LC-MS/MS: liquid chromatography tandem mass spectrometry
MEM: minimum essential medium
MF: molecular function
MHV: mouse hepatitis virus
MLE: Maximum Likelihood Estimation
PBs: processing bodies
PKR: protein kinase R
SFV: Semliki Forest virus
sgmRNA: subgenomic mRNA
SG: stress granules
SILAC: stable isotope labeling of amino acids in cell culture
smRNA-FISH: single molecule RNA fluorescence in situ hybridization
TIA-1: T-cell restricted intracellular antigen-1
UBAP2L: ubiquitin associated protein 2 like.
