# Supplementary material for: Stress granules formed during infections with RNA viruses have significant virus-specific differences in generation and composition
Source: PLoS Biol. 2026 Aug 14;24(8):e3003363. doi: 10.1371/journal.pbio.3003363 (PMC13497286; doi:10.1371/journal.pbio.3003363)

Western blot from Figure 1C

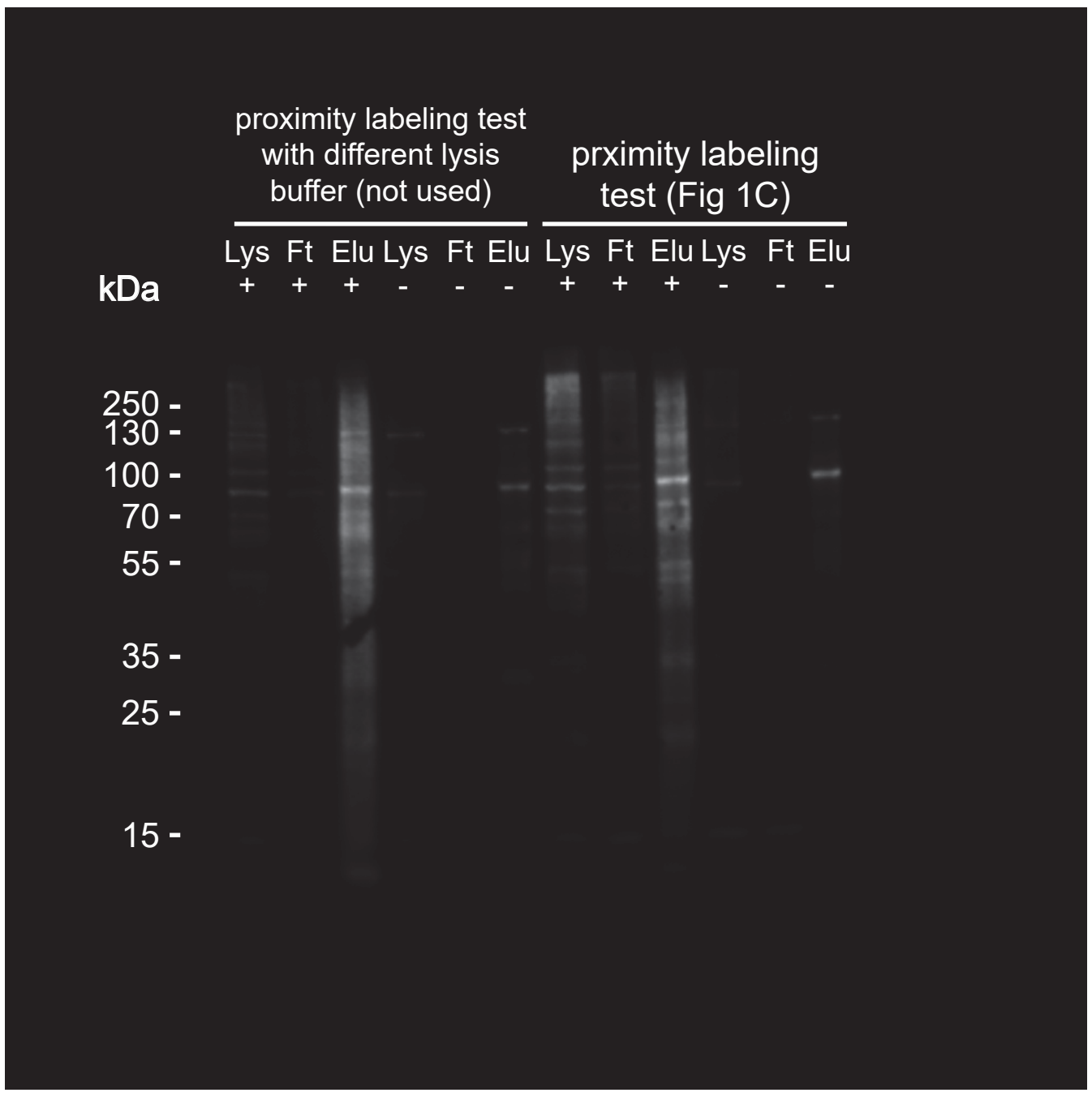

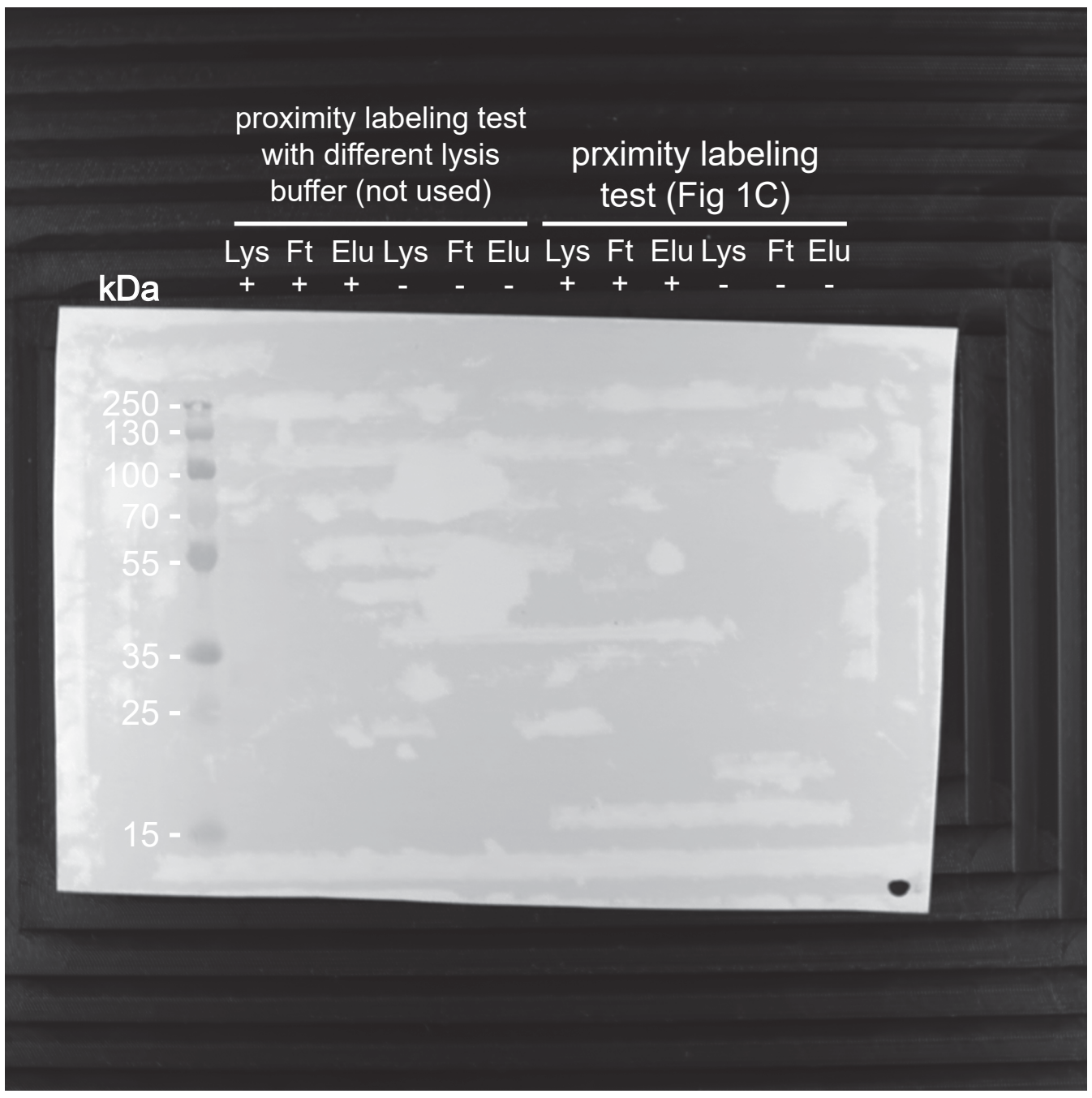

Western blots from Figure 4F

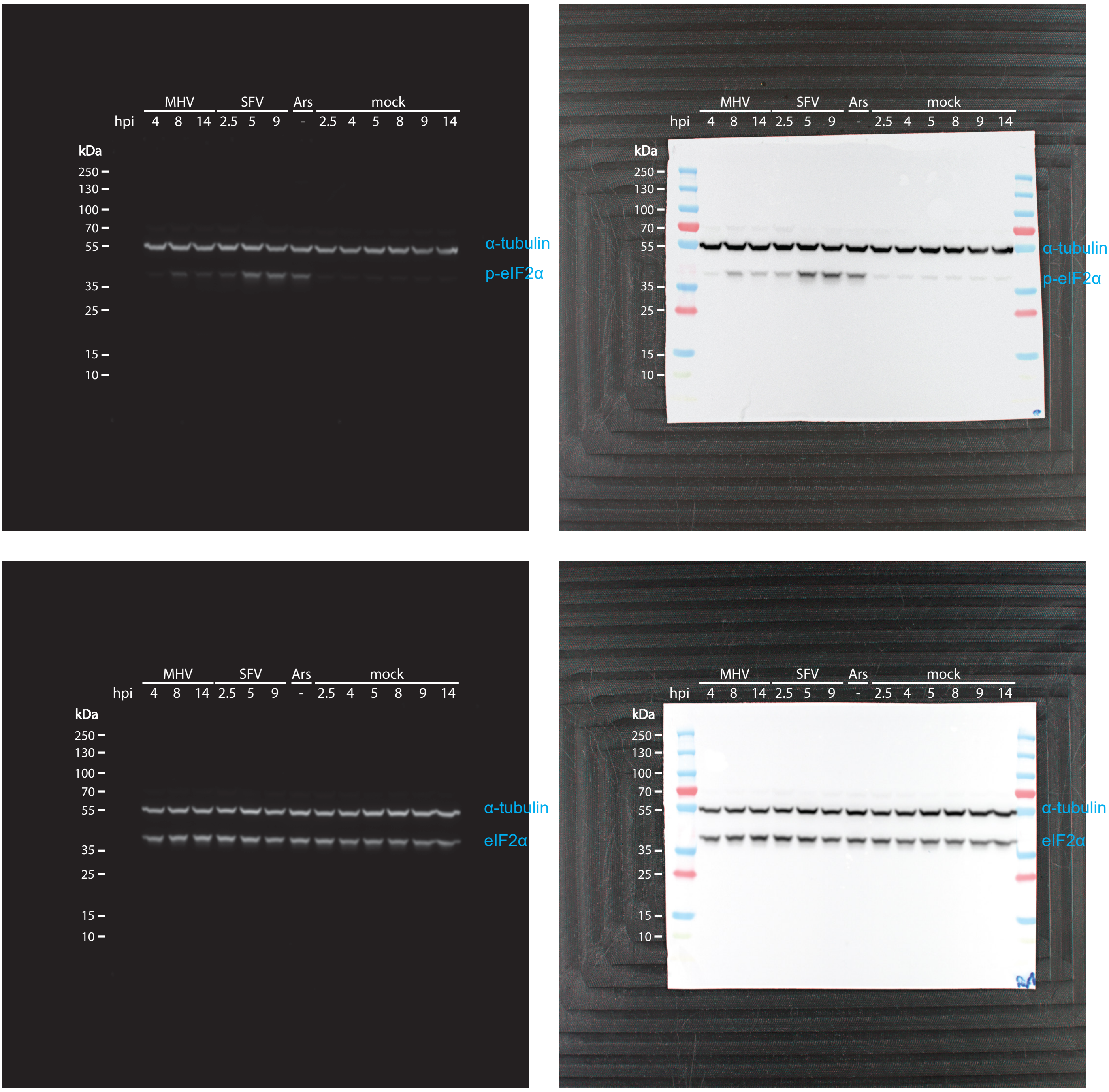

Western blots from Supplementary 4B/C

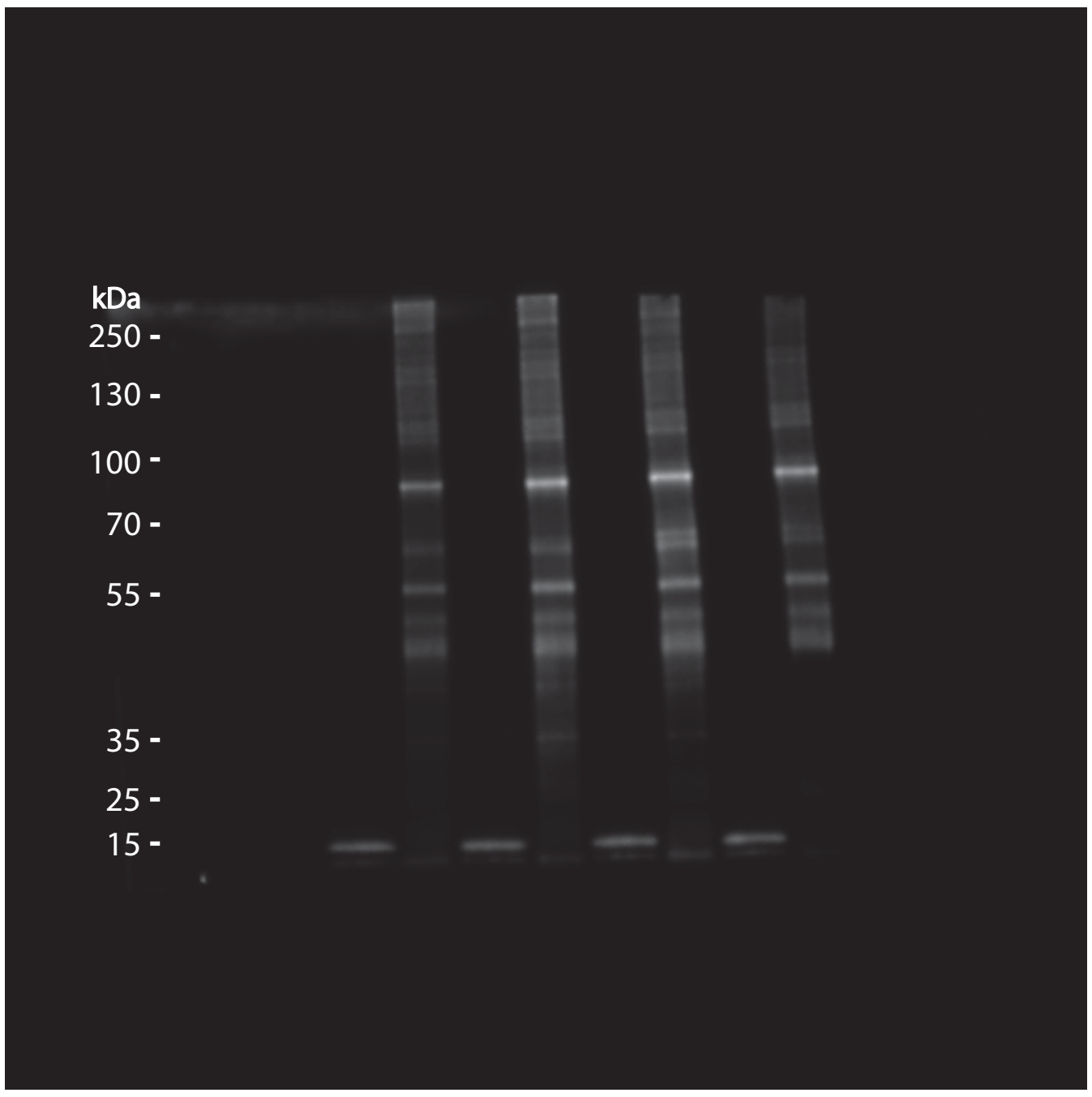

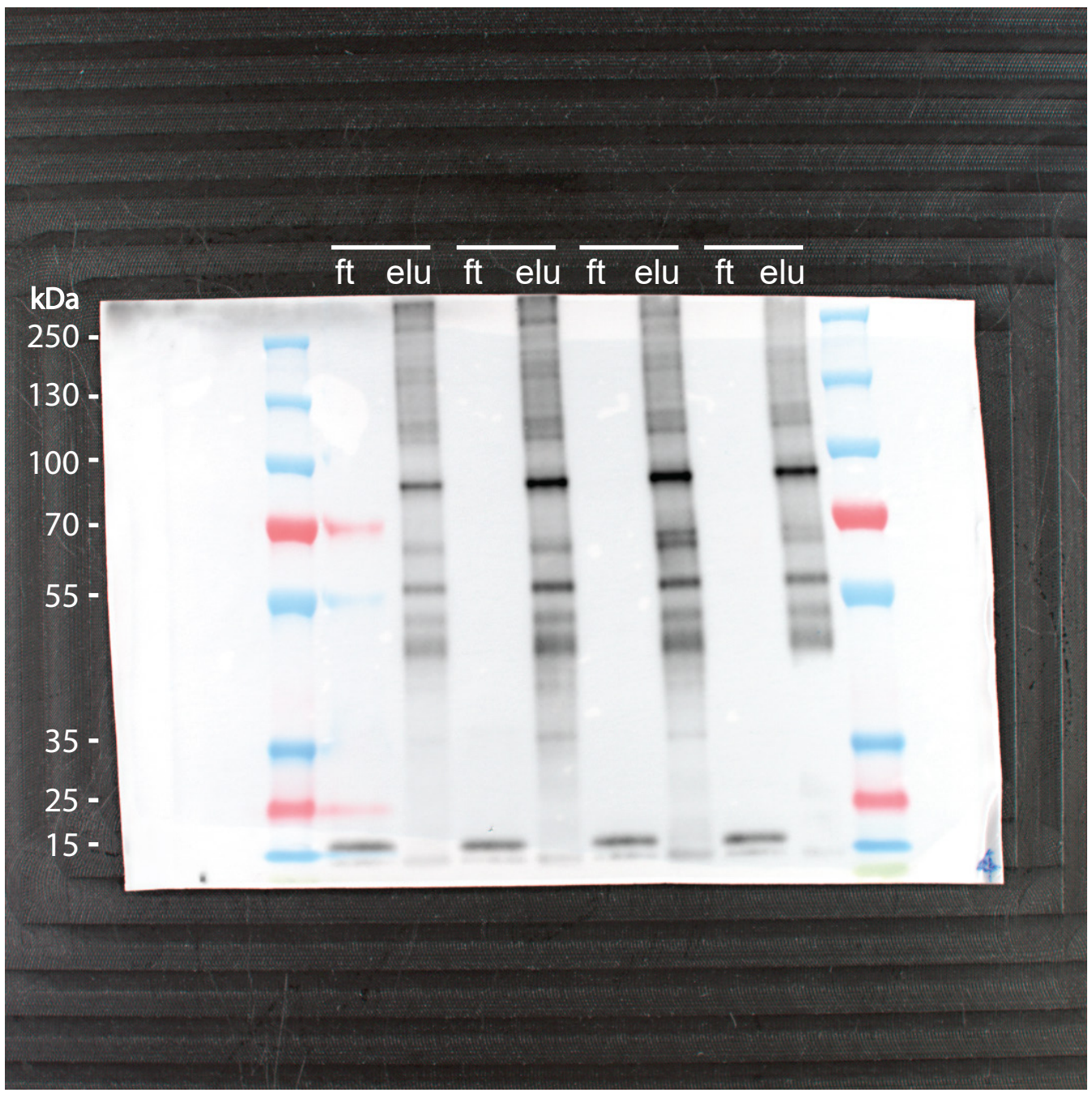

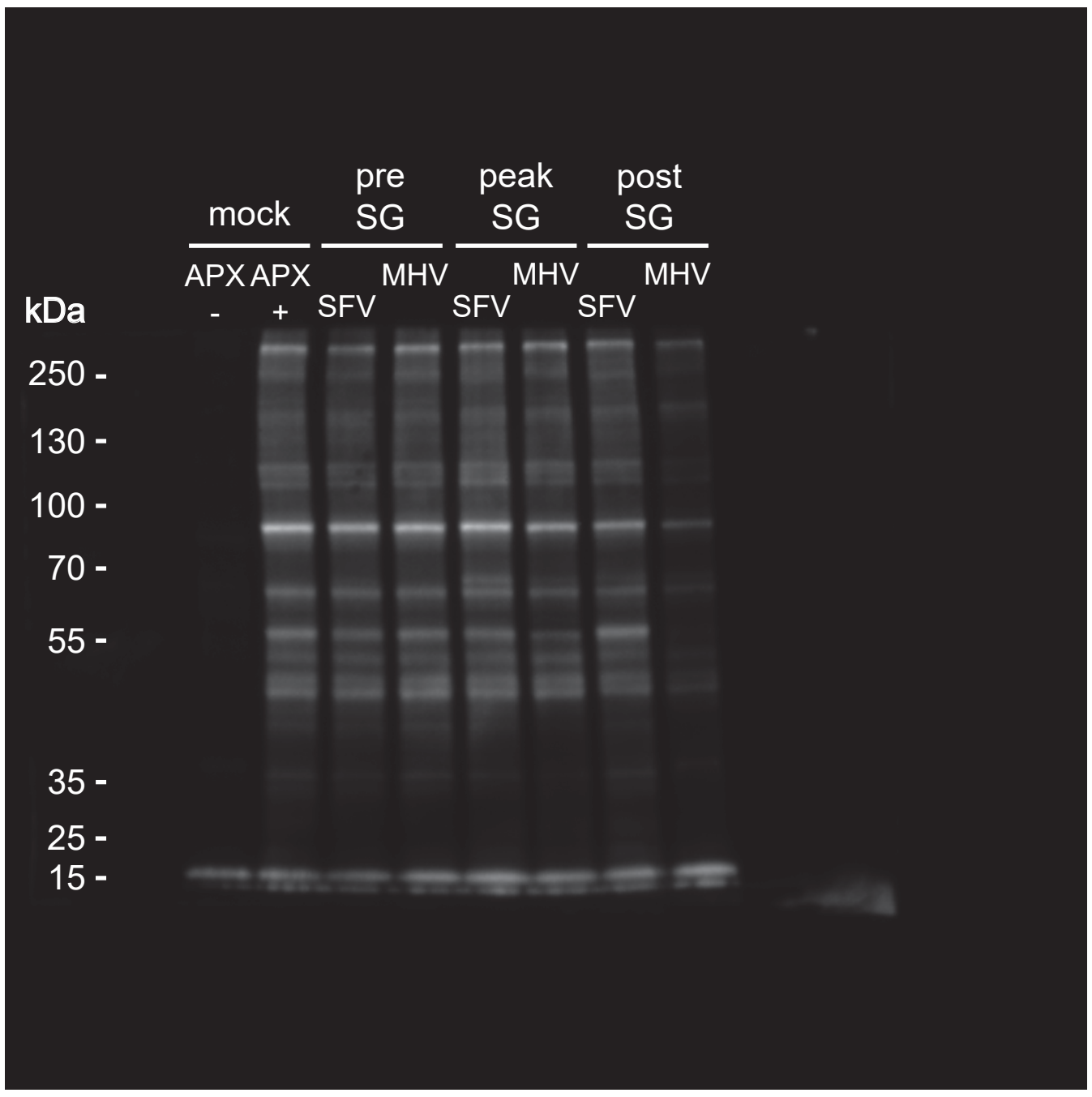

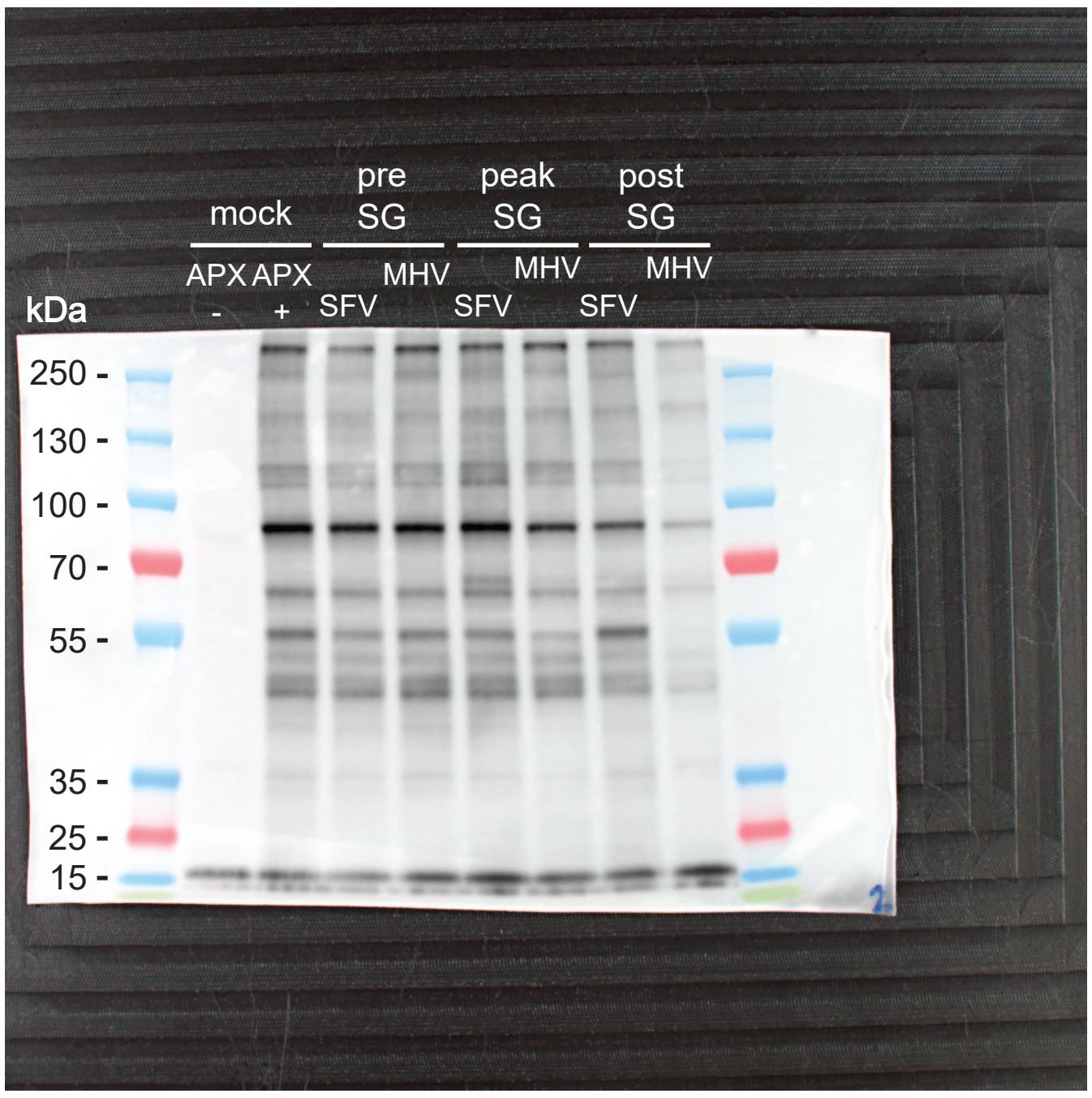

Supplement: Supplementary file 1 [file pbio.3003363.s025.pdf]
