# Supplementary material for: Stress granules formed during infections with RNA viruses have significant virus-specific differences in generation and composition
Source: PLoS Biol. 2026 Aug 14;24(8):e3003363. doi: 10.1371/journal.pbio.3003363 (PMC13497286; doi:10.1371/journal.pbio.3003363)
Supplement: Supplementary file 3 [file pbio.3003363.s030.pdf]

Reviewer 1:

This manuscript presents a compelling comparative analysis of stress granules (SGs) induced by MHV and SFV infection, employing sophisticated proximity labeling and proteomic approaches. The central finding—that MHV induces atypical, non-antiviral SGs—is significant and challenges the prevailing paradigm of SGs as universal antiviral hubs. The work is technically sound and well-executed. However, several key concerns must be addressed to solidify the major conclusions and fully contextualize the findings within the field.

We thank the reviewer for the positive comment, and we have performed additional experiments to solidify the major conclusions according to the specific comments below.

Major comments

1. *Experimental Validation of Early SG Suppression by MHV: The conclusion that MHV prevents SG formation early in infection is potentially confounded by the use of a single viral inoculum (MOI=10). To robustly support this claim, infection with a higher viral dose (e.g., MOI=100) must be performed. The number of SGs formed at the same early time point under both conditions should be statistically compared and the viral inocula should be precisely quantified (e.g., in TCID50).*

   Due to technical challenges, we did not manage to produce a virus stock with a titer high enough to carry out an infection at MOI 100. However, we have now added the quantification, including statistics, of the number and size of SGs in different settings (Fig 4E,7H,10B). Figure 7H is displaying now the sizes of SGs induced by sodium arsenite, SFV, and MHV. We have also added detailed quantifications of the intensity of IFA stainings of detected SG components, such as eIF4G1, eIF3a, PTBP1, TAF15, and others (Suppl. Fig. 9).

   Furthermore, we addressed the question of whether MHV prevents SG formation by additional experiments. These involve induction of SGs by sodium arsenite in MHV infected cells and by co-infection experiments with SFV and MHV. We found that MHV does not prevent the formation of canonical SGs induced by sodium arsenite treatment early in infection (4hpi), nor does it prevent SG formation in SFV and MHV co-infected cells (4hpi). These results indicate that the suppression of SGs at early time points is not based on an active inhibition of these granules by MHV.

   The results of the sodium arsenite treatment of MHV-infected cells are presented in an additional figure (Fig. 10A/B) and the corresponding changes in the main are in lines 497-512 (500-515 in track change manuscript).

The results of the SFV – MHV coinfection are presented in Fig. 10C-F and demonstrate that the kinetics of SG formation is dictated by SFV and that MHV does apparently not prevent SG formation at early time points post co-infection (5 hpi). Notably, as outlined below (comment 2), the SGs induced in SFV and MHV co-infected cells contain only SFV vRNA but not MHV vRNA.

2. *Demonstration of Viral RNA Exclusion from MHV SGs: The hypothesis that MHV evades innate immunity by excluding its vRNA from SGs is a cornerstone of the paper. This requires direct experimental validation. Immunofluorescence experiments co-staining for vRNA and SG markers (e.g., G3BP1) must be added, with quantitative co-localization statistics presented (e.g., in a histogram format in a supplemented Figure 7). This is critical evidence to visually and statistically demonstrate the exclusion.*

   We agree with the reviewer that the exclusion of vRNA from SGs in MHV-infected cells is a cornerstone of the paper. We have therefore conducted additional experiments to corroborate this finding.

   First, we have performed additional IFA and smRNA FISH to validate and quantify the presence or absence of vRNAs in SFV- or MHV-infected cells. As shown now in the new Suppl. Fig. 16, IFA, and corresponding intensity line plots and quantification of fluorescence intensity, corroborate that indeed MHV vRNA is absent in MHV-induced SGs, while SFV vRNA is readily detectable in SFV-induced SGs.

   Second, we have assessed if vRNA can be detected in sodium arsenite-induced SGs in MHV-infected cells. As shown in Figure 10A/B, and Suppl. Fig.17, treatment of MHV-infected cells with sodium arsenite during the early phase of infection (4 hpi) results in SG formation, while MHV-infected cells that were not treated with sodium arsenite show, as before, almost no SGs (Fig. 10A/B). This indicates that MHV may not actively prevent SG formation under these conditions. In Suppl. Fig. 17, we have assessed the localization of MHV vRNA. At both time points post infection that were analyzed (4hpi and 8hpi) we observed that MHV vRNA has a distinct localization compared to SGs that were either induced in MHV-infected cells 8hpi, or in MHV-infected and sodium arsenite treated cells (analyzed at 4hpi and 8hpi).

   Third, we have assessed if vRNA can be detected in SGs of MHV and SFV co-infected cells. As shown in Fig.10C-F, SG formation in co-infected cells is already observed at early time points post infection, suggesting that the kinetics of SG formation is dictated by SFV, and that MHV does apparently not actively inhibit SG formation in SFV and MHV co-infected cells at this early time point (5hpi).

Strikingly, SGs induced in co-infected cells do contain SFV vRNA but not MHV vRNA.

These new findings are included in the manuscript in lines 497-528.

3. *Physiological Relevance of SFV NSP3-G3BP1 Interaction: The proposed mechanism for SFV—that NSP3-mediated SG formation is antiviral—requires functional validation. To distinguish these as "classic" antiviral SGs from MHV's atypical ones, the authors should overexpress SFV NSP3 and G3BP1 and measure the subsequent impact on viral replication (e.g., through viral titer or RNA copy number assays). Demonstrating that this interaction partially inhibits viral growth would provide crucial physiological evidence for its antiviral role.*

In this study, we did not focus on a closer investigation of the SFV nsP3-G3BP1 interaction since this already has been addressed in several previous studies (1,2,3). Although we did not perform overexpression experiments of nsP3 or G3BP1, we further investigated the potential effect of SGs on virus growth using a G3BP1 inhibitor. As outlined below (specific comment 9 of reviewer 3), we did not observe any increase of MHV replication under conditions of G3BP1 inhibition, further corroborating the idea that SGs may not exert pronounced antiviral functions during MHV infection.

1) Panas MD, Varjak M, Lulla A, Eng KE, Merits A, Hedestam GBK, et al. Sequestration of G3BP coupled with efficient translation inhibits stress granules in Semliki Forest virus infection. Mol Biol Cell. 2012;23(24):4701–12.
2) Kedersha N, Panas MD, Achorn CA, Lyons S, Tisdale S, Hickman T, et al. G3BP–Caprin1–USP10 complexes mediate stress granule condensation and associate with 40S subunits. J Cell Biol [Internet]. 2016 Mar 28;212(7):845–60.
3) Jayabalan AK, Adivarahan S, Koppula A, Abraham R, Batish M, Zenklusen D, et al. Stress granule formation, disassembly, and composition are regulated by alphavirus ADP-ribosylhydrolase activity. Proc Natl Acad Sci [Internet]. 2021 Feb 9;118(6).

4. *Definition and Discussion of Atypical MHV SGs: It remains unclear if the MHV-induced structures are bona fide SGs or a distinct class of virus-specific RNP condensates. The discussion must be expanded to directly address this point, explicitly contrasting the proteome and proposed function of MHV SGs with the canonical, antiviral SGs formed during Sendai virus infection. The discussion should focus on the discovery of these non-immunogenic SGs and their significance for viral immune evasion.*

We thank the reviewer for this comment and agree that we should further emphasize and discuss the “atypical” MHV-induced stress granules. We have

now extended the discussion and contrasted “antiviral” SG induced by SFV and other viruses, such as Sendai virus, with those induced by MHV.

Minor comments

1. *The proteomic data reveals a fundamental difference: SFV RNA is incorporated into SGs while MHV RNA is excluded. The manuscript should discuss the potential mechanism for this selectivity. Is it determined by intrinsic features of the viral RNAs (e.g., sequence, structure) or is it actively mediated by specific viral proteins?*

   We thank the reviewer for this comment and have included potential mechanisms in the discussion. These are clearly speculative mechanisms and need to be addressed in future studies, but we think this will stimulate the field. The corresponding text in the discussion section is now in lines 616-632 (621-637 in track change manuscript).

   “Especially, the exclusion of MHV vRNA from SGs contrasting the observed accumulation for SFV vRNA raises the question on underlying mechanisms. As MHV vRNA is also excluded from SGs induced by sodium arsenite and SFV, it does not seem to be a specific characteristic of MHV-induced atypical SGs. A more likely explanation could be differences in the availability of vRNA in the cytoplasm between SFV and MHV. Alphaviruses like SFV replicate in single-membraned spherules with an opening at one end known to “leak” vRNA into the cytoplasm and activating host sensors like PKR early in infection (15). In contrast, MHV and other CoVs replicate in more complex double membraned vesicles (DMVs) with viral protein pores as only connections to the cytoplasm (47,48). Furthermore, as seen with our dsRNA measurements, MHV seems to produce lower levels of dsRNA and possesses mechanisms to regulate vRNA amounts. Another mechanism of vRNA exclusion could be the interaction with viral proteins like the vRNA-binding N, or the efficient translation potentially mediated by nsp1 preventing the recruitment into SGs. Further investigations are needed to assess which mechanism is driving MHV vRNA exclusion from SGs. Nevertheless, the striking difference in vRNA accumulation between SFV and MHV is of special interest because some suggested antiviral mechanisms of SGs are associated with the recruitment of vRNA to these condensates.“

2. *The discussion should address the potential link between the temporal differences in SG assembly (early vs. late) and viral replication strategies. The authors are encouraged to discuss whether this timing reflects an active viral*

strategy to evade or suppress host translation and innate sensing at critical phases of the infection cycle.

In our previous discussion, we briefly discussed the difference in SG appearance between both viruses and its potential implications on SG function and host-virus interactions. To make this point clearer, we added a short paragraph specifically discussing the delayed SG formation during MHV infection as viral evasion strategy (lines 673-680; 684-691 in track changes manuscript).

"The striking differences in the timing of SG appearance in relation to virus growth between MHV and SFV indicate different replication strategies of these viruses. While SFV seems to quickly overwhelm the cell and employ countermeasures to cope with activated host responses, like the disassembly of SGs or the independence form eIF2, MHV seems to remain undetected for a longer period while establishing the infection. The weak stress stimulus combined with the late time point of SG formation during MHV infection might give rise to the altered proteome of the atypical SGs characterized in this work."

3. *Figure 1: The green fluorescent signals observed in the 17Cl1 wild-type negative control cells in Figure 1 require explanation. The authors must clarify the source of this signal (e.g., autofluorescence, non-specific antibody binding) to ensure proper interpretation of the data.*

   The observed signal in the 17Cl1 wildtype cells is caused by autofluorescence. We agree that an explanation of the observed signal improves the interpretation of the presented data and therefore we added a short explanation in the corresponding paragraph in the results section (line 133-135; 134-136 in track changes manuscript).

   "A weaker signal in the G3-A-G channel can also be observed for the wildtype cells, which is caused by autofluorescence of the sample."

4. *The legend for Figure 4 is incomplete and must be completed to provide all necessary information for the reader to interpret the figure accurately.*

   We added the missing information to the legend of figure 4.

Reviewer 2:

Lang et al present a detailed examination of stress granules, induced by two viruses, mouse hepatitis coronavirus (MHV) and Semliki Forest alphavirus (SFV). Controls

include sodium arsenite, a standard treatment used to induce stress granules. Stress granules proteins are identified by fusing the major stress granule initiator protein G3BP1 with the peroxidase APEX, which allows for a very short time period of labeling of the vicinal proteins. The SILAC labeling technique is used to identify the differences between the compared conditions. Additionally, fluorescence microcopy is used to verify the behavior of the major stress granules proteins in cells. SFV induces stress granules early in infection, as previously shown by Gerald McInerney and others, and the granules are soon disassembled. Interestingly, MHV induces stress granules late in infection, when the virus titer is reaching its peak, and these granules are also disassembled. Nicely, Lang et al examine the stress granule microenvironment at three different time point during infection: before the granules appear, at peak granule time point, and after visible granules have disappeared. Importantly, the results are similar for both viruses before the granules appear. Then, SFV induces a strong stress response, which resembles arsenite induces stress, and many known stress granule proteins are found in the vicinity of G3BP1. MHV appears to induce a weaker stress response, and a smaller number of known stress proteins are found. Additionally, many new proteins are identified that can now be linked to MHV-induces stress and could also play general roles. The functions of stress granules during virus infection continue to be debated, even for the well-studied SFV. Interestingly, due to the different time scales, SFV granules could perhaps be linked to virus replication and nonstructural proteins, and MHV granules could perhaps be linked to the large-scale production of structural proteins. Furthermore, Lang et al find that viral RNA is present in the SFV-induced granules but absent from the MHV-induced granules.

We thank the reviewer for the positive comments

Major comments

1. *The G3BP1-APEX-GFP fusion protein appears to be expressed in addition to the cellular G3BP1 in the cell line constructed for this work. As background information, how does the expression level of the fusion protein compare to that of endogenous G3BP1? Overexpression could conceivably affect some properties of the stress granules, but one is reassured by the fact that the time courses of SG formation and disappearance during virus infections are similar to previously published work.*

   In our cell line, the G3BP1-APEX2-GFP fusion protein is expressed additionally to the endogenous G3BP1. To prevent unwanted effects of G3BP1 overexpression on SG formation (e.g. spontaneous SG formation in absence of stressor as shown previously (1)), our cell line only expresses G3BP1-APEX2-GFP at a low level, which we could verify with a Western blot analysis shown below. We stained

lysates from 17Cl1:G3AG or wildtype (wt) cells either with an antibody against G3BP1 alone or together with an antibody against GFP. Based on the low protein level, the absence of visible G3BP1-APEX2-GFP accumulations in unstressed cells and the SG formation time points in line with previous studies on MHV- and SFV-induced SGs let us conclude that there are now significant effects on SG characteristics due to the additional expression of G3BP1.

1) *Reineke, L. C., Dougherty, J. D., Pierre, P. & Lloyd, R. E. Large G3BP-induced granules trigger eIF2α phosphorylation. Mol. Biol. Cell 23, 3499–3510 (2012).*

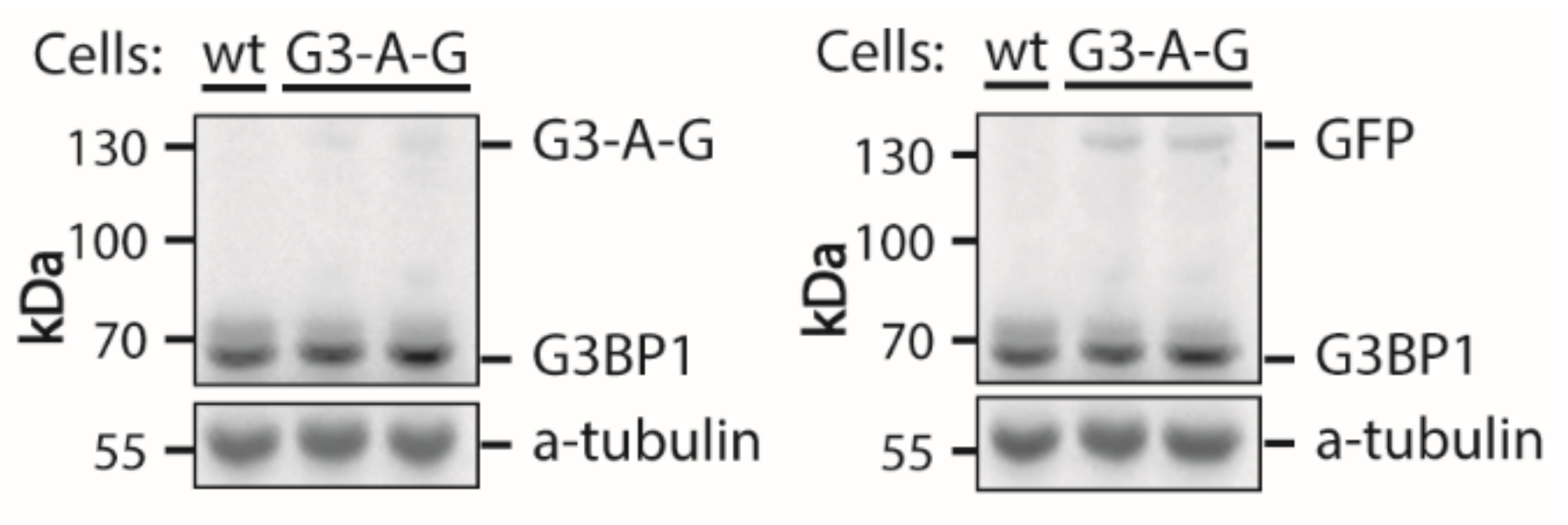

Assessing the expression level of G3BP1-APEX2-GFP (G3-A-G) fusion protein. *Lysates from 17Cl1:G3AG cells (2x) or wildtype cells (wt) were subjected to Western blot analysis targeting G3BP1 (left panel) or G3BP1 and GFP (right panel). Alpha-tubulin was used as loading control.*

2. *Line 204 glycosylation. Is this set of proteins specifically involved in cytoplasmic O-GlcNAcylation of proteins? And not involved in the glycosylation of secretory proteins, which takes place in compartments topologically separated from the cytoplasmic stress granules and G3BP1.*

   The GO terms for glycosylation in our analysis are associated with four detected proteins (Ext2, Pomgnt2, B4gat1and B4gat5). These proteins are involved in the glycosylation of secretory proteins and mainly localize to the ER and secretory pathway according to UniProt.

Minor comments

1. *Refer to all supplementary figures and other material in the text, in the order the figures (etc.) are numbered. Some might now be missing.*

   We checked all supplementary figures and material and referenced the missing ones in the text.

2. Corrections to Figure legends:
   -Fig. 4 panels E and F missing

-Fig. 6 check panels B and C for order
-S1 Movie check the wording of the title

We corrected the Figure legends.

Reviewer 3:

In this manuscript, the authors explore the composition of stress granules (SGs) formed during mouse hepatitis virus (MHV). Leveraging APEX2-based proximity labeling combined with quantitative proteomics, they first compare MHV-induced SGs to those triggered by arsenite, a commonly used inducer of oxidative stress. To uncover virus-specific characteristics, a parallel comparison is made with SGs formed during infection by Semliki Forest virus (SFV), another positive-sense single-stranded RNA virus. Their results reveal both stress- and virus-specific differences in SG composition, underscoring the unique nature of virus-induced SGs, including distinct patterns of viral protein and viral RNA (genomic and/or subgenomic) colocalization. The study concludes by examining temporal changes in SG composition throughout the infection. This study is thoughtfully designed and rigorously executed. A major strength of the manuscript is its direct comparison of different viruses within the same cellular context, utilizing an APEX-based proximity labeling approach combined with SILAC mass spectrometry to reveal virus-specific differences in SG composition would provide valuable insights into the interplay between the viruses and the cellular stress response. The following points may help to strengthen the manuscript.

We thank the reviewer for the positive comments.

Major comments

1. *The mouse cell line 17Cl1:G3AG used in this study exhibits SG-like foci even under mock (unstressed) conditions, as shown in Video S1. Since overexpression of G3BP1 frequently induces spontaneous self-condensation in the absence of stress, this phenomenon could potentially bias the proteomic enrichment analysis, leading to an underestimation of proteins enriched relative to mock. How many cells display spontaneous SG formation? Assuming this proportion is minimal, the authors should address and discuss the potential impact of this effect on their results.*

   We thank the reviewer for pointing this out. A similar question was raised by reviewer 2 concerning possible overexpression of the G3AG fusion protein. As shown above by Western Blot analysis (Reviewer 2, point 1), we have carefully selected cells with very low expression of G3AG. In addition, we rarely observed

spontaneously formed SGs in mock samples when we verified MS-data of SG components by IFA in 17clone1 G3AG cells and further never found differences to 17clone1 wildtype cells. The occasional appearance of green-fluorescent foci, particularly at the beginning of the live imaging recording periods, is very likely a result of mild stress of some cells when the culture wells were transported to and mounted at the microscope for imaging. These spontaneously formed foci are usually very weak, and their number decreases with progressing imaging, which differentiates them from SGs formed during stress. These initial green foci are already included in the time-course where SGs had been quantified. As shown in Fig.4B (and the new live-cell imaging data in Fig. 8E/F), the number of those early foci is neglectable.

2. *The image chosen for the mock condition in panel 1D gives the impression that G3BP1 levels are substantially lower under unstressed conditions. However, this appears consistent with the levels observed in panel 1C in 17Cl1 wild-type cells. Can the authors confirm whether this mock control is the appropriate match for comparison?*

   We have carefully checked the identity of the provided figure panels in Figure 1D and Figure 1C and can confirm that they are correct. The low signal for G3BP1 in mock samples (unstressed conditions) is likely due to the fact that G3BP1 is distributed throughout the cytoplasm, while under conditions of stress G3BP1 condenses to well visible puncta (SGs).

3. *The authors use Markmiller et al. (2018) as a reference for identifying SG core and associated proteins, likely due to the shared methodology. However, other APEX-based studies, such as Marmor-Kollet et al. (2020) (PMID: 33217318) and Qin et al. (2023) (PMID: 37385249). All used different cell types and stress conditions. Some proteins categorized here as "non-SG associated," may appear in other studies using similar or different methodological approaches. The authors should clarify that these proteins were not detected in the Markmiller et al. dataset specifically, rather than generally excluding them from SG association. Comparison could also be made with the highest-confidence stress granule proteins listed in the RNA Granule Database (PMID: 31626750 and PMID: 36662637), which likely includes a broader range of candidate proteins.*

   Thanks a lot for this comment. We agree that the use of a reference list based mostly on the findings of Markmiller et al. does not provide a complete picture of the current knowledge on the SG proteome. Thus, as suggested, we repeated the

mass spectrometry data analysis using a new reference list containing the highest-confidence proteins (Tire 1) based on the RNA granule database. All figures and the corresponding figure legends referring to the SG reference list (namely Fig 2, 3 and 5, and supplementary figures 3, 5, 6, 7 and 8) were updated accordingly, and the main text was adjusted.

4. *The authors observed that 144 known SG-associated proteins did not meet significance criteria under arsenite treatment, and 164 SG-associated proteins were not enriched in MHV-infected cells. Several studies have proposed that SG seeds exist under normal conditions and expand into larger assemblies upon stress. This model could explain the lack of significant enrichment observed, which warrants further discussion.*

   In our previous discussion, we just briefly mentioned the “preexisting network” theory. Now we further elaborate on a possible explanation of this theory for the lack of significant enrichment of some SG associated proteins in the arsenite treated or infected samples (lines 539-545; 542-548 in track changes manuscript).

   “A similar observation was made by Markmiller et al. and others suggesting that this could indicate a preexisting network of SG protein interactions in the unstressed cell, which is intensified upon stress (12,13). Such stress-independent interactions between G3BP1 and other SG proteins could further explain that several known SG associated proteins did not show significant enrichment for arsenite treated or infected cells in our proximity labeling experiments, therefore leading to a potential underestimation of the proteins accumulated in SGs.”

5. *Figure 3: While the figure and Venn diagrams are clearly presented and effectively highlight candidate proteins, it is difficult to follow the corresponding categories referenced in the main text. Some categories, such as "stress independent MHV," are unclear. Additionally, viral proteins appear both in the "novel candidates MHV" group and in "MHV enriched (not SG reference)." The authors should clarify these categories and their distinctions to improve clarity.*

   We thank the reviewer for this comment and have adapted the main text to clarify and better explain the different categories. Additionally, we changed the names in Figure 5D/E from “not SG ref” to “novel candidates”, as this group reflects potential new SG protein candidates.

6. *Are the MHV viral proteins found in SGs dispensable for replication?*

Although we have identified the viral proteins nap1, nsp2, nsp4, nsp8, and the structural proteins S, M, and N, we would not claim that these proteins are specifically enriched in SGs. The reason for this is that as a comparator for the analysis we used non-infected cells and can therefore not determine if those proteins would be enriched. Concerning the question of whether these viral proteins are essential for replication, except for nsp2, all other detected proteins are essential (1).

1) *Fehr, A. R. & Perlman, S. Coronaviruses: An Overview of Their Replication and Pathogenesis. in Coronaviruses: Methods and Protocols 1282, 1–23 (2015).*

7. *The comparison of MHV and SFV replication kinetics and ISR activation is insightful, showing that although both viruses follow similar replication dynamics, MHV replicates at lower levels (based on infectious particle release, almost 2 logs difference) and induces less eIF2α phosphorylation (Fig. 4E and 4F). However, the authors do not address the potential relationship between the amount of dsRNA generated during viral replication and PKR activation. Is PKR phosphorylated during these infections? Additionally, is the level of dsRNA lower in MHV-infected cells compared to SFV-infected cells? Such differences could help explain the observed dynamics. Are the numbers of infected cells comparable between the two viruses? Furthermore, how many infected cells exhibit stress granules at various times post-infection (hpi)? Providing this information would strengthen the conclusions and offer a more comprehensive understanding.*

Thanks for the suggestion to further elucidate the relationship between ISR, PKR activation, and the level of dsRNA in our infection model. Concerning the activation of PKR, we were unfortunately unable to identify an antibody that allowed us to measure the level of phosphorylated PKR during SFV and MHV infection. However, we performed live-cell imaging on cells infected with either SFV or MHV and treated with the PKR inhibitor C16 (50uM). The experiment showed a substantial reduction in the SG number for both viruses (see figure below), indicating that PKR seems to be a driver of SG formation during both virus infections. We show here the corresponding data for the attention of the reviewer and would prefer not to include these data in the manuscript since we think they are still preliminary and further, more detailed studies would be needed.

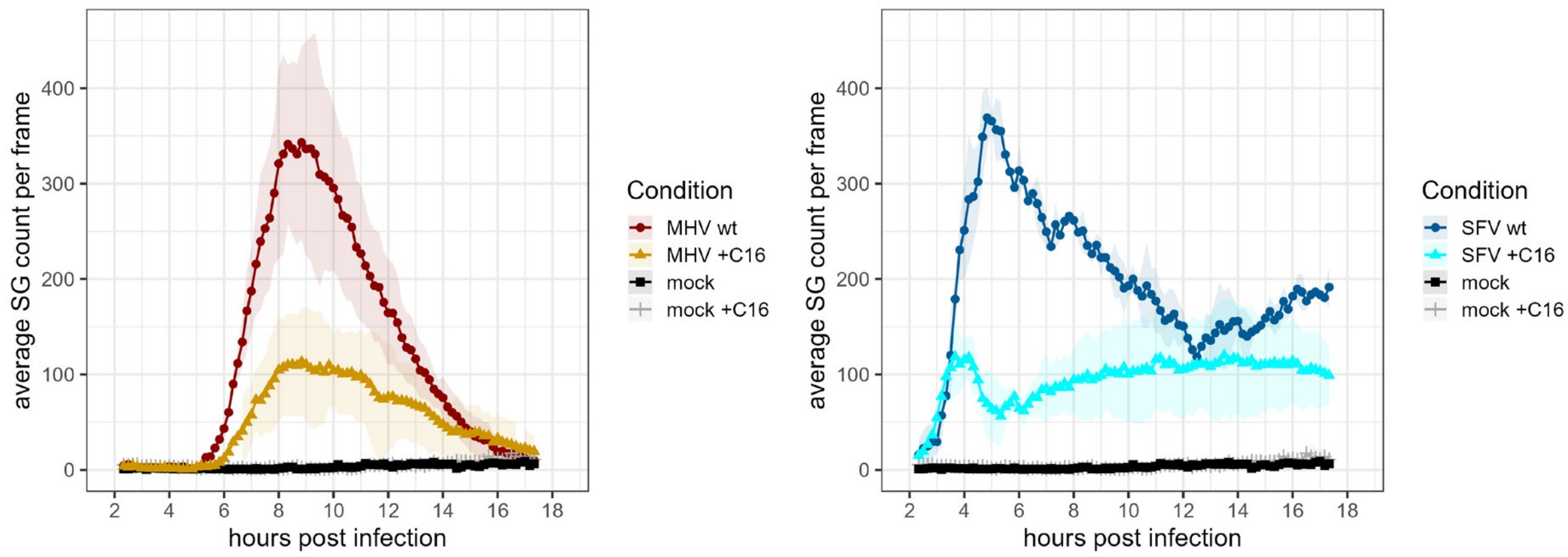

SG formation in MHV- or SFV-infected cells treated with or without PKR inhibitor C16. *17Cl1:G3AG cells were infected with MHV or SFV at an MOI 10. For PKR inhibitor samples, the infection medium and medium added after washing (1.5hpi) were supplemented with PKR inhibitor C16 (1uM). Live-cell imaging was performed up to 17.5hpi. Graphs display the average SG count per frame over time for MHV (left) and SFV (right). SGs per frame were quantified automatically using Ilastik-based segmentation, and the average count was calculated over 2-3 image series per condition.*

We also addressed the question concerning the level of dsRNA in MHV- and SFV-infected cells and observed that indeed the level of dsRNA is higher in SFV-infected cells compared to MHV-infected cells, particularly during the early phase of the infection. This may indeed result in a later appearance of SGs in MHV-infected cells and reflects that coronaviruses have multiple mechanisms to evade detection of dsRNA by Mda5, OAS, and PKR (1,2). We have included these observations concerning kinetics of dsRNA levels in the revised manuscript. The corresponding new figure 8 shows the dsRNA measurement by flowcytometry in MHV or SFV infected cells and additional investigations of dsRNA levels and SG formation during infection with an endonuclease-deficient MHV-nsp15 mutant.

1) *Kindler, E. et al. Early endonuclease-mediated evasion of RNA sensing ensures efficient coronavirus replication. PLoS Pathog. 13, 1–26 (2017).*
2) *Steiner, S. et al. SARS-CoV-2 biology and host interactions. Nat. Rev. Microbiol. 22, 206–225 (2024).*

8. *An intriguing finding is the absence of key translation-related proteins such as eIF3a/c and eIF4GI, major components of the translation machinery. Generally, quantifying the correlation either by Pearson correlation or Manders' coefficient would strengthen the colocalization conclusions. The authors do not discuss potential differences in translation strategies between MHV and SFV. Specifically, does SFV recruit distinct translation initiation complexes for genome translation compared to MHV?*

We thank the reviewer for this comment, and we have now included a detailed quantification of key translation-related proteins. The corresponding results are

displayed in the new Suppl. Fig. 9. We have also included a new section in the discussion concerning potential differences in translation strategies with a focus on initiation of translation (lines 588-598; 593-603 in track changes manuscript).

"As alpha- and coronaviruses possess 5'-cap structures on their genomic and subgenomic mRNAs, it is believed that they require the cap-dependent translation initiation machinery to produce viral proteins (39,40). Interestingly, in the case of alphaviruses, the translation of subgenomic mRNA (sgmRNA) was found to be independent of eIF4F complex and eIF2, whereby the latter is mediated by a downstream stable hairpin structure in the coding region of the sgmRNA (29,41). Nevertheless, there is evidence that sgmRNA translation of alphaviruses requires other factors including eIF3 (42). Coronaviruses on the other hand were found to express nsp1 to profoundly manipulate host translation. For SARS-CoV-2, it was shown that this viral protein directly interacts with the ribosome causing translation inhibition of host transcripts, while promoting translation of viral RNAs (43,44). Which eIFs are required for coronavirus translation is currently unclear."

9. *The co-localization of SFV genomic RNA within SGs is observed transiently, specifically at 5 hours post-infection (hpi) (Fig. 8C). This timing is notable but remains unexplored. Interestingly, this timeframe coincides with the beginning of the exponential phase of viral release. Given that previous studies—and the authors' own discussion—indicate that SGs induced by MHV and SFV do not exhibit an antiviral function, it would be valuable to investigate this further and inhibit SG condensation, for example using commercially available G3BP1 inhibitors such as G3Ia or G3Ib. Assessing how the disruption of SG formation and the consequent absence of SFV genomic RNA localization within SGs affects the viral life cycle could provide important mechanistic insights.*

   We thank the reviewer for this comment and we agree that inhibition of SG formation would provide further insight concerning the question to which extent SGs may exhibit antiviral function. We have performed additional live-cell imaging on cells infected with either SFV or MHV and treated with the G3BP1 inhibitor G3Ia. Notably, we did not detect any increase of viral replication in MHV- or SFV-infected cells that were treated with the PKR inhibitor. This corroborates our hypothesis that SGs are unlikely to exert antiviral activity against MHV. Concerning SFV infections in presence of the PKR inhibitor we hypothesize that nsP3-mediated dissolvement of SGs during the early phase of infection may already be very effective and the G3BP1 inhibitor may not provide an additional advantage for viral replication. We show here the corresponding data for the attention of the reviewer and would prefer not to include these data in the

manuscript since we think they are still preliminary and further, more detailed studies would be needed.

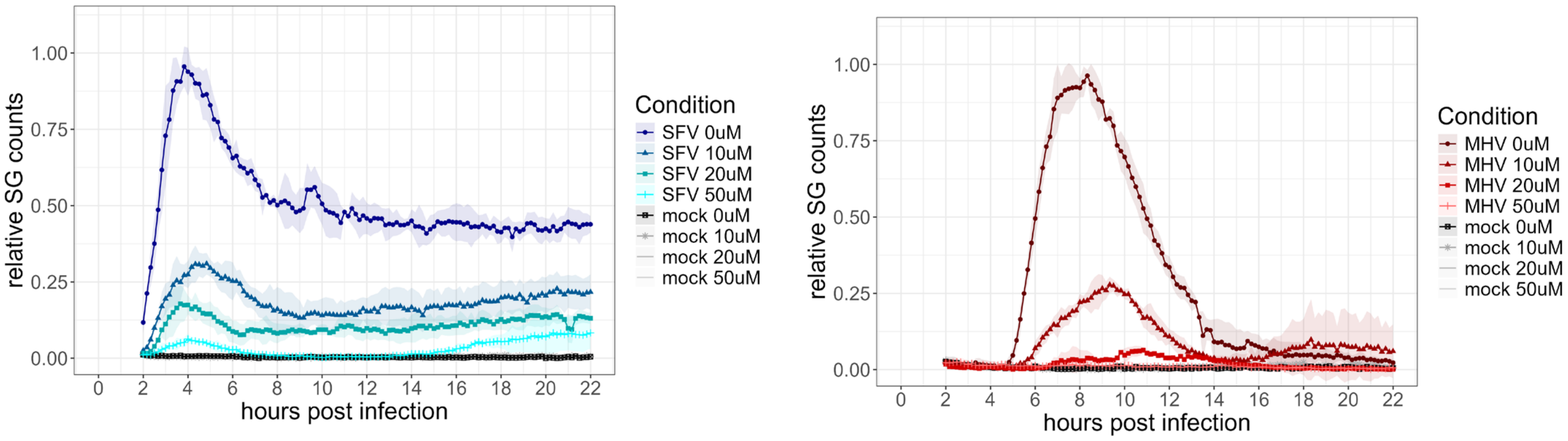

SG formation during MHV and SFV infections with different concentrations of G3BP1 inhibitor G3Ia assessed by live-cell imaging. *17Cl1:G3AG cells were infected with SFV (left) or MHV (right) at an MOI 10. At 1.5hpi, cells were washed and medium supplemented with the indicated concentration of G3BP1 inhibitor G3Ia was added, and cells were imaged up to 22hpi. SGs per frame were counted using an automated image analysis workflow based on Fiji/Ilastik. The graphs show the mean relative SG count per frame (SG count/max. SG count in 0uM samples) over 3 image series per condition. Ribbon indicates standard deviation.*

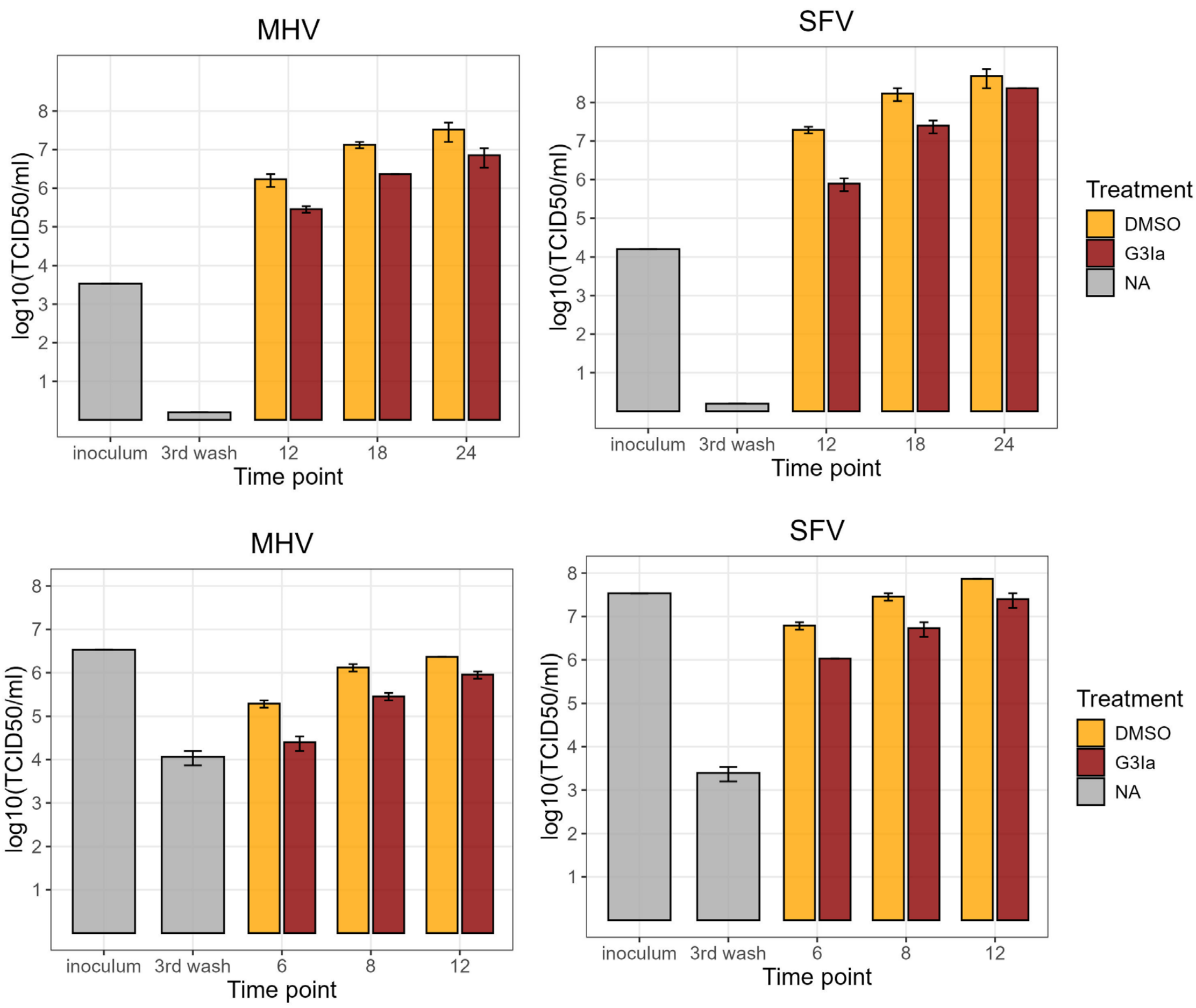

Effect of SG inhibition by G3Ia G3BP1 inhibitor on replication kinetics of MHV (left side) or SFV (right side). *17Cl1:G3AG cells were infected with MHV or SFV at an MOI 0.01 (top row) or 10 (bottom row) to investigate multicycle (top row) or single cycle (bottom row) kinetics. At 1.5hpi, cells were washed three times (third wash collected) and medium supplemented with 50μM G3BP1 inhibitor G3Ia or DMSO was added. Supernatants were collected at indicated time points, and titers were estimated by TCID50 assay. The graphs show the mean titers based on two replicates; the error bars indicate the standard deviation.*

10. *Consistent with this observation, UBAP2L appears to be enriched in SFV-induced stress granules at 5 hours post-infection but is markedly depleted by 9 hours post-infection (Fig. 5B and 5C). This raises the question of whether UBAP2L functions as a host dependency factor or a restriction factor. Silencing UBAP2L could provide valuable insights into the dynamic interplay between stress granule formation and viral replication strategies.*

    We agree that UBAP2L is certainly an interesting target to further investigate possible functions as host dependency factor or restriction factor, especially with its important role in SG formation (1,2). However, we think a detailed analysis of UBAP2L involvement in SFV and MHV infection is beyond the scope of this work. We are, however, very interested in addressing this question in future studies.

1) *Cirillo, L. et al. UBAP2L Forms Distinct Cores that Act in Nucleating Stress Granules Upstream of G3BP1. Curr. Biol. 30, 698-707.e6 (2020).*
2) *Markmiller, S. et al. Context-Dependent and Disease-Specific Diversity in Protein Interactions within Stress Granules. Cell 172, 590-604.e13 (2018).*

11. *This work represents a valuable resource for the community; however, no statement regarding data availability was provided. In the interest of FAIR data principles, the authors should feel encouraged to deposit their mass spectrometry dataset in a public repository such as PRIDE to ensure accessibility and reuse.*

    The raw data is now available on PRIDE under the identifier PXD077432. A statement referring to the repository was further added to the manuscript.

Minor comments

1. *The term SG "disassembly" is more accurate than "dissolvement."*

   To use the more accurate wording, we replaced “dissolvement” with “disassembly” throughout the manuscript.

2. *Line 172: Reference to Supplementary Table S1 is missing.*

   We added the reference to Supplementary Table S1 along with a short description.

3. *SG composition changes over time. The comparison between MHV and SFV may be influenced by differences in SG maturation stages. The authors should cite the work by Hu et al. (2023) (PMID: 38012130), which discusses compositional differences between early and late SGs formed under the same stress conditions.*

   We thank the reviewer for this comment and have expanded our discussion with a sentence specifically highlighting the potential influence of the different maturation stages on SG composition in our experiments and added the reference of Hu et al (lines 611-614; 615-619 in track changes manuscript).

   “Another factor could be the maturation stage of SGs as a previous study showed differences in the SG proteome depending on their maturation stage (46). As we only compare the peak time point for each virus, we cannot exclude that some differences might arise from different stages of SG maturation.”

4. *Figs S10 and S11: show each stain individually for better clarity and the merge.*

   The supplementary figures S10 and S11 were adjusted to display each channel individually.

5. *Fig S12: show the dsRNA stain.*

   Due to an incompatibility of the used antibodies for dsRNA and TAF15, we were unable to do co-staining and thus do not have the dsRNA staining for TAF15.

Reviewer 4:

Lang et al. set out to elucidate the compositional diversity of stress granules (SGs) within different infection context, specifically during MHV and SFV infection. For this they generated a recombinant mouse fibroblast 17C11 cell line stably expressing a G3BP1 fused to APEX2 and eGFP called G3AG. Initially the performed proximity labelling in mock, arsenite and MHV-infected cells. Multiple comparisons revealed an overall decrease of SG proteins and translation factors within MHV-induced granules compared to arsenite-induced SGs. Further comparisons to SFV-induced granules showed that their composition resemble arsenite-induced SGs more than MHV-induced granules. The authors observed that while SFV induces strong phosphorylation of eIF2α, MHV did not, which might relate to the differences seen in SG composition. The authors further compared the G3BP1 microenvironment at different timepoints of SG assembly during viral infection. For SFV-induced SGs they observed high similarities between the G3BP1 microenvironment at 'peak SG' and 'post SG' timepoints. The 'weaker' enrichment of eIFs in MHV granules (as suggested in the proteomics data) is supported by IFA staining for different eIFs. Further, the authors observe a stronger cytoplasmic localisation of hnRNPs to the cytoplasm during SFV infection. Finally, the authors perform smRNA-FISH staining to show differences in viral RNA localisation patterns between MHV and SFV infection.

Overall, the study described a novel cell line G3AG, which could prove useful for further studies focusing on SGs. The authors perform thorough comparisons of SG proteomics induced by chemical stress, MHV and SFV infection. However, they fail to convincingly validate their proteomics data and further don't provide a functional characterisation of the observed SGs. Therefore, the authors are encouraged to provide more experimental data to substantiate the conclusions drawn in this manuscript and further show the value of the presented proteomics data for our understanding of SGs during viral infection.

We thank the reviewer for the comments and have thoroughly addressed the specific comments below to further corroborate our main conclusions.

Major comments

1. Can the authors provide experiments addressing the functional relevance of SGs during viral infection. Does depletion or stabilization of SGs have any effect on viral replication or cell survival?

   We thank the reviewer for this comment and have performed additional experiments using the G3BP1 inhibitor G3Ia. The data is shown above (reviewer 2 point 9).

2. *For the IFA experiment on MHV-infected cells, cells were treated with HR2 peptide to prevent syncytia formation. Can the authors please mention this within the main text and comment if prevention of syncytia formation has any effect on SG formation during MHV infection?*

We thank the reviewer for this comment and mention the use of the HR2 peptide now also in the main text (lines 127-129, "For infections with MHV, we supplemented the medium with HR2 peptide. This prevents syncytia formation and allows analysis of single cells, especially important for imaging."). To address the question of whether prevention of syncytia formation impacts SG formation, we have directly compared SG formation in MHV-infected cells that were treated or not treated with the HR2 peptide. As shown here for the reviewer's attention, we did not observe any impact of the HR2 peptide on SG formation.

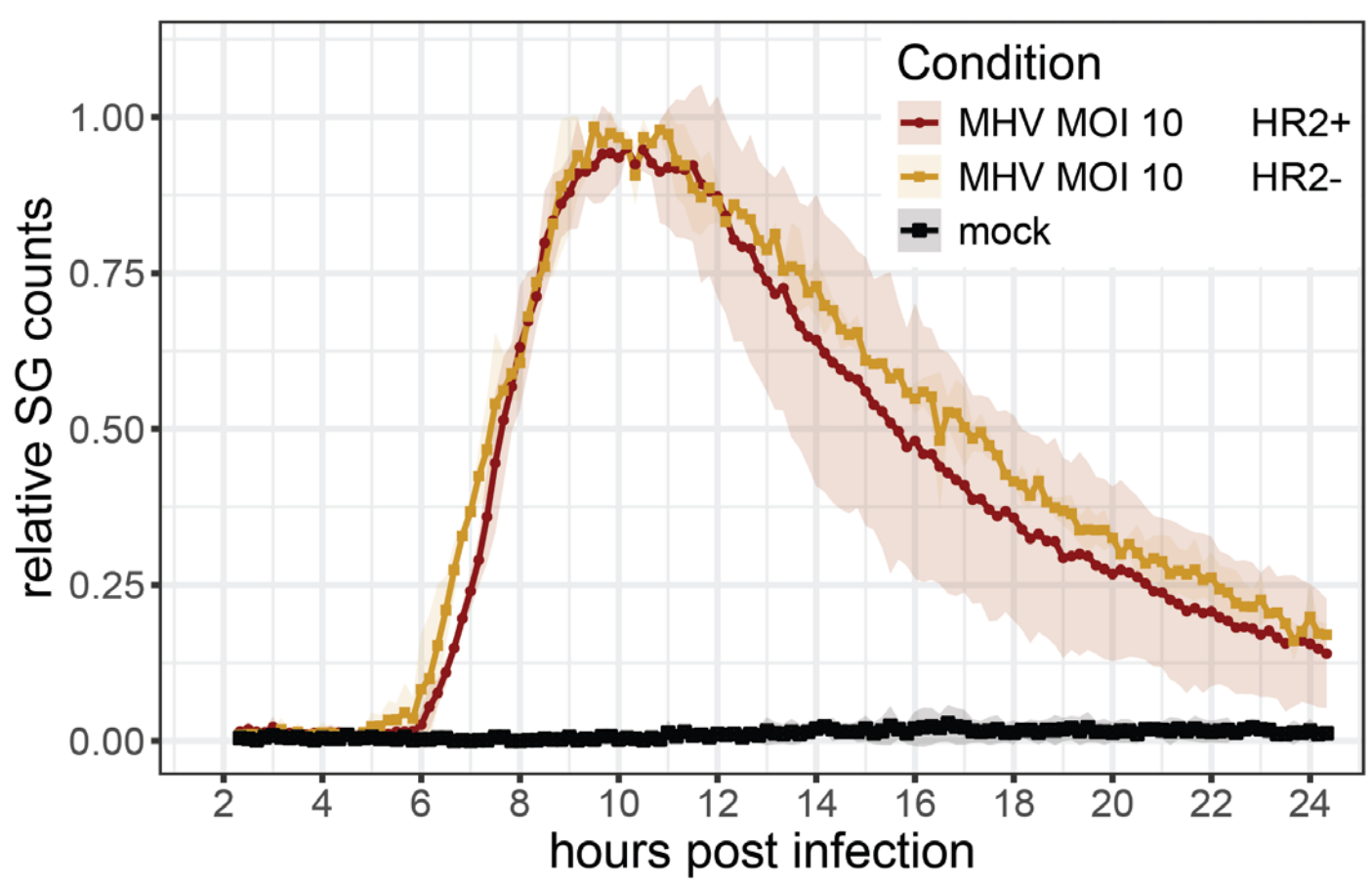

SG formation during MHV infection with and without HR2 peptide assessed by live-cell imaging. *17Cl1:G3AG cells were infected with MHV at an MOI 10 or mock infected. At 1.5hpi the infection medium was exchanged with medium supplemented with HR2 peptide (HR2+) to prevent syncytia formation, and cells were imaged up to 24hpi. The graph shows the relative SG count per frame (SG count/max. SG count for corresponding image series) summarized over 3 image series for each condition to visualize the SG formation dynamics. The ribbon indicates the standard deviation.*

3. Re Fig 2 B: the authors suggest that MHV induces a weaker stress response than arsenite leading to less accumulation of SG proteins and translation factors to MHV-induced granules. For SARS-CoV2 it is known that it induces a strong translation shut-off on infected cells via the action of Nsp1.

    a. Are some host cell proteins at 8hpi in MHV-infected cells less abundant leading to lower enrichment during down-stream processing?

We cannot exclude that some host cell proteins may be less abundant at 8hpi in MHV-infected cells. Proteins that we have analyzed, such as G3BP1 (see below) do not show any difference concerning the overall amount of protein in the cytoplasm. We would rather argue that most translation-related proteins are heavily involved in translating the abundant viral mRNAs and would probably be therefore reduced in SGs at this time point. We have included this hypothesis in the discussion (line 607-6011; 612-15 in track changes manuscript).

"On the other side, the efficient translation of viral RNA at later time points might occupy a large fraction of eIFs preventing their accumulation in SGs. This could also lead to smaller granules and would further explain why the major SG nucleator still seem to accumulate in comparable levels for canonical and atypical SGs."

b. Can the authors provide a western blot of major SG proteins of mock, arsenite treated cells, MHV (8hpi) and SFV (5hpi).

Early in the project, we performed a Western blot analysis to investigate the protein level of some SG proteins at different time points of SFV and MHV infections (pre SG and peak SG time points for each) and could not observe substantial differences between the viruses at the peak time points. Below, the Western Blot analysis of G3BP1 is shown as an example.

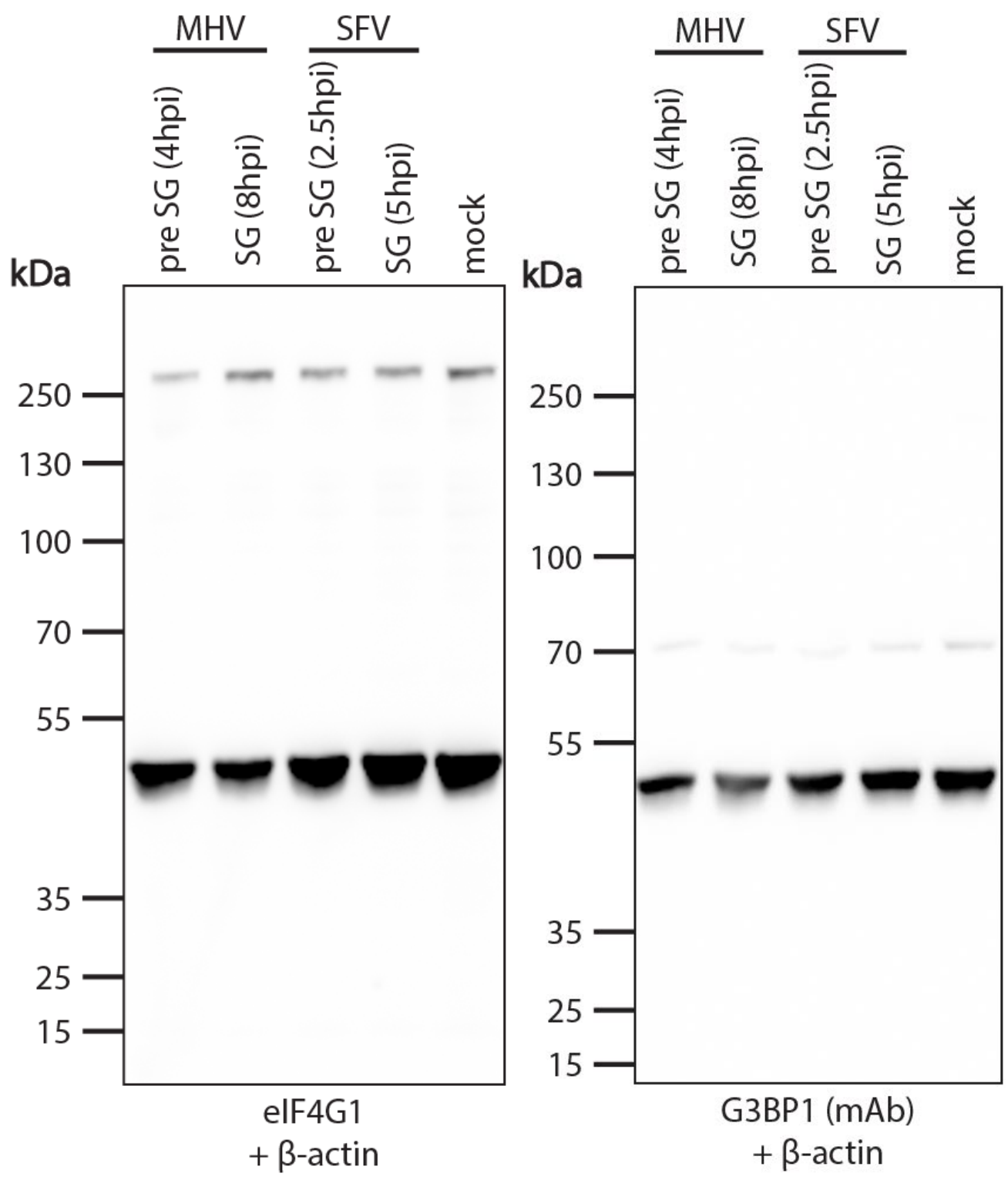

Assessing protein levels of eIF4G1 and G3BP1 at different time points of MHV and SFV infections. *17Cl1:G3AG cells were infected with MHV or SFV at an MOI 10 or mock infected. Lysates were collected at indicated time points (mock at 5hpi) and subjected to Western blot analysis targeting eIF4G1 (left) or G3BP1 (right). Staining for β-actin was used as loading control.*

c. Alternatively, it might be that the granules differ in their fluidity and therefore exchange rate with the cytoplasmic environment. Can the author provide FRAP experiments addressing the biophysical properties of the observed granules?

We agree with the reviewer that the SG fluidity and exchange rate with the cytoplasmic environment would be interesting to address in order to further dissect possible SG functions during infection and possible viral interference with SG formation and function. We think that although such studies may provide valuable clues concerning differences of SGs, they would at this point still be preliminary. The question of whether there are biophysical differences would require extensive additional studies that we think would go beyond the scope of this work.

d. Can the authors provide quantification of the SG size in MHV-infected cells compared to SFV-infected and arsenite-treated cells?

We thank the reviewer for this comment and have compared the SG size in MHV-infected cells, SFV-infected cells, and sodium arsenite-treated cells. The results are shown in the new figure panel Fig.7H.

4. Re Fig 3 and 5: Could the authors provide and discuss comparisons of the here generated proteomics data for arsenite, MHV- and SFV-induced granules with previously published proteomics data for SGs, paracrine granules and P-bodies?

   This comment also relates to the comment of reviewer 3 (point 3) concerning the reference list of SG proteins. We have carefully adapted the reference list (as mentioned above). We have also included a brief discussion of paracrine granules and P-bodies in the discussion section (lines 577-585; 581-589 in track changes manuscript).

   "In contrast, other condensates like processing bodies (PBs) or the recently described paracrine granules formed in uninfected bystander cells during infection, are characterized by the absence of many eIFs (2,38). However, our identified atypical SGs induced by MHV do seem to be distinct from both. PBs are enriched in proteins involved in mRNA decay like the decapping enzyme DCP1A, which we did not observe for MHV-induced SGs (2,13). The paracrine granules on the other hand are characterized by the accumulation of large ribosomal proteins and their resistance to disassembly by cycloheximide, which are distinct from the atypical SGs described here (38). Furthermore, as we used a high MOI for our experiments, the fraction of uninfected bystander cells is minimal."

5. Fig 3 B: Can the authors show that selected MHV-induced SG interactors are indeed localizing to SGs and do these interactors have anti- or pro-viral properties?

   We are not entirely sure which MHV-induced SG interactors are meant here. We have provided in Fig. 7 and Suppl. Fig. 10 IFAs of several SG interactors and show their localization in relation to G3BP1 and dsRNA.

6. *Fig 3B: can the authors speculate why in MHV-induced SGs they find SG nucleators (Caprin1) and at the same time disassembly factors (USP10), as these are competing for G3BP1 interaction.*

   Although the mentioned factors were shown to bind G3BP1 in a mutually exclusive manner, on the level of SGs it is possible to find both factors, which was

also shown in previous studies (1,2,3). The presence of both factors in MHV-induced SGs could be a result of the stage of SG maturation.

1) *Markmiller, S. et al. Context-Dependent and Disease-Specific Diversity in Protein Interactions within Stress Granules. Cell 172, 590-604.e13 (2018).*
2) *Youn, J.-Y. et al. High-Density Proximity Mapping Reveals the Subcellular Organization of mRNA-Associated Granules and Bodies. Mol. Cell 69, 517-532.e11 (2018).*
3) *Vu, L. et al. Defining the Caprin-1 Interactome in Unstressed and Stressed Conditions. J. Proteome Res. 20, 3165–3178 (2021).*

7. *Fig 3 C: does any of the MHV proteins localize to SGs as suggested by the proteomics data? Can the authors provide IFA experiments?*

Thank you for the question. We did an IFA experiment in MHV-infected cells with an antibody available in our lab targeting the nucleocapsid protein N. As shown below, the N protein seems to localize to SGs and appears to be slightly accumulated in these condensates. However, the accumulation is not as strong as observed for other proteins (e.g. SG marker TIA1). It is important to mention that in our proteomic screen we compare conditions with virus infection against conditions without. Thus, a significant enrichment of viral proteins when comparing MHV vs. Arsenite or mock does not necessarily prove the accumulation of these proteins in SGs.

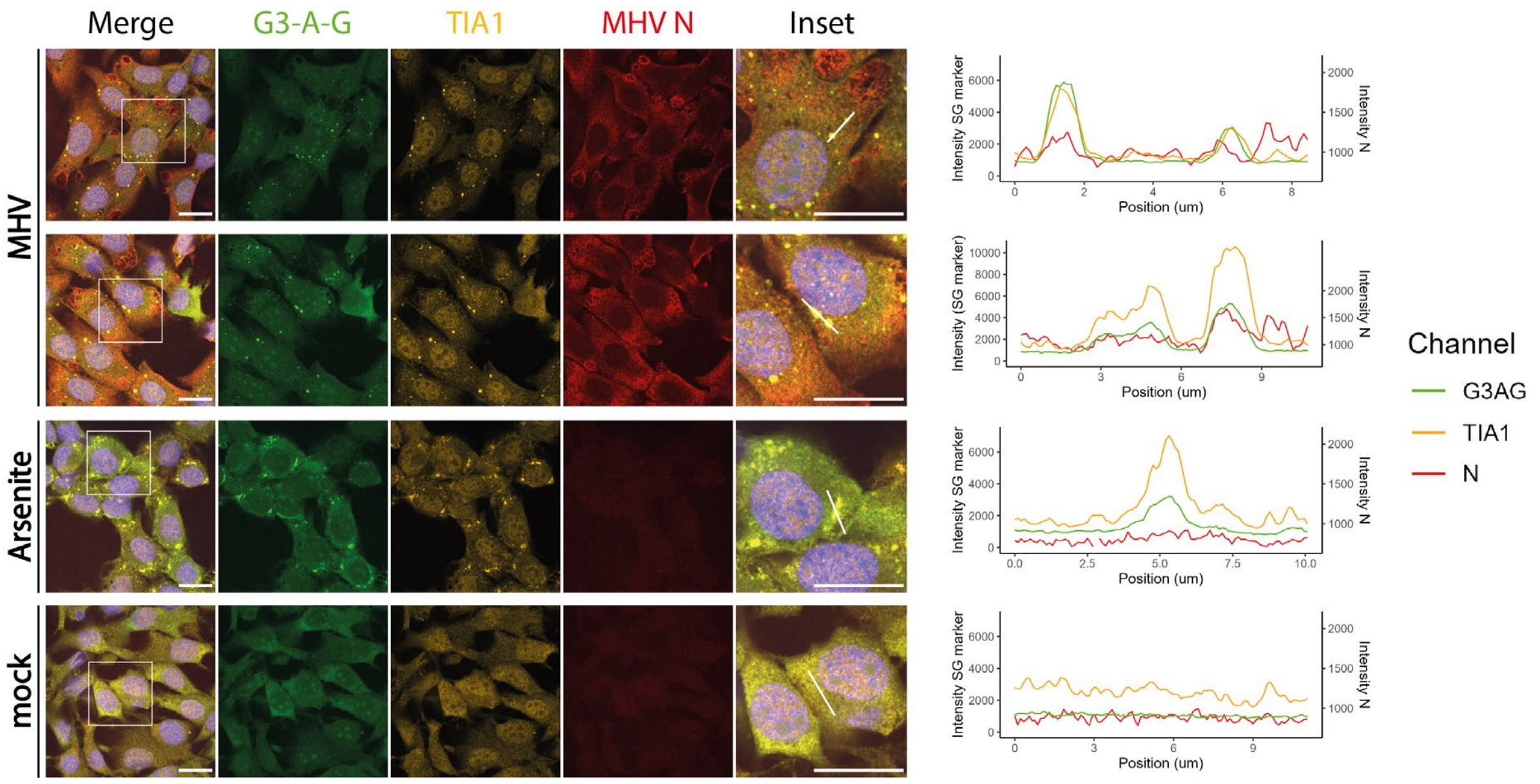

Investigating the subcellular localization of nucleocapsid protein (N) of MHV in relation to stress granules. *17Cl1 cells stably expressing a G3BP1-APEX2-GFP fusion protein (green) were infected with MHV at an MOI 10 or treated with 250uM sodium arsenite. The cells were fixed at 8hpi or 30min after treatment respectively and stained with antibodies targeting TIA1 (orange) or MHV N (red). Nuclei were stained with DAPI (blue). Scale bar: 20µm. The intensity profile of each channel (except DAPI) was measured along the indicated lines and plotted.*

8. Fig 4 B: The authors speculate that SG disassembly during SFV depends on the action of the viral nsP3.

   NsP3 mediated SG disassembly during SFV and other alphavirus infection is based on previous studies reporting a role of the alphavirus nsP3 in SG disassembly or inhibition by sequestering G3BP1 (1,2) or counteracting ADP-ribosylations (3). However, the importance of alphavirus nsP3 in SG disassembly is still disputed, and some studies suggest that the main driver of SG disassembly is the virus-induced transcriptional and translational shut down rather than the nsP3-G3BP interaction (4).

   a. Can the authors provide a WB showing that nsP3 expression co-occurs with SG disassembly?

      As mentioned above (reviewer 1, point 3) we did not focus on a closer investigation of the SFV nsP3-G3BP1 interaction since this already has been addressed in several previous studies (1-4). It is known that nsP3 expression is starting to increase at the early time of infection when SGs are disassembled.

   b. Further, potentially the disassembly observed during MHV and SFV-infection occurs as a by-production of the activation of intrinsic cell death pathways. Can the authors comment on when cells become apoptotic upon viral infection. Do the authors still observe SG disassembly if cells are treated with caspase inhibitors?

      During the early phase of virus infection (up to 5hpi) it is highly unlikely that infected cells are already apoptotic. For SFV it was found that cell death only starts after 10hpi (5). We therefore expect that the disassembly of SFV-induced SGs is independent of apoptosis. At a later phase of infection when MHV-induced SGs appear (8-12hpi) the cells are likely apoptotic, but we have not investigated if this may impact the disassembly of SGs.

1) *Panas, M. D. et al. Sequestration of G3BP coupled with efficient translation inhibits stress granules in Semliki Forest virus infection. Mol. Biol. Cell 23, 4701–4712 (2012).*
2) *Kedersha, N. et al. G3BP–Caprin1–USP10 complexes mediate stress granule condensation and associate with 40S subunits. J. Cell Biol. 212, 845–860 (2016).*
3) *Jayabalan, A. K. et al. Stress granule formation, disassembly, and composition are regulated by alphavirus ADP-ribosylhydrolase activity. Proc. Natl. Acad. Sci. 118, (2021).*

4) *Frolova, E. I., Palchevska, O., Dominguez, F. & Frolov, I. Alphavirus-induced transcriptional and translational shutoffs play major roles in blocking the formation of stress granules. J. Virol. 97, (2023).*
5) *Jenster, L.-M. et al. P38 kinases mediate NLRP1 inflammasome activation after ribotoxic stress response and virus infection. J. Exp. Med. 220, (2023).*

9. *Fig 4 D: Can the authors provide a quantification of the percentage of cells displaying SGs?*

   We thank the reviewer for this comment, and we have now provided the quantification of the SG number per cell (Fig.4e).

   a. Can the authors comment what effect 1.5h cycloheximide treatment has on a viral infection that is 5 or 8 hours long respectively.

      Because viruses, including MHV and SFV, rely on the cellular translation machinery, the inhibition of translation does negatively affect the production of viral proteins and with it the viral growth. Such an effect we can see in our data for SFV, where we observed a reduced number of infected cells for cycloheximide treated samples. However, despite the lower number of infected cells, we observe a reduction of SGs in infected cells, which is in line with cycloheximide-induced SG disassembly.

   b. For MHV the cell seems to be less infected with might be the actual reason that no SGs are formed. Can the authors provide an alternative experiment to prove that the observed virus-induced SGs resemble canonical SGs?

      The observation that cycloheximide treatment dissolves the G3BP1-postive granules in MHV-infected cells suggests that these granules are SG. However, our detailed analyses of the SG composition support the idea that the MHV-induced granules are similar to canonical SGs, but somewhat “atypical”. We have discussed this extensively in lines 554-561/568-585 (557-565/572-589 in track changes manuscript) in the discussion.
      As an alternative experiment to assess if the MHV-induced granules resemble SGs, we treated MHV-infected cells with the G3BP1 inhibitor G3Ia which is known to inhibit SG formation. The data are shown above in response to point 9 of reviewer 3 and demonstrate that G3Ia treatment results in inhibition of SG formation in SFV- and MHV-infected cells. We

therefore conclude that MHV-induced granules share with canonical SGs also the inhibition of granule formation with the G3BP1 inhibitor G3Ia.

10. *Fig. 4 E and F: There is no description provided in the figure legends for panel E and F. Further, can the authors add statistics to panel F and adjust y-axis labelling to reflect that panel F refers to eIF2α phosphorylation.*

The missing information on figure 4 F and G (formerly E, F) was added to the figure legend. Furthermore, we performed statistics on the results shown in panel G (Western blot) and adjusted the label of the y-axis to increase clarity.

11. *Fig 4 E: The authors observe lower phosphorylation of eIF2α in MHV-infected cells compared to SFV-infected and arsenite-treated cells.*
    a. *Does this correlate with activation of PKR and subsequent translation shut-off? If translational activity is retained in MHV-infected cells, that might explain why eIFs are less enriched in MHV-induced granules compared to arsenite or SFV-induced granules, as further observed in figure 7 A-D.*
    b. *Does MHV potentially induce SG formation via eIF2α- independent pathways? If yes, the differences observed in SG composition might reflect general differences of SGs induced by eIF2α-dependent vs. independent pathways instead of virus dependent differences.*

This comment relates to comment of reviewer 3 (point 7). As mentioned above, we could not measure the level of phosphorylated PKR due to lack of suitable antibodies. However, we previously performed a live-cell imaging of SFV-and MHV-infected cells treated with PKR inhibitor C16 showing a strong reduction in SG numbers for both viruses. These indicate the involvement of PKR and the ISR in SG formation during MHV infection. The reduction of eIFs in MHV-induced atypical SGs could indeed be caused by an efficient translation of viral proteins late in infection, and we now addressed this in the discussion (line 603-607).

"On the other side, the efficient translation of viral RNA at later time points might occupy a large fraction of eIFs preventing their accumulation in SGs. This could also lead to smaller granules and would further explain why the major SG nucleator still seem to accumulate in comparable levels for canonical and atypical SGs."

Concerning an eIF2a-independent SG induction, we cannot currently rule out the activation of such pathways during MHV infection. However, a previous study on the stress induction during MHV infection and our own results showing increased

phoshp-eIF2a at later time points correlating with SG formation strongly suggests that ISR is a major driver of SG formation during MHV infection (1).

1) *Raaben M, Groot Koerkamp MJA, Rottier PJM, de Haan CAM. Mouse hepatitis coronavirus replication induces host translational shutoff and mRNA decay, with concomitant formation of stress granules and processing bodies. Cell Microbiol. 2007;9(9):2218–29.*

In addition, as requested by reviewer 3 (point 7) we assessed the level of dsRNA in SFV- and MHV-infected cells. These analyses are now shown in the new figure 8 (see further comments under reviewer 3, point 7).

12. *Re Fig. 6 A-C: The top 5 enriched pathways are shown for MHV and SFV peak and post SGs. However, no enriched pathway is shown for MHV peak SGs. The authors mention in line 354-356 that due to the only few enriched proteins for SG peak and SG post time points, the meaningfulness of this analysis is limited. However, if that is the case, can the authors please clarify the difference to the MHV enriched pathways depicted in figure 2 D and the MHV peak SGs pathways in figure 6 A-C.*

    The main difference is the condition to which the proteome of MHV-induced SGs is compared to. In figure 2D, the SG proteome induced by MHV infection is compared to either the G3BP1 microenvironment in the unstressed state (mock) or the canonical SG proteome induced by sodium arsenite treatment. In figure 6, we directly compare the proteome between SFV- and MHV-induced SGs. While our screen identified several significantly enriched proteins for MHV-induced SGs when compared to the unstressed cells or arsenite-induced SGs, only few proteins were found significant when compared to SFV-induced SGs reducing the meaningfulness of the corresponding GO term enrichment analysis.

13. Fig 7 E-G, S10-12: The authors found FUS, PTBP1 and TAF15 enriched in SFV-induced SGs and conclude that this is based on the relocalisation of these proteins to the cytoplasm which is different from the predominant nuclear signal in MHV-infected and arsenite-infected cells. However, this relocalisation only becomes significant at 7hpi representing a late timepoint in SFV-infection, where SGs already start to disassemble. For MHV the authors only tested one timepoint (assumingly 8 hpi, please provide information in the figure legend). Could the authors provide quantitative data on the FUS, PTBP1 and TAF15 localisation during late timepoints of MHV infection to provide stronger evidence that this relocalisation is truly virus-dependent and not due to cell damage?

    The detection of FUS, PTBP1, and TAF15 in SFV-induced SGs was indeed surprising, and we therefore addressed if these proteins indeed re-localize from

the nucleus to the cytoplasm/SGs. For MHV, the 8hpi time point is already in the late phase of the life cycle. We assume that FUS, PTBP1, and TAF15 display nuclear localization before the infection and likely also before the formation of SGs, since we did not detect any considerable amounts of these proteins in the cytoplasm or MHV-induced SGs at 8hpi. We therefore assume that these proteins do not change their localization during MHV infection.

14. Fig. 8 C: The authors mention in the discussion, in line 502-503 that 'SFV shows a strong accumulation of gRNA in these granules', although in Fig. 8 C it is demonstrated, that the viral gRNA is located at the periphery of these granules. Can the authors speculate why the polyadenylated SFV gRNA localises to stress granules differently than polyadenylated cellular RNA and if this has implication for viral infection?

    We also noticed that the SFV gRNA is located more at the periphery of SFV-induced SGs. This region is the more dynamic shell of SGs and to some extent in equilibrium with the cytosol. We have not addressed if this may have any implication for viral infection, since this would require extensive additional experiments that we think would go beyond the scope of this study.

15. Within the discussion the authors should speculate on the function of SGs during SFV and MHV infection based on the pathways they identified and on previous research done in viral infections in cells depleted for SG proteins.

    We have extended the discussion on translation-related pathways with a focus on virus-specific interactions with the translation machinery (viral cap structures, sgRNA structural features, coronavirus nsp1 function). We feel that a too broad discussion of pathways and their potential impact on virus infection may dilute the may conclusions of our study.

16. *Could the authors speculate why they fail to observe enrichment of pattern recognition receptors in their granules? Could the choice of cell line used for infection have an influence on the activation of innate immune signalling pathways and therefore localisation of pattern recognition receptors to SGs? Could the authors speculate on possible limitations of the study as using the G3AG cells what is probed is the microenvironment of G3BP1 which might differ to a certain degree from the microenvironment of SGs.*

    The used 17Cl1 mouse fibroblast cell line has long been used in MHV infection studies. It is IFN competent and contains functional RIG-I, PKR and OAS. We therefore assume that the presence or absence of these innate immune sensors

may differ depending on which virus is studied. We have included this notion now into the discussion (lines 637-641; 642-646 in track changes manuscript)

"Importantly, the used 17Cl1 cell line has long been used for MHV infection studies, also because it is fully IFN competent and contains functional RIG-I, PKR and OAS. Thus, the absence of these innate immune sensors in SGs in our study suggest that their abundance in SGs likely differs between viruses, even from the same family, and does not seem to be a general feature of virus-induced SGs."

Minor comments

1. *Re Fig. 8: The authors use an MOI of 0.1 instead of 10, which was used in most of the other experiments. Can the authors please comment on the reasoning of this decision and if a lower MOI does affect the kinetics of SG assembly.*

   The initial decision to use a lower MOI was made to reduce the overall amount of vRNA and thereby get a cleaner staining and potentially visualize single RNA molecules. On the level of individual cells, the lower MOI does not change the SG formation kinetics. In additional experiments verifying the differences in vRNA accumulation in SGs between SFV- and MHV-cells, we used MOI 10 and MOI 0.1 side-by-side and did not observe any notable differences on the level of the individual cells (supplementary figure S16).

2. *Fig. 2, panel A-C: use same cell labelling as in Sppl. Fig. 2 to reflect that all presented conditions underwent APEX2-mediated proximity labelling (e.g. Fig. 2A displays mock APX+ vs. Arsenite, change to mock APEX+ vs. Arsenite APEX+)*

   We adjusted the labels accordingly.

3. *Re Fig. 8: provide probe sequences used*

   The sequences of the used FISH probes are added in a separate excel file as supplementary data **S5 Table** and referenced in the method section.

4. *Some of the reference numbers cited in the text have different font styles (see line 484, 487, 618, 709)*

   We unified the font style of all references.

5. *Material and Methods- Cell culture: provide culture conditions for BHK21 cells (which are used in line 568); Adjust line spacing of that paragraph to fit the rest of the text.*

   We specified the culture conditions for BHK21 cells in the method section (Line 725; 737 in track changes manuscript).

6. *Line 590: provide a short description of the TCID50 assay*

   We added a short description of the TCID50 assay in the method paragraph describing the Single-cycle kinetics (Line 747-750; 795-762 in track changes manuscript).

   "In short, 17Cl1 cells were infected with a tenfold serial dilution of the supernatants (6 columns of 96-well plate pr sample) and incubated for 3 days at 37°C. The cells were fixed and stained with crystal violet. Based on the highest dilutions displaying dead cells, TCID50 was calculated using the Spearman and Kärber algorithm (50)."

7. *Line 601: please clarify if for the arsenite control (treatment 30 min) cycloheximide (treatment for 1.5h) was added before stress granule induction with arsenite.*

   Thanks for pointing out the needed clarification. The cycloheximide treatment was started 1h before treatment of cells with sodium arsenite. Fresh medium supplemented with sodium arsenite and cycloheximide was added 30min before fixation of cells. We added a sentence to the methods clarifying the procedure (Line 761-763; 773-775 in track changes manuscript).

   "For the sodium arsenite treatment, fresh medium supplemented with sodium arsenite and cycloheximide was added 1h after start of cycloheximide treatment and 30min before fixation."

8. *Line 603: missing 'C' for '..PBS at 4° until....;*

   We added the missing "C".

9. *646-648; 667-675: keep spacing between number and unit consistent*
   Corrected

10. *Line 693: add spacing between brackets*
    Corrected

11. *Line 695-703: text is aligned left, make consistent with rest of the text*

    Corrected

12. *Line 721: spelling error: 'imputed'*

    “Imputed” is correctly written, as here we refer to the statistical process to replace missing values in a data set (imputation).

13. *Line 736: spelling error: 'lates'*

    Corrected
