# Supplementary material for: Stress granules formed during infections with RNA viruses have significant virus-specific differences in generation and composition
Source: PLoS Biol. 2026 Aug 14;24(8):e3003363. doi: 10.1371/journal.pbio.3003363 (PMC13497286; doi:10.1371/journal.pbio.3003363)
Supplement: Peer review history

# Editor report: Decision Letter 0

**DOI:** 10.1371/journal.pbio.3003363.r001

**Authors:** Melissa Vazquez Hernandez

10 Aug 2025

Dear Voker,

Thank you for submitting your manuscript entitled "Stress granules formed during different RNA virus infections show remarkable plasticity and substantial virus-specific differences in their formation and composition" for consideration as a Research Article by PLOS Biology.

Your manuscript has now been evaluated by the PLOS Biology editorial staff, as well as by an academic editor with relevant expertise, and I am writing to let you know that we would like to send your submission out for external peer review.

However, before we can send your manuscript to reviewers, we need you to complete your submission by providing the metadata that is required for full assessment. To this end, please login to Editorial Manager where you will find the paper in the 'Submissions Needing Revisions' folder on your homepage. Please click 'Revise Submission' from the Action Links and complete all additional questions in the submission questionnaire.

Once your full submission is complete, your paper will undergo a series of checks in preparation for peer review. After your manuscript has passed the checks it will be sent out for review. To provide the metadata for your submission, please Login to Editorial Manager (https://www.editorialmanager.com/pbiology) within two working days, i.e. by Aug 12 2025 11:59PM.

If your manuscript has been previously peer-reviewed at another journal, PLOS Biology is willing to work with those reviews in order to avoid re-starting the process. Submission of the previous reviews is entirely optional and our ability to use them effectively will depend on the willingness of the previous journal to confirm the content of the reports and share the reviewer identities. Please note that we reserve the right to invite additional reviewers if we consider that additional/independent reviewers are needed, although we aim to avoid this as far as possible. In our experience, working with previous reviews does save time.

If you would like us to consider previous reviewer reports, please edit your cover letter to let us know and include the name of the journal where the work was previously considered and the manuscript ID it was given. In addition, please upload a response to the reviews as a 'Prior Peer Review' file type, which should include the reports in full and a point-by-point reply detailing how you have or plan to address the reviewers' concerns.

During the process of completing your manuscript submission, you will be invited to opt-in to posting your pre-review manuscript as a bioRxiv preprint. Visit http://journals.plos.org/plosbiology/s/preprints for full details. If you consent to posting your current manuscript as a preprint, please upload a single Preprint PDF.

Feel free to email us at plosbiology@plos.org if you have any queries relating to your submission.

Best wishes,

Melissa

Melissa Vazquez Hernandez, Ph.D.

Associate Editor

PLOS Biology

mvazquezhernandez@plos.org

# Peer review: Decision Letter 1

**DOI:** 10.1371/journal.pbio.3003363.r002

**Authors:** Melissa Vazquez Hernandez

13 Sep 2025

Dear Volker,

I hope you are doing great. Thank you for your patience while your manuscript "Stress granules formed during different RNA virus infections show remarkable plasticity and substantial virus-specific differences in their formation and composition" was peer-reviewed at PLOS Biology. Your manuscript has been evaluated by the PLOS Biology editors, an Academic Editor with relevant expertise, and by four independent reviewers.

As you will see in the reviewer reports, all the reviewers are really positive about your study but they share similar concerns regarding the lack of experimental and functinal validation. Reviewer 1 requests validation that MHV truly suppresses SGs early in infection, direct evidence that MHV RNA is excluded from SGs (via co-localization experiments), functional tests confirming that SFV’s NSP3-G3BP1 interaction restricts viral replication, and a clearer discussion of whether MHV-induced structures are genuine SGs or distinct condensates. Reviewer 2 would like you to clarify whether the G3BP1–APEX–GFP fusion is expressed at physiological levels compared to endogenous G3BP1 (since overexpression could alter SG properties) and explain whether the identified glycosylation-related proteins are specific to cytoplasmic O-GlcNAcylation rather than secretory pathways. Reviewer 3 raises multiple concerns or things to be clarified such as the expression level of the G3BP1–APEX–GFP fusion versus endogenous G3BP1 to rule out overexpression artifacts, the role of glycosylation-related proteins, the lack of enrichment of many known SG proteins under arsenite or MHV, the relationship between dsRNA levels, PKR activation, and replication kinetics, and recommends functional tests (e.g., G3BP1 inhibition or depletion of UBAP2L) to assess whether viral protein-SG interactions affect replication. Reviewer 4 has important concerns regarding the lack of validation and further functional characterisation of the observed SGs. The reviewer requests to test the functional relevance of virus-induced SGs, clarify whether HR2 peptide treatment influences MHV-induced SGs, and provide additional validation such as western blots of SG proteins, FRAP analyses, and SG size quantification. S/He also asks for clarification of proteomics categories, localization of viral and host proteins, exploration of differences in dsRNA levels and PKR activation between viruses, and discussion of whether observed SG composition differences reflect virus-specific strategies or eIF2α-dependent versus -independent mechanisms.

IMPORTANT: after discussion with the Academic Editor and the reviewers, while we do not require that you do every single new experiment, it is important to address the share concerns, namely the experimental validation and functional characterisation.

In addition to these revisions, you will need to complete some formatting changes, which you will receive in a follow up email. A member of our team will be in touch with a set of requests shortly.

We appreciate that these requests represent a great deal of extra work, and we are willing to relax our standard revision time to allow you 6 months to revise your study. Please email us (plosbiology@plos.org) if you have any questions or concerns, or envision needing a (short) extension.

At this stage, your manuscript remains formally under active consideration at our journal; please notify us by email if you do not intend to submit a revision so that we may withdraw it.

**IMPORTANT - SUBMITTING YOUR REVISION**

Your revisions should address the specific points made by each reviewer. Please submit the following files along with your revised manuscript:

1. A 'Response to Reviewers' file - this should detail your responses to the editorial requests, present a point-by-point response to all of the reviewers' comments, and indicate the changes made to the manuscript.

*NOTE: In your point by point response to the reviewers, please provide the full context of each review. Do not selectively quote paragraphs or sentences to reply to. The entire set of reviewer comments should be present in full and each specific point should be responded to individually, point by point.

You should also cite any additional relevant literature that has been published since the original submission and mention any additional citations in your response.

2. In addition to a clean copy of the manuscript, please also upload a 'track-changes' version of your manuscript that specifies the edits made. This should be uploaded as a "Revised Article with Changes Highlighted " file type.

*Resubmission Checklist*

When you are ready to resubmit your revised manuscript, please refer to this resubmission checklist: https://plos.io/Biology_Checklist

To submit a revised version of your manuscript, please go to https://www.editorialmanager.com/pbiology/ and log in as an Author. Click the link labelled 'Submissions Needing Revision' where you will find your submission record.

Please make sure to read the following important policies and guidelines while preparing your revision:

*Published Peer Review*

Please note while forming your response, if your article is accepted, you may have the opportunity to make the peer review history publicly available. The record will include editor decision letters (with reviews) and your responses to reviewer comments. If eligible, we will contact you to opt in or out. Please see here for more details:

https://blogs.plos.org/plos/2019/05/plos-journals-now-open-for-published-peer-review/

*PLOS Data Policy*

Please note that as a condition of publication PLOS' data policy (http://journals.plos.org/plosbiology/s/data-availability) requires that you make available all data used to draw the conclusions arrived at in your manuscript. If you have not already done so, you must include any data used in your manuscript either in appropriate repositories, within the body of the manuscript, or as supporting information (N.B. this includes any numerical values that were used to generate graphs, histograms etc.). For an example see here: http://www.plosbiology.org/article/info%3Adoi%2F10.1371%2Fjournal.pbio.1001908#s5

*Blot and Gel Data Policy*

We require the original, uncropped and minimally adjusted images supporting all blot and gel results reported in an article's figures or Supporting Information files. We will require these files before a manuscript can be accepted so please prepare them now, if you have not already uploaded them. Please carefully read our guidelines for how to prepare and upload this data: https://journals.plos.org/plosbiology/s/figures#loc-blot-and-gel-reporting-requirements

*Protocols deposition*

To enhance the reproducibility of your results, we recommend that if applicable you deposit your laboratory protocols in protocols.io, where a protocol can be assigned its own identifier (DOI) such that it can be cited independently in the future. Additionally, PLOS ONE offers an option for publishing peer-reviewed Lab Protocol articles, which describe protocols hosted on protocols.io. Read more information on sharing protocols at https://plos.org/protocols?utm_medium=editorial-email&utm_source=authorletters&utm_campaign=protocols

Thank you again for your submission to our journal. We hope that our editorial process has been constructive thus far, and we welcome your feedback at any time. Please don't hesitate to contact us if you have any questions or comments.

Sincerely,

Melissa

Melissa Vazquez Hernandez, Ph.D.

Associate Editor

PLOS Biology

mvazquezhernandez@plos.org

-----------------------------------------

REVIEWERS' COMMENTS

-----------------------------------------

Reviewer #1:

This manuscript presents a compelling comparative analysis of stress granules (SGs) induced by MHV and SFV infection, employing sophisticated proximity labeling and proteomic approaches. The central finding—that MHV induces atypical, non-antiviral SGs—is significant and challenges the prevailing paradigm of SGs as universal antiviral hubs. The work is technically sound and well-executed. However, several key concerns must be addressed to solidify the major conclusions and fully contextualize the findings within the field.

Major Comments

1.Experimental Validation of Early SG Suppression by MHV: The conclusion that MHV prevents SG formation early in infection is potentially confounded by the use of a single viral inoculum (MOI=10). To robustly support this claim, infection with a higher viral dose (e.g., MOI=100) must be performed. The number of SGs formed at the same early time point under both conditions should be statistically compared and the viral inocula should be precisely quantified (e.g., in TCID50).

2.Demonstration of Viral RNA Exclusion from MHV SGs: The hypothesis that MHV evades innate immunity by excluding its vRNA from SGs is a cornerstone of the paper. This requires direct experimental validation. Immunofluorescence experiments co-staining for vRNA and SG markers (e.g., G3BP1) must be added, with quantitative co-localization statistics presented (e.g., in a histogram format in a supplemented Figure 7). This is critical evidence to visually and statistically demonstrate the exclusion.

3.Physiological Relevance of SFV NSP3-G3BP1 Interaction: The proposed mechanism for SFV—that NSP3-mediated SG formation is antiviral—requires functional validation. To distinguish these as "classic" antiviral SGs from MHV's atypical ones, the authors should overexpress SFV NSP3 and G3BP1 and measure the subsequent impact on viral replication (e.g., through viral titer or RNA copy number assays). Demonstrating that this interaction partially inhibits viral growth would provide crucial physiological evidence for its antiviral role.

4.Definition and Discussion of Atypical MHV SGs: It remains unclear if the MHV-induced structures are bona fide SGs or a distinct class of virus-specific RNP condensates. The discussion must be expanded to directly address this point, explicitly contrasting the proteome and proposed function of MHV SGs with the canonical, antiviral SGs formed during Sendai virus infection. The discussion should focus on the discovery of these non-immunogenic SGs and their significance for viral immune evasion.

Minor Comments

1.The proteomic data reveals a fundamental difference: SFV RNA is incorporated into SGs while MHV RNA is excluded. The manuscript should discuss the potential mechanism for this selectivity. Is it determined by intrinsic features of the viral RNAs (e.g., sequence, structure) or is it actively mediated by specific viral proteins?

2. The discussion should address the potential link between the temporal differences in SG assembly (early vs. late) and viral replication strategies. The authors are encouraged to discuss whether this timing reflects an active viral strategy to evade or suppress host translation and innate sensing at critical phases of the infection cycle.

3.Figure 1: The green fluorescent signals observed in the 17Cl1 wild-type negative control cells in Figure 1 require explanation. The authors must clarify the source of this signal (e.g., autofluorescence, non-specific antibody binding) to ensure proper interpretation of the data.

4.The legend for Figure 4 is incomplete and must be completed to provide all necessary information for the reader to interpret the figure accurately.

-----------------------------------------

Reviewer #2:

Lang et al present a detailed examination of stress granules, induced by two viruses, mouse hepatitis coronavirus (MHV) and Semliki Forest alphavirus (SFV). Controls include sodium arsenite, a standard treatment used to induce stress granules. Stress granules proteins are identified by fusing the major stress granule initiator protein G3BP1 with the peroxidase APEX, which allows for a very short time period of labeling of the vicinal proteins. The SILAC labeling technique is used to identify the differences between the compared conditions. Additionally, fluorescence microcopy is used to verify the behavior of the major stress granules proteins in cells. SFV induces stress granules early in infection, as previously shown by Gerald McInerney and others, and the granules are soon disassembled. Interestingly, MHV induces stress granules late in infection, when the virus titer is reaching its peak, and these granules are also disassembled. Nicely, Lang et al examine the stress granule microenvironment at three different time point during infection: before the granules appear, at peak granule time point, and after visible granules have disappeared. Importantly, the results are similar for both viruses before the granules appear. Then, SFV induces a strong stress response, which resembles arsenite induces stress, and many known stress granule proteins are found in the vicinity of G3BP1. MHV appears to induce a weaker stress response, and a smaller number of known stress proteins are found. Additionally, many new proteins are identified that can now be linked to MHV-induces stress and could also play general roles. The functions of stress granules during virus infection continue to be debated, even for the well-studied SFV. Interestingly, due to the different time scales, SFV granules could perhaps be linked to virus replication and nonstructural proteins, and MHV granules could perhaps be linked to the large-scale production of structural proteins. Furthermore, Lang et al find that viral RNA is present in the SFV-induced granules but absent from the MHV-induced granules.

Specific comments:

1. The G3BP1-APEX-GFP fusion protein appears to be expressed in addition to the cellular G3BP1 in the cell line constructed for this work. As background information, how does the expression level of the fusion protein compare to that of endogenous G3BP1? Overexpression could conceivably affect some properties of the stress granules, but one is reassured by the fact that the time courses of SG formation and disappearance during virus infections are similar to previously published work.

2. Line 204 glycosylation. Is this set of proteins specifically involved in cytoplasmic O-GlcNAcylation of proteins? And not involved in the glycosylation of secretory proteins, which takes place in compartments topologically separated from the cytoplasmic stress granules and G3BP1.

Minor:

1. Refer to all supplementary figures and other material in the text, in the order the figures (etc.) are numbered. Some might now be missing.

2. Corrections to Figure legends:

-Fig. 4 panels E and F missing

-Fig. 6 check panels B and C for order

-S1 Movie check the wording of the title

-----------------------------------------

Reviewer #3:

In this manuscript, the authors explore the composition of stress granules (SGs) formed during mouse hepatitis virus (MHV). Leveraging APEX2-based proximity labeling combined with quantitative proteomics, they first compare MHV-induced SGs to those triggered by arsenite, a commonly used inducer of oxidative stress. To uncover virus-specific characteristics, a parallel comparison is made with SGs formed during infection by Semliki Forest virus (SFV), another positive-sense single-stranded RNA virus. Their results reveal both stress- and virus-specific differences in SG composition, underscoring the unique nature of virus-induced SGs, including distinct patterns of viral protein and viral RNA (genomic and/or subgenomic) colocalization. The study concludes by examining temporal changes in SG composition throughout the infection.

This study is thoughtfully designed and rigorously executed. A major strength of the manuscript is its direct comparison of different viruses within the same cellular context, utilizing an APEX-based proximity labeling approach combined with SILAC mass spectrometry to reveal virus-specific differences in SG composition would provide valuable insights into the interplay between the viruses and the cellular stress response. The following points may help to strengthen the manuscript.

- The mouse cell line 17Cl1:G3AG used in this study exhibits SG-like foci even under mock (unstressed) conditions, as shown in Video S1. Since overexpression of G3BP1 frequently induces spontaneous self-condensation in the absence of stress, this phenomenon could potentially bias the proteomic enrichment analysis, leading to an underestimation of proteins enriched relative to mock. How many cells display spontaneous SG formation? Assuming this proportion is minimal, the authors should address and discuss the potential impact of this effect on their results.

- The image chosen for the mock condition in panel 1D gives the impression that G3BP1 levels are substantially lower under unstressed conditions. However, this appears consistent with the levels observed in panel 1C in 17Cl1 wild-type cells. Can the authors confirm whether this mock control is the appropriate match for comparison?

- The authors use Markmiller et al. (2018) as a reference for identifying SG core and associated proteins, likely due to the shared methodology. However, other APEX-based studies, such as Marmor-Kollet et al. (2020) (PMID: 33217318) and Qin et al. (2023) (PMID: 37385249). All used different cell types and stress conditions. Some proteins categorized here as "non-SG associated," may appear in other studies using similar or different methodological approaches. The authors should clarify that these proteins were not detected in the Markmiller et al. dataset specifically, rather than generally excluding them from SG association. Comparison could also be made with the highest-confidence stress granule proteins listed in the RNA Granule Database (PMID: 31626750 and PMID: 36662637), which likely includes a broader range of candidate proteins.

- The authors observed that 144 known SG-associated proteins did not meet significance criteria under arsenite treatment, and 164 SG-associated proteins were not enriched in MHV-infected cells. Several studies have proposed that SG seeds exist under normal conditions and expand into larger assemblies upon stress. This model could explain the lack of significant enrichment observed, which warrants further discussion.

Figure 3: While the figure and Venn diagrams are clearly presented and effectively highlight candidate proteins, it is difficult to follow the corresponding categories referenced in the main text. Some categories, such as "stress independent MHV," are unclear. Additionally, viral proteins appear both in the "novel candidates MHV" group and in "MHV enriched (not SG reference)." The authors should clarify these categories and their distinctions to improve clarity.

- Are the MHV viral proteins found in SGs dispensable for replication?

- The comparison of MHV and SFV replication kinetics and ISR activation is insightful, showing that although both viruses follow similar replication dynamics, MHV replicates at lower levels (based on infectious particle release, almost 2 logs difference) and induces less eIF2α phosphorylation (Fig. 4E and 4F). However, the authors do not address the potential relationship between the amount of dsRNA generated during viral replication and PKR activation. Is PKR phosphorylated during these infections? Additionally, is the level of dsRNA lower in MHV-infected cells compared to SFV-infected cells? Such differences could help explain the observed dynamics. Are the numbers of infected cells comparable between the two viruses? Furthermore, how many infected cells exhibit stress granules at various times post-infection (hpi)? Providing this information would strengthen the conclusions and offer a more comprehensive understanding.

- An intriguing finding is the absence of key translation-related proteins such as eIF3a/c and eIF4GI, major components of the translation machinery. Generally, quantifying the correlation either by Pearson correlation or Manders' coefficient would strengthen the colocalization conclusions. The authors do not discuss potential differences in translation strategies between MHV and SFV. Specifically, does SFV recruit distinct translation initiation complexes for genome translation compared to MHV?

- The co-localization of SFV genomic RNA within SGs is observed transiently, specifically at 5 hours post-infection (hpi) (Fig. 8C). This timing is notable but remains unexplored. Interestingly, this timeframe coincides with the beginning of the exponential phase of viral release. Given that previous studies—and the authors' own discussion—indicate that SGs induced by MHV and SFV do not exhibit an antiviral function, it would be valuable to investigate this further and inhibit SG condensation, for example using commercially available G3BP1 inhibitors such as G3Ia or G3Ib. Assessing how the disruption of SG formation and the consequent absence of SFV genomic RNA localization within SGs affects the viral life cycle could provide important mechanistic insights.

- Consistent with this observation, UBAP2L appears to be enriched in SFV-induced stress granules at 5 hours post-infection but is markedly depleted by 9 hours post-infection (Fig. 5B and 5C). This raises the question of whether UBAP2L functions as a host dependency factor or a restriction factor. Silencing UBAP2L could provide valuable insights into the dynamic interplay between stress granule formation and viral replication strategies.

- This work represents a valuable resource for the community; however, no statement regarding data availability was provided. In the interest of FAIR data principles, the authors should feel encouraged to deposit their mass spectrometry dataset in a public repository such as PRIDE to ensure accessibility and reuse.

Minor:

The term SG "disassembly" is more accurate rather than "dissolvement."

Line 172: Reference to Supplementary Table S1 is missing.

SG composition changes over time. The comparison between MHV and SFV may be influenced by differences in SG maturation stages. The authors should cite the work by Hu et al. (2023) (PMID: 38012130), which discusses compositional differences between early and late SGs formed under the same stress conditions.

Figs S10 and S11: show each stain individually for better clarity and the merge.

Fig S12: show the dsRNA stain.

-----------------------------------------

Reviewer #4:

Lang et al. set out to elucidate the compositional diversity of stress granules (SGs) within different infection context, specifically during MHV and SFV infection. For this they generated a recombinant mouse fibroblast 17C11 cell line stably expressing a G3BP1 fused to APEX2 and eGFP called G3AG. Initially the performed proximity labelling in mock, arsenite and MHV-infected cells. Multiple comparisons revealed an overall decrease of SG proteins and translation factors within MHV-induced granules compared to arsenite-induced SGs. Further comparisons to SFV-induced granules showed that their composition resemble arsenite-induced SGs more than MHV-induced granules. The authors observed that while SFV induces strong phosphorylation of eIF2α, MHV did not, which might relate to the differences seen in SG composition. The authors further compared the G3BP1 microenvironment at different timepoints of SG assembly during viral infection. For SFV-induced SGs they observed high similarities between the G3BP1 microenvironment at 'peak SG' and 'post SG' timepoints. The 'weaker' enrichment of eIFs in MHV granules (as suggested in the proteomics data) is supported by IFA staining for different eIFs. Further, the authors observe a stronger cytoplasmic localisation of hnRNPs to the cytoplasm during SFV infection. Finally, the authors perform smRNA-FISH staining to show differences in viral RNA localisation patterns between MHV and SFV infection.

Overall, the study described a novel cell line G3AG, which could prove useful for further studies focusing on SGs. The authors perform thorough comparisons of SG proteomics induced by chemical stress, MHV and SFV infection. However, they fail to convincingly validate their proteomics data and further don't provide a functional characterisation of the observed SGs. Therefore, the authors are encouraged to provide more experimental data to substantiate the conclusions drawn in this manuscript and further show the value of the presented proteomics data for our understanding of SGs during viral infection.

Major comments:

> Can the authors provide experiments addressing the functional relevance of SGs during viral infection. Does depletion or stabilization of SGs have any effect on viral replication or cell survival?

> For the IFA experiment on MHV-infected cells, cells were treated with HR2 peptide to prevent syncytia formation. Can the authors please mention this within the main text and comment if prevention of syncytia formation has any effect on SG formation during MHV infection?

> Re Fig 2 B: the authors suggest that MHV induces a weaker stress response than arsenite leading to less accumulation of SG proteins and translation factors to MHV-induced granules. For SARS-CoV2 it is known that it induces a strong translation shut-off on infected cells via the action of Nsp1. Are some host cell proteins at 8hpi in MHV-infected cells less abundant leading to lower enrichment during down-stream processing? Can the authors provide a western blot of major SG proteins of mock, arsenite treated cells, MHV (8hpi) and SFV (5hpi). Alternatively, it might be that the granules differ in their fluidity and therefore exchange rate with the cytoplasmic environment. Can the author provide FRAP experiments addressing the biophysical properties of the observed granules? Can the authors provide quantification of the SG size in MHV-infected cells compared to SFV-infected and arsenite-treated cells?

> Re Fig 3 and 5: Could the authors provide and discuss comparisons of the here generated proteomics data for arsenite, MHV- and SFV-induced granules with previously published proteomics data for SGs, paracrine granules and P-bodies?

> Fig 3 B: Can the authors show that selected MHV-induced SG interactors are indeed localizing to SGs and do these interactors have anti- or pro-viral properties?

> Fig 3B: can the authors speculate why in MHV-induced SGs they find SG nucleators (Caprin1) and at the same time disassembly factors (USP10), as these are competing for G3BP1 interaction.

> Fig 3 C: does any of the MHV proteins localize to SGs as suggested by the proteomics data? Can the authors provide IFA experiments?

> Fig 4 B: The authors speculate that SG disassembly during SFV depends on the action of the viral nsP3. Can the authors provide a WB showing that nsP3 expression co-occurs with SG disassembly? Further, potentially the disassembly observed during MHV and SFV-infection occurs as a by-production of the activation of intrinsic cell death pathways. Can the authors comment on when cells become apoptotic upon viral infection. Do the authors still observe SG disassembly if cells are treated with caspase inhibitors?

> Fig 4 D: Can the authors provide a quantification of the percentage of cells displaying SGs? Can the authors comment what effect 1.5h cycloheximide treatment has on a viral infection that is 5 or 8 hours long respectively. For MHV the cell seems to be less infected with might be the actual reason that no SGs are formed. Can the authors provide an alternative experiment to prove that the observed virus-induced SGs resemble canonical SGs?

> Fig. 4 E and F: There is no description provided in the figure legends for panel E and F. Further, can the authors add statistics to panel F and adjust y-axis labelling to reflect that panel F refers to eIF2α phosphorylation.

> Fig 4 E: The authors observe lower phosphorylation of eIF2α in MHV-infected cells compared to SFV-infected and arsenite-treated cells. Does this correlate with activation of PKR and subsequent translation shut-off? If translational activity is retained in MHV-infected cells, that might explain why eIFs are less enriched in MHV-induced granules compared to arsenite or SFV-induced granules, as further observed in figure 7 A-D. Does MHV potentially induce SG formation via eIF2α- independent pathways? If yes, the differences observed in SG composition might reflect general differences of SGs induced by eIF2α-dependent vs. independent pathways instead of virus dependent differences.

Re Fig. 6 A-C: The top 5 enriched pathways are shown for MHV and SFV peak and post SGs. However, no enriched pathway is shown for MHV peak SGs. The authors mention in line 354-356 that due to the only few enriched proteins for SG peak and SG post time points, the meaningfulness of this analysis is limited. However, if that is the case, can the authors please clarify the difference to the MHV enriched pathways depicted in figure 2 D and the MHV peak SGs pathways in figure 6 A-C.

> Fig 7 E-G, S10-12: The authors found FUS, PTBP1 and TAF15 enriched in SFV-induced SGs and conclude that this is based on the relocalisation of these proteins to the cytoplasm which is different from the predominant nuclear signal in MHV-infected and arsenite-infected cells. However, this relocalisation only becomes significant at 7hpi representing a late timepoint in SFV-infection, where SGs already start to disassemble. For MHV the authors only tested one timepoint (assumingly 8 hpi, please provide information in the figure legend). Could the authors provide quantitative data on the FUS, PTBP1 and TAF15 localisation during late timepoints of MHV infection to provide stronger evidence that this relocalisation is truly virus-dependent and not due to cell damage?

> Fig. 8 C: The authors mention in the discussion, in line 502-503 that 'SFV shows a strong accumulation of gRNA in these granules', although in Fig. 8 C it is demonstrated, that the viral gRNA is located at the periphery of these granules. Can the authors speculate why the polyadenylated SFV gRNA localises to stress granules differently than polyadenylated cellular RNA and if this has implication for viral infection?

> Within the discussion the authors should speculate on the function of SGs during SFV and MHV infection based on the pathways they identified and on previous research done in viral infections in cells depleted for SG proteins.

Could the authors speculate why they fail to observe enrichment of pattern recognition receptors in their granules? Could the choice of cell line used for infection have an influence on the activation of innate immune signalling pathways and therefore localisation of pattern recognition receptors to SGs? Could the authors speculate on possible limitations of the study as using the G3AG cells what is probed is the microenvironment of G3BP1 which might differ to a certain degree from the microenvironment of SGs.

Minor comments:

- Re Fig. 8: The authors use an MOI of 0.1 instead of 10, which was used in most of the other experiments. Can the authors please comment on the reasoning of this decision and if a lower MOI does affect the kinetics of SG assembly.

- Fig. 2, panel A-C: use same cell labelling as in Sppl. Fig. 2 to reflect that all presented conditions underwent APEX2-mediated proximity labelling (e.g. Fig. 2A displays mock APX+ vs. Arsenite, change to mock APEX+ vs. Arsenite APEX+)

- Re Fig. 8: provide probe sequences used

- Some of the reference numbers cited in the text have different font styles (see line 484, 487, 618, 709)

- Material and Methods- Cell culture: provide culture conditions for BHK21 cells (which are used in line 568); Adjust line spacing of that paragraph to fit the rest of the text.

- Line 590: provide a short description of the TCID50 assay

- Line 601: please clarify if for the arsenite control (treatment 30 min) cycloheximide (treatment for 1.5h) was added before stress granule induction with arsenite.

- Line 603: missing 'C' for '..PBS at 4˚ until….;

- 646-648; 667-675: keep spacing between number and unit consistent

- Line 693: add spacing between brackets

- Line 695-703: text is aligned left, make consistent with rest of the text

- Line 721: spelling error: 'imputed'

- Line 736: spelling error: 'lates'

# Author comment: Author response to Decision Letter 2

**DOI:** 10.1371/journal.pbio.3003363.r003

8 May 2026

Attachment Submitted filename: Answers_to_Reviewer_Comments_final.pdf

# Peer review: Decision Letter 2

**DOI:** 10.1371/journal.pbio.3003363.r004

**Authors:** Melissa Vazquez Hernandez

15 Jun 2026

Dear Volker,

I hope you are doing great and will have a nice time during the conference. Thank you for your patience while we considered your revised manuscript "Stress granules formed during different RNA virus infections show remarkable plasticity and substantial virus-specific differences in their formation and composition" for publication as a Research Article at PLOS Biology. This revised version of your manuscript has been evaluated by the PLOS Biology editors, the Academic Editor and the original reviewers.

Based on the reviews, we are likely to accept this manuscript for publication, provided you satisfactorily address the remaining editorial points. Please also make sure to address the following data and other policy-related requests.

1) We routinely suggest changes to titles to ensure maximum accessibility for a broad, non-specialist readership, and to ensure they reflect the contents of the paper. In this case, we would suggest a minor edit to the title, as follows. Please ensure you change both the manuscript file and the online submission system, as they need to match for final acceptance:

"Stress granules formed during infection with RNA viruses have significant virus-specific differences in generation and composition"

2) Please add the weblink of the funding agencies in the Financial Disclosure statement in the manuscript details and during re-submission.

3) You may be aware of the PLOS Data Policy, which requires that all data be made available without restriction: http://journals.plos.org/plosbiology/s/data-availability. For more information, please also see this editorial: http://dx.doi.org/10.1371/journal.pbio.1001797

Please supply the numerical values either in the a supplementary file or as a permanent DOI’d deposition for the following figures:

Figure 2A-D, 4BCEG, 5, 6, 7H, 8A-DF, 9BC, 10BC-F, S3, S5AB, S6, S7D, S8, S9A-G, S11B, S12B, S13B, S15, S16ABC, S17AB

NOTE: the numerical data provided should include all replicates AND the way in which the plotted mean and errors were derived (it should not present only the mean/average values).

*I am aware that in your availability statement you say "Image analysis scripts are available on the Open Science Framework under the identifier DOI 10.17605/OSF.IO/7QFG6." However, I currently have no access to it (please make sure to make it available), and we also require the raw data for all the figures mentioned above.

4) Please cite the location of the data clearly in all relevant main and supplementary Figure legends, e.g. “The data underlying this Figure can be found in S1 Data” or “The data underlying this Figure can be found in https://doi.org/10.5281/zenodo.XXXXX”

5) For figures containing FACS data (Figures S14ABC), please provide the FCS files and a picture showing the successive plots and gates that were applied to the FCS files to generate the figure. Unfortunately, is seems that FlowRepository (https://flowrepository.org/) is not longer accessible. However, you can upload the files to our system or to a publicly available repository like Zenodo, and provide the accession number/URL of the deposition in the Data Availability Statement in the online submission form.

6) Supplementary files (e.g., excel). Please ensure that all data files are uploaded as 'Supporting Information' and are invariably referred to (in the manuscript, figure legends, and the Description field when uploading your files) using the following format verbatim: S1 Data, S2 Data, etc. Multiple panels of a single or even several figures can be included as multiple sheets in one excel file that is saved using exactly the following convention: S1_Data.xlsx (using an underscore).

7) Thank you for having the scale bar in the microscopy pictures. However, I notices that some figures (like S15) has numbers in on top of the scale bars, while other pictures do not.

8) Please ensure that your Data Statement in the submission system accurately describes where your data can be found and is in final format, as it will be published as written there. Also make sure that this is open and it can be accessed.

9) Per journal policy, if you have generated any custom code during the course of this investigation, please make it available without restrictions. Please ensure that the code is sufficiently well documented and reusable, and that your Data Statement in the Editorial Manager submission system accurately describes where your code can be found. More information on our Code Policy, what and how to share can be found here: https://journals.plos.org/plosbiology/s/code-availability

Please note that we cannot accept sole deposition of code in GitHub, as this could be changed after publication. However, you can archive this version of your publicly available GitHub code to Zenodo. Once you do this, it will generate a DOI number, which you will need to provide in the Data Accessibility Statement (you are welcome to also provide the GitHub access information). See the process for doing this here: https://docs.github.com/en/repositories/archiving-a-github-repository/referencing-and-citing-content

As you address these items, please take this last chance to review your reference list to ensure that it is complete and correct. If you have cited papers that have been retracted, please include the rationale for doing so in the manuscript text, or remove these references and replace them with relevant current references. Any changes to the reference list should be mentioned in the cover letter that accompanies your revised manuscript.

In addition to these revisions, you may need to complete some formatting changes, which you will receive in a follow up email. A member of our team will be in touch with a set of requests shortly. If you do not receive a separate email within a few days, please assume that checks have been completed, and no additional changes are required.

We expect to receive your revised manuscript within three weeks.

To submit your revision, please go to https://www.editorialmanager.com/pbiology/ and log in as an Author. Click the link labelled 'Submissions Needing Revision' to find your submission record. Your revised submission must include the following:

- a cover letter that should detail your responses to any editorial requests, if applicable, and whether changes have been made to the reference list

- a Response to Reviewers file that provides a detailed response to the reviewers' comments (if applicable, if not applicable please do not delete your existing 'Response to Reviewers' file.)

- a track-changes file indicating any changes that you have made to the manuscript.

NOTE: If Supporting Information files are included with your article, note that these are not copyedited and will be published as they are submitted. Please ensure that these files are legible and of high quality (at least 300 dpi) in an easily accessible file format. For this reason, please be aware that any references listed in an SI file will not be indexed. For more information, see our Supporting Information guidelines:

https://journals.plos.org/plosbiology/s/supporting-information

*Published Peer Review History*

Please note that you may have the opportunity to make the peer review history publicly available. The record will include editor decision letters (with reviews) and your responses to reviewer comments. If eligible, we will contact you to opt in or out. Please see here for more details:

https://plos.org/published-peer-review-history/

*Press*

Should you, your institution's press office or the journal office choose to press release your paper, please ensure you have opted out of Early Article Posting on the submission form. We ask that you notify us as soon as possible if you or your institution is planning to press release the article.

*Protocols deposition*

To enhance the reproducibility of your results, we recommend that if applicable you deposit your laboratory protocols in protocols.io, where a protocol can be assigned its own identifier (DOI) such that it can be cited independently in the future. Additionally, PLOS ONE offers an option for publishing peer-reviewed Lab Protocol articles, which describe protocols hosted on protocols.io. Read more information on sharing protocols at https://plos.org/protocols?utm_medium=editorial-email&utm_source=authorletters&utm_campaign=protocols

Please do not hesitate to contact me should you have any questions.

Sincerely,

Melissa

Melissa Vazquez Hernandez, Ph.D.

Associate Editor

mvazquezhernandez@plos.org

PLOS Biology

------------------------------------------------------------------------

REVIEWERS' COMMENTS

------------------------------------------------------------------------

Reviewer #1: The authors have addressed my previous concerns

------------------------------------------------------------------------

Reviewer #2: The authors have provided a comprehensive revision of the manuscript, addressing my concerns and many others. In the manuscript, Lang et al present a detailed examination of stress granules, induced by two viruses, mouse hepatitis coronavirus (MHV) and Semliki Forest alphavirus (SFV). Controls include sodium arsenite, a standard treatment used to induce stress granules. In the revision, more work and discussion are devoted to the finding that viral RNA is present in the SFV-induced granules but absent from the MHV-induced granules. The same finding with respect to the two viral RNAs is now reported even in cells co-infected with the two viruses, showing that specific exclusion of MHV RNAs is likely.

------------------------------------------------------------------------

Reviewer #3: The authors have made substantial and thoughtful revisions that have clearly strengthened the manuscript. The data are robust and well presented. The responses to my comments are thorough and constructive. Where certain suggested experiments were not feasible, the authors have provided appropriate justification and, where possible, even implemented alternative approaches to support and further validate their conclusions. Overall, the revised manuscript is of high quality and represents a meaningful contribution that will be of broad interest to the scientific community.

------------------------------------------------------------------------

Reviewer #4: The authors have performed a significant amount of additional experimental work to address a large range of comments from all reviewers. While some comments were not answered with new data - justification was provided for doing so. These additional data have now substantially improved the quality of the manuscript which together with revised points of discussion support publication in PLOS Biology.

# Author comment: Author response to Decision Letter 3

**DOI:** 10.1371/journal.pbio.3003363.r005

30 Jul 2026

Attachment Submitted filename: Answers_to_Reviewer_Comments_final_auresp_3.pdf

# Editor report: Decision Letter 3

**DOI:** 10.1371/journal.pbio.3003363.r006

**Authors:** Melissa Vazquez Hernandez

3 Aug 2026

Dear Volker,

Thank you for the submission of your revised Research Article "Stress granules formed during infections with RNA viruses have significant virus-specific differences in generation and composition" for publication in PLOS Biology. On behalf of my colleagues and the Academic Editor, Andrew Mehle, I am pleased to say that we can in principle accept your manuscript for publication, provided you address any remaining formatting and reporting issues. These will be detailed in an email you should receive within 2-3 business days from our colleagues in the journal operations team; no action is required from you until then. Please note that we will not be able to formally accept your manuscript and schedule it for publication until you have completed any requested changes.

Please take a minute to log into Editorial Manager at http://www.editorialmanager.com/pbiology/, click the "Update My Information" link at the top of the page, and update your user information to ensure an efficient production process.

PRESS

We frequently collaborate with press offices. If your institution or institutions have a press office, please notify them about your upcoming paper at this point, to enable them to help maximise its impact. If the press office is planning to promote your findings, we would be grateful if they could coordinate with biologypress@plos.org. If you have previously opted in to the early version process, we ask that you notify us immediately of any press plans so that we may opt out on your behalf.

We also ask that you take this opportunity to read our Embargo Policy regarding the discussion, promotion and media coverage of work that is yet to be published by PLOS. As your manuscript is not yet published, it is bound by the conditions of our Embargo Policy. Please be aware that this policy is in place both to ensure that any press coverage of your article is fully substantiated and to provide a direct link between such coverage and the published work. For full details of our Embargo Policy, please visit http://www.plos.org/about/media-inquiries/embargo-policy/.

Thank you again for choosing PLOS Biology for publication and supporting Open Access publishing. We look forward to publishing your study.

Sincerely,

Melissa

Melissa Vazquez Hernandez, Ph.D., Ph.D.

Senior Editor

PLOS Biology

mvazquezhernandez@plos.org
